# A Comparative Study and a Phylogenetic Exploration of the Compositional Architectures of Mammalian Nuclear Genomes

**DOI:** 10.1371/journal.pcbi.1003925

**Published:** 2014-11-06

**Authors:** Eran Elhaik, Dan Graur

**Affiliations:** 1 Department of Animal and Plant Sciences, University of Sheffield, Sheffield, United Kingdom; 2 Department of Biology & Biochemistry, University of Houston, Houston, Texas, United States of America; University of California San Diego, United States of America

## Abstract

For the past four decades the compositional organization of the mammalian genome posed a formidable challenge to molecular evolutionists attempting to explain it from an evolutionary perspective. Unfortunately, most of the explanations adhered to the “isochore theory,” which has long been rebutted. Recently, an alternative compositional domain model was proposed depicting the human and cow genomes as composed mostly of short compositionally homogeneous and nonhomogeneous domains and a few long ones. We test the validity of this model through a rigorous sequence-based analysis of eleven completely sequenced mammalian and avian genomes. Seven attributes of compositional domains are used in the analyses: (1) the number of compositional domains, (2) compositional domain-length distribution, (3) density of compositional domains, (4) genome coverage by the different domain types, (5) degree of fit to a power-law distribution, (6) compositional domain GC content, and (7) the joint distribution of GC content and length of the different domain types. We discuss the evolution of these attributes in light of two competing phylogenetic hypotheses that differ from each other in the validity of clade Euarchontoglires. If valid, the murid genome compositional organization would be a derived state and exhibit a high similarity to that of other mammals. If invalid, the murid genome compositional organization would be closer to an ancestral state. We demonstrate that the compositional organization of the murid genome differs from those of primates and laurasiatherians, a phenomenon previously termed the “murid shift,” and in many ways resembles the genome of opossum. We find no support to the “isochore theory.” Instead, our findings depict the mammalian genome as a tapestry of mostly short homogeneous and nonhomogeneous domains and few long ones thus providing strong evidence in favor of the compositional domain model and seem to invalidate clade Euarchontoglires.

## Author Summary

The non-uniformity of DNA composition in mammalian genomes has been known for over four decades. Many attempts have been made to provide a concise description of this heterogeneity and to identify the evolutionary driving forces behind this compositional phenomenology. The first concise description of the genome suggested an isochoric structure according to which the mammalian genome consists of a mosaic of long, compositionally homogenous DNA sequences. With the advent of genome sequencing, this description was found to be inappropriate. We have recently proposed an alternative “compositional domains” model that depicts the human and cow genomes as composed of mixture of compositionally homogeneous and nonhomogeneous domains. Most of these domains are very short. Since its proposal, this model has been validated in plethora of invertebrate genomes. Here, we test the validity of this model on eleven mammalian and avian genomes using seven attributes of compositional domains and discuss their evolution. We also use these attributes to decide between two competing phylogenetic hypotheses. Our findings provide strong supporting evidence for the “compositional domains” model and indicate that rodents are not as close to primates as envisioned by the Euarchontoglires hypothesis.

## Introduction

Human and cow genomes have been shown to possess a complex architecture, in which compositionally homogeneous and nonhomogeneous domains of varying lengths and nucleotide composition are interspersed with one another [1], [2]. These empirically derived compositional architectures are mostly incompatible with the “isochore theory” [3]–[6], according to which the genomes of warm-blooded vertebrates are depicted as mosaics of fairly long isochores —typically 300 kb or more—each possessing a characteristic GC content that differs significantly from that of its neighbors, and each classifiable by GC content into six or less isochore families [7]–[14].

Numerous methods for segmenting DNA sequences into contiguous compositionally-coherent domains have been proposed in the literature. These methods differ from one another in the number and types of parameters used in the segmentation process, as well as in the levels of user intervention. Unfortunately, even methods that limit user input to a few parameters yield incongruent results with one another [15], whereas methods that rely on subjective user intervention [e.g., 16] preclude independent replication of the results and are, thus, unscientific. Through comparison of performances against benchmark simulations, Elhaik, Graur, and Josić [2] identified a segmentation method, D_JS_ [17], that outperformed all others. However, D_JS_ failed to partition sequences with low compositional dispersion and had difficulties in identifying short homogeneous domains. To rectify these inadequacies, Elhaik et al. [15] devised IsoPlotter—a recursive segmentation algorithm that employs a dynamic threshold, which takes into account the composition and length of each segment. Most importantly, IsoPlotter is an unsupervised algorithm, i.e., it requires no subjective user intervention, and through benchmark validation, it was shown to yield unbiased results [15].

The compositional domains identified by IsoPlotter are contiguous genomic segments, each with a characteristic GC content that differs significantly from the GC contents of its adjacent upstream and downstream compositional domains. By comparing the GC content variance of compositional domains with that of the chromosomes on which they reside, compositional domains can be further classified into two types: “compositionally homogeneous domains,” or simply “homogeneous domains,” and “compositionally nonhomogeneous domains” or “nonhomogeneous domains.” A subset of long homogeneous domains, where “long” is arbitrarily defined as ≥300 kb, are termed “isochoric” domains (sensu [12]). By segmenting the human genome with IsoPlotter, we found that one-third of the genome is composed of compositionally nonhomogeneous domains and the remaining is a mixture of many short compositionally homogeneous domains and relatively few long ones [15]. “Isochoric” domains cover less than a third of the human genome. Similar results were obtained for the cow genome [1].

**Figure 1:**
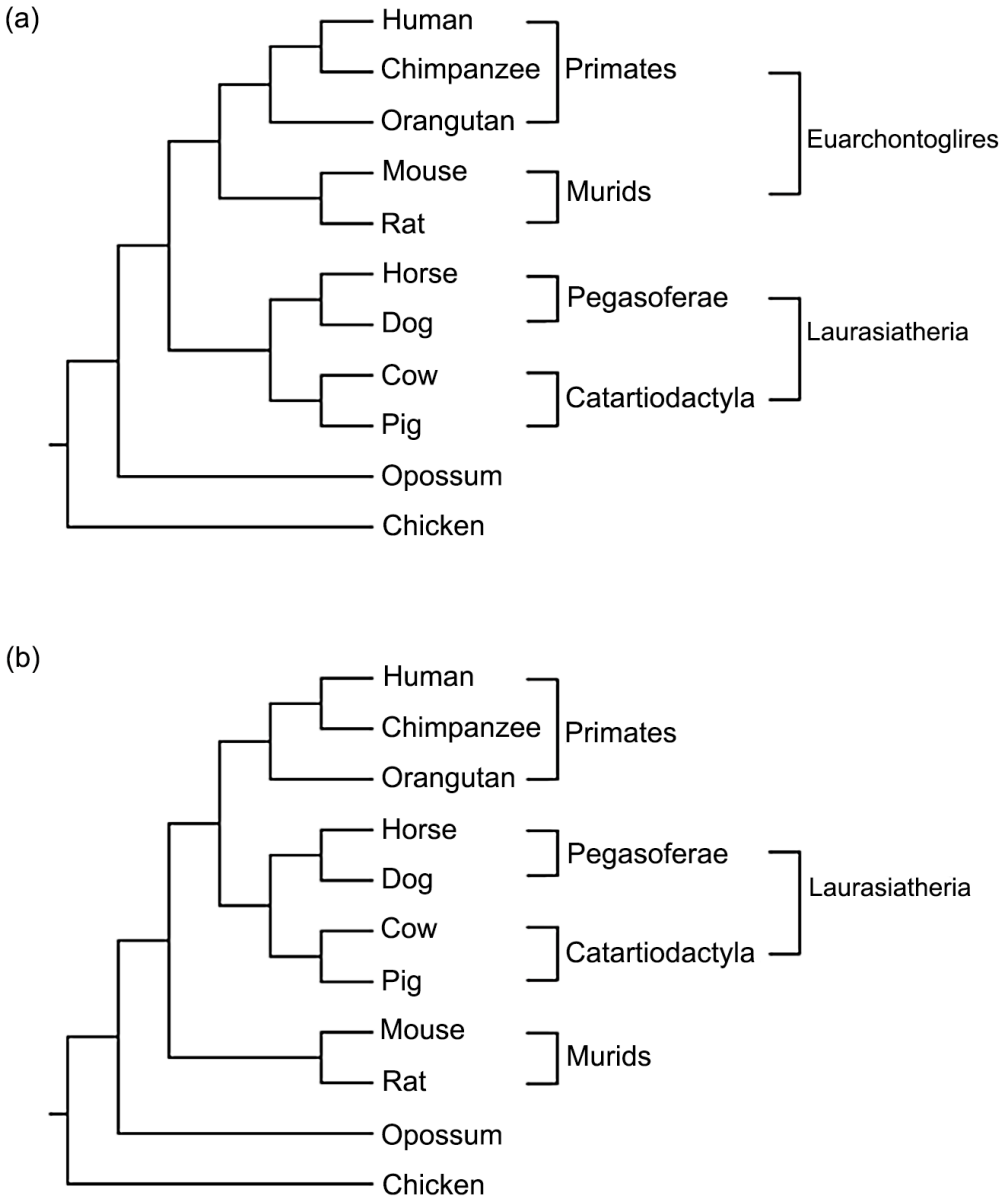
Phylogenetic trees illustrating two competing hypotheses concerning the relative kinships of murids, laurasiatherians, and primates to one another.

Here, we characterize the compositional architecture of ten completely sequenced mammalian genomes and an avian outgroup, and attempt to identify quantitative trends in the evolution of homogeneous and nonhomogeneous domains. Seven attributes of compositional domains are used, many of which were previously used to characterize compositional architectures [1], [18]–[28]. Each genome is defined by: (1) the number of compositional domains, (2) compositional domain-length distribution, (3) density of compositional domains, (4) genome coverage by the different domain types, (5) degree of fit to a power-law distribution, (6) compositional domain GC content, and (7) the joint distribution of GC content and length of the different domain types. Our results are interpreted in light of two currently competing phylogenetic hypotheses depicting the evolution of eutherian mammals for which traditional phylogenetic tools provided ambiguous answers [e.g.], [ 29, 30] (Figure 1). Further, our results support the so-called “murid shift” hypothesis, and suggest that homogeneous and nonhomogeneous domains are biologically different.

This evolutionary study represents a dramatic departure from earlier studies that either extrapolated from a few genes to the entire genome [e.g.], [ 10], [ 31, 32], used unreliable proxies to infer the composition of domains [e.g.], [ 31, 33], or used irreproducible methodologies [e.g.], [ 16, 34]. Our results will be compared with claims made by proponents of the “isochore theory.” Sadly, we are forced yet again to confront the “isochore theory,” because despite its being refuted numerous times [e.g.], [ 18], [ 35], [ 36, 37], proponents of the theory and those invested in it continue to pursue the notion of isochores aggressively, relentlessly, and vociferously [e.g.], [ 31], [ 38], [ 39]–[46]. It seems that T. H. Huxley's dictum on “the great tragedy of science” being “the slaying of a beautiful theory by an ugly fact” does not easily apply to the concept of “isochores.”

## Results

All mammalian genomes in our study are similar in size, ranging from 2 Gb in horse to 3.4 Gb in opossum. At 1 Gb, the size of the chicken genome is considerably smaller than the average mammalian genome. The genomic characteristics of the compositional domains for the 11 species under study are listed in Table 1.

**Table 1:** Genome statistics for compositional, homogeneous, nonhomogeneous, and “isochoric” domains.

	Species	Human	Chimpanzee	Orangutan	Mouse	Rat	Horse	Dog	Cow	Pig	Opossum	Chicken
Whole genome	Sequenced genome size (Gb)	2.8	2.8	2.7	2.6	2.5	2.0	2.3	2.2	2.5	3.4	1.0
	Mean GC content	40.80	40.70	40.70	41.80	41.90	41.10	41.00	41.70	41.80	37.70	41.30
Compositional domains	Number of domains	107,571	107,359	105,688	67,223	63,137	112,350	104,885	96,410	90,881	107,356	39,450
	Domain density (per MB)	38.7	39	38.8	26.3	25.5	48.1	45.3	43.2	38.6	31.5	40.1
	Mean domain length (bp)	25,865	25,637	25,764	38,060	39,233	20,787	22,097	23,144	25,938	31,788	24,965
	Median domain length (bp)	7,808	7,840	7,936	9,216	9,376	7,456	7,744	7,872	7,744	7,616	9,216
	Mean GC content (%)	42.7	42.7	42.6	43	43.3	43.3	43.6	44	44	39.6	43
	Median GC content (%)	41.8	41.8	41.6	42.8	43.1	42	42	43	42.9	38.4	41.7
Homogeneous domains	Number of domains	74,579	73,172	72,384	41,783	38,945	83,169	73,141	70,850	64,228	63,393	28,141
	Fraction out of all compositional domains (%)	69.3	68.2	68.5	62.2	61.7	74	69.7	73.5	70.7	59	71.3
	Domain density (per MB)	26.8	26.6	26.6	16.3	15.7	35.6	31.6	31.8	27.2	18.6	28.6
	Mean domain length (bp)	29,668	29,708	29,820	48,190	50,257	24,027	25,569	26,842	31,292	43,197	28,854
	Median domain length (bp)	8,384	8,480	8,512	10,880	11,264	8,064	8,448	8,576	8,576	8,960	11,104
	Mean GC content (%)	42.3	42.2	42.1	43.5	43.6	42.7	42.7	43.7	43.7	38.7	41.5
	Median GC content (%)	40.8	40.8	40.6	43.3	43.4	40.9	40.8	42.4	42.6	37.4	40.3
	Fraction of genome covered (%)	79.5	79	79.3	78.7	79	85.6	80.7	85.2	85.3	80.2	82.4
	Length of largest domain (Mb)	5.2	6.5	4.3	5.2	7.6	3.8	7.4	4.8	6.1	10.5	4
Nonhomogeneous domains	Number of domains	32,992	34,187	33,304	25,440	24,192	29,181	31,744	25,560	26,653	43,963	11,309
	Fraction out of all compositional domains (%)	30.7	31.8	31.5	37.8	38.3	26	30.3	26.5	29.3	41	28.7
	Domain density (per MB)	11.9	12.4	12.2	9.9	9.8	12.5	13.7	11.5	11.3	12.9	11.5
	Mean domain length (bp)	17,269	16,923	16,949	21,422	21,486	11,553	14,095	12,893	13,037	15,335	15,287
	Median domain length (bp)	6,784	6,784	6,912	7,424	7,456	6,176	6,528	6,464	6,368	6,464	6,496
	Mean GC content (%)	43.7	43.7	43.7	42.4	42.8	44.9	45.6	44.8	44.5	41	46.8
	Median GC content (%)	43.2	43.2	43.1	42.2	42.7	44.4	44.3	44.1	43.8	39.7	46.1
	Fraction of genome covered (%)	20.5	21	20.7	21.3	21	14.4	19.3	14.8	14.7	19.8	17.6
	Length of largest domain (Mb)	2.3	1.9	2.1	7.3	7.5	1.2	2.2	1.3	2	2.9	1.8
“Isochoric” domains	Number of domains	1,071	1,052	1,084	1,312	1,317	811	721	872	1,030	1,705	281
	Fraction out of all compositional domains (%)	1	1	1	2	2.1	0.7	0.7	0.9	1.1	1.6	0.7
	Fraction out of all compositionally homogeneous domains (%)	1.4	1.4	1.5	3.1	3.4	1	1	1.2	1.6	2.7	1
	Mean domain length (bp)	652,728	656,232	651,933	656,374	634,304	587,228	633,831	570,266	675,778	749,900	554,962
	Median domain length (bp)	481,504	485,408	483,488	484,592	480,704	436,864	450,496	455,872	509,888	516,480	421,408
	Mean GC content (%)	38.3	38.3	38.1	40.6	40.7	38.2	37.8	38.8	39.5	36.6	39.4
	Median GC content (%)	37.5	37.5	37.5	39.9	40	37.5	37	38	39	36.4	38.8
	Fraction of genome covered (%)	25.1	25.1	26	33.7	33.7	20.4	19.7	22.3	29.5	37.5	15.8

### Compositional domain abundance

Genome statistics for compositional, homogeneous, nonhomogeneous, and “isochoric” domains are shown in Table 1. In Table S1 we present the same data partitioned by individual chromosomes. The mean number of compositional domains in a mammalian genome in our sample is approximately 96,000, with opossum having the largest number of domains (107,000), and rat having the smallest (∼63,000).

On average, over two thirds of all mammalian domains are homogeneous, but this proportion varies with taxon (Table 1). Opossum has the smallest fraction of homogeneous domains (59%) followed by murids (62%). By contrast, pig (71%) and horse (74%) genomes are the most enriched for homogeneous domains. Isochoric domains constitute only a tiny fraction of the compositional domains, from 0.7% in horse and dog to 2.1% in rat.

### Length distribution of compositional domains

The mean compositional-domain length varies from ∼25,700 bp in primates to ∼38,500 bp in murids (Table 1). The median length is much smaller in all taxa, indicating an extreme skewed distribution towards very short domains. For example, half of the compositional domains in rat are shorter than 9,216 bp. The mean and median lengths of homogeneous and nonhomogeneous domains within a taxon are practically indistinguishable. The largest homogeneous domain among all species is one 10.5-megabase (Mb) long (GC content of 36%) found in the opossum genome. In the human genome, the largest homogenous domain is about half that length (5.2 Mb).

**Figure 2:**
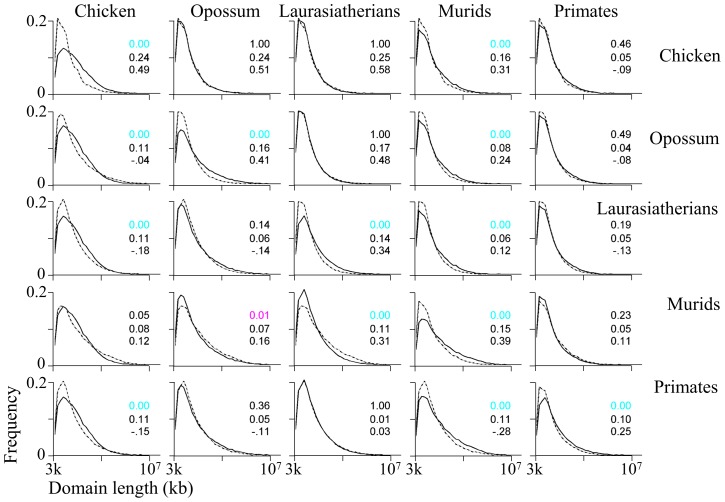
Pairwise comparisons of domain-length distributions for five taxa. Homogeneous-domain lengths are shown above the diagonal; nonhomogeneous-domain lengths are below, where the distribution curves of the species on the X-axis are solid and those on the Y-axis are dashed. On the diagonal we compare homogeneous and nonhomogeneous domain length distributions within a taxon. The first value in each plot is the *p*-value of significant (Kolmogorov-Smirnov goodness-of-fit test) and the colors represent the actual *p*-value after correcting for multiple testing using the FDR method (black>0.05 and pink<0.05). The second and third values are effect size calculated as the nonoverlapping percentage of the two distributions and Cohen's *d* using the Hedges' *g* estimator, respectively.

Almost all the distributions of homogeneous domain lengths in all studied species (Figure 2) are significantly different from each other (Kolmogorov-Smirnov goodness-of-fit test, *p*<0.01), however, this is due to the large sample sizes. The magnitude of the differences between homogeneous and nonhomogeneous domain lengths is very small in all species (area overlap>98%, Cohen's *d*<0.05) with the chicken genome exhibiting borderline similarity (area overlap 97%, Cohen's *d*<0.05).

**Figure 3:**
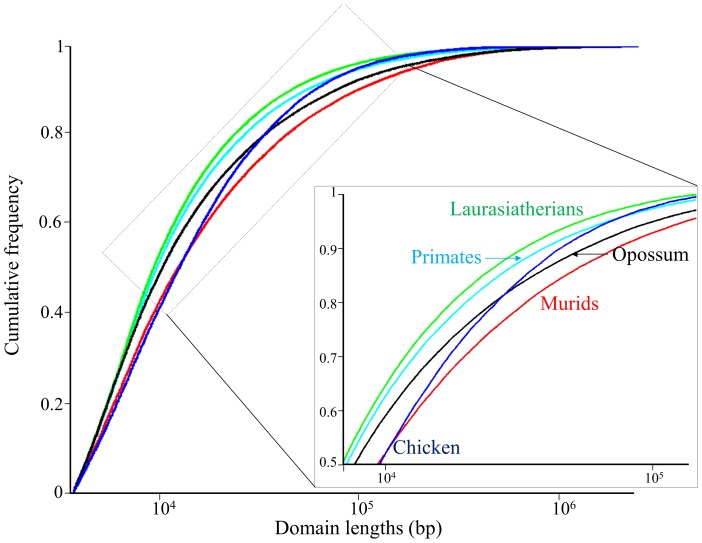
The cumulative distribution of homogeneous domain lengths in log scale. For simplicity, the mean distributions of primates, murids, and laurasiatherians are shown. In the inset, the majority of the domains of medium-short length.

A comparison of the cumulative distributions of domain lengths indicates that the top percentile in murids consists of domains larger than 511 kb, whereas the top percentile in the laurasiatherian genomes consists of domains larger than 281 kb (Figure 3). In mammalian genomes, the proportion of long homogeneous domains (≥300 kb), i.e., “isochoric” domains, out of all domains is 1% and twice that in murids (2.02%). Similar cumulative distributions were observed for compositional and nonhomogeneous domains (Figure S1).

### Compositional domain density

**Figure 4:**
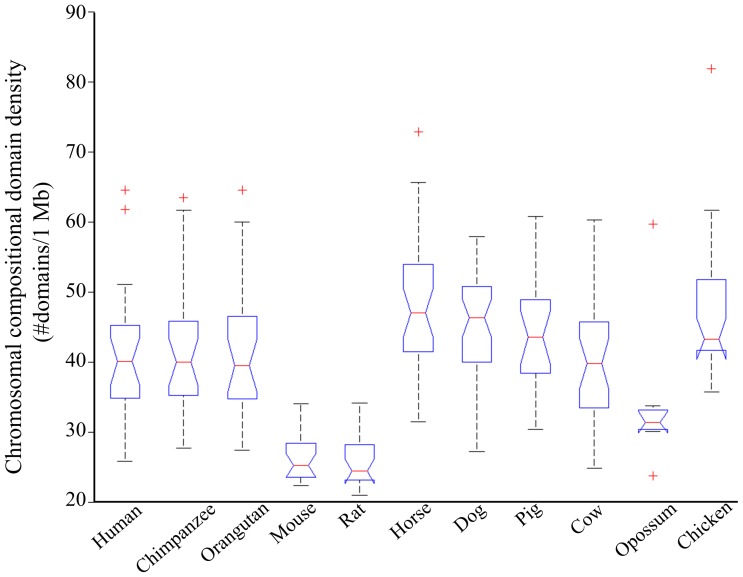
Compositional domain densities of all chromosomes. Box plots summarize medians, quartiles, and range.

Domain density measures the average number of compositional domains per Mb. When divided into GC-poor and GC-rich compositional domains it ranges from 0 to 90 domains/Mb for GC-poor domains and up to 121 domains/Mb for GC-rich domains (Figure 4). Homogeneous domains are more dense for both GC-poor (0–57 domains/Mb) and GC-rich (0–98 domains/Mb) domains compared to nonhomogeneous GC-poor (0–43 domains/Mb) and GC-rich (0–64 domains/Mb) domains, respectively. In regions of low domain densities, the density of GC-rich domains is higher than the density of GC-poor domains. That is, genomic regions with fewer domains are more likely to be GC-rich, whereas denser genomic regions are more likely to harbor GC-poor domains (Figure S2a).

On average, murid chromosomes are the least dense (26 domains/Mb), whereas the horse genome is the most dense (49 domains/Mb). The chromosomal domain densities of opossum are as low as murids for homogeneous domains (21 and 16 domains/Mb, respectively) and as high as primates for nonhomogeneous domains (13 domains/Mb). The overall chromosomal domain densities position opossum (34 domains/Mb) between murids (26 domains/Mb) and other mammals (43 domains/Mb).

Similar patterns were observed when comparing the compositional domain densities of GC- rich and GC-poor domains (Figure S3); the opossum and primate genomes have the highest density for GC-rich domains (21 and 18 domains/Mb, respectively). By contrast, the opossum's genome low density for GC-poor domains (10 domains/Mb) is lower even than that of murids (16 domains/Mb). The overall domain density in opossum (31 domains/Mb) is between that of murids (25.5 domains/Mb) and primates (∼38.5 domains/Mb).

Domain density largely varies among chromosomes and chromosome types. Density differences between chromosomes can reach 100% (Figures 4, S2) with sex chromosomes having a lower density than the average autosome (Table S1). These results indicate that the processes that shaped the inter-chromosomal domain organization acted non-uniformly on all chromosomes and their effect on domain lengths was highly variable in different lineages implying the existence of a compositional constraint on chromosomal heterogeneity.

### Genomic coverage of compositional domains

**Figure 5:**
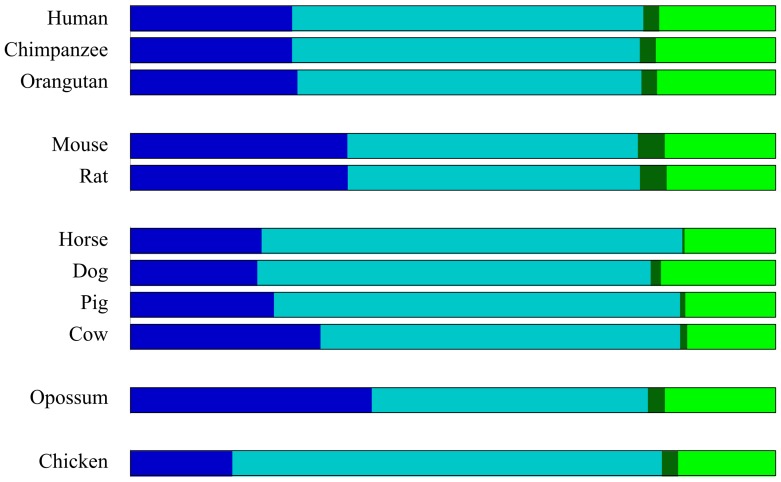
Genomic coverage of four compositional domain types. Homogeneous domains are in blue shades; nonhomogeneous domains are in green shades. Domains longer than 300 kb are in dark shades; domains shorter than 300 kb are in light shades. Compositionally homogeneous domains longer than 300 kb (i.e., isochoric domains) are in dark blue.

In Figure 5, we show the relative genomic coverage of compositional domains as a function of domain homogeneity and length. The genomic coverage by homogeneous domains ranges from ∼79% in primates and murids to ∼85% in horse. By defining “isochoric” domains as compositionally homogeneous domains longer than 300 kb, we find that the genomic coverage by “isochores” in mammals is a trifling 27%, compared to 16% in the chicken. Murids and opossum have the largest genomic coverage by “isochoric” domains (34% and 37%, respectively). Relaxing the “isochore” definition to include homogeneous domains larger than 100 kb, as proposed by Nekrutenko and Li [47], slightly increases the “isochoric” portion of the genome to 38%. These results, in themselves, are sufficient to invalidate the “isochore theory” or at least diminish its applicability.

### Are domain lengths power-law distributed?

**Figure 6:**
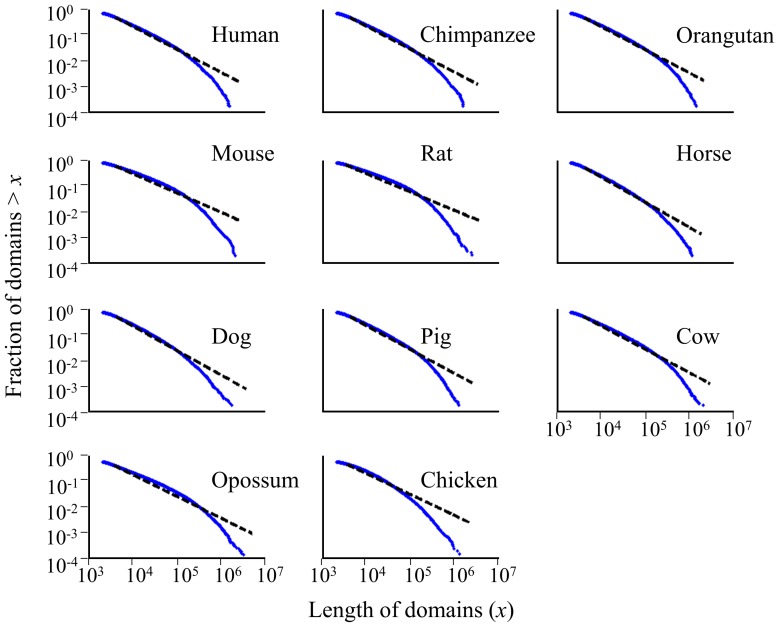
The cumulative density function *P*(x) of compositional homogeneous domain lengths (x) (points) plotted on a log-log scale. The dashed lines represent the maximum likelihood power-law fits to the data.

The distribution of domain lengths in the human genome is commonly depicted as a power-law distribution over a large range of length scales [e.g.], [ 18], [ 48, 49]. A distribution is said to follow a power-law if its histogram is a straight line when plotted on a log-log scale [50], [51]. To gauge the power-law model, we used two approaches: first, we compared the cumulative distributions of homogeneous domain lengths to the maximum likelihood power-law fits. In all cases, the complementary cumulative distribution function *P*(x) and their maximum likelihood power-law fits deviate from a straight line, and the *p*-value is sufficiently small (Kolmogorov-Smirnov, *p*<0.01) that the power-law model can be ruled out (Figure 6). In other words, there is a very small probability that the data can be modeled by a power-law. An even weaker fit was obtained using compositional domains and nonhomogeneous domains (Figure S4). Next, we tested the power-law behavior of domain lengths using the random group formation model. We found that the same deviations from a power-law-like behavior were also predicted by the random group formation model [52] (Figure S5).

The deviations of the data from power-law behavior are caused by the excess of short domains and low frequency of long domains. These findings are at odds with earlier contentions that the mammalian genome is a mosaic of long homogeneous domains with very few short domains [e.g.], [ 12], [ 49, 53]. However, we note that earlier results are not based on the length distribution of actual domains as some authors chose to avoid the excess of short domains – that cause the deviation from power-law – by concatenating them to form artificially long domains [e.g.], [ 54, 55]. We believe that the decision as to whether or not neighboring domains should be concatenated should rely solely on their homogeneity rather than on attempts to make the data fit a preconceived model.

Moreover, if domain lengths are truly drawn from a power-law distribution, the power-law model should fit the data over more than three orders of magnitude [50]. In reality, the power-law fit is quite poor and should thus be rejected (Figures 6, S4, S5). Our findings are in agreement with previous studies that rejected the power-law behavior of compositional domains, although they relied on a small dataset and incomplete genomic sequences [56]–[61]. We reported similar findings in three ant genomes [19]–[21].

### Compositional domain GC content

**Figure 7:**
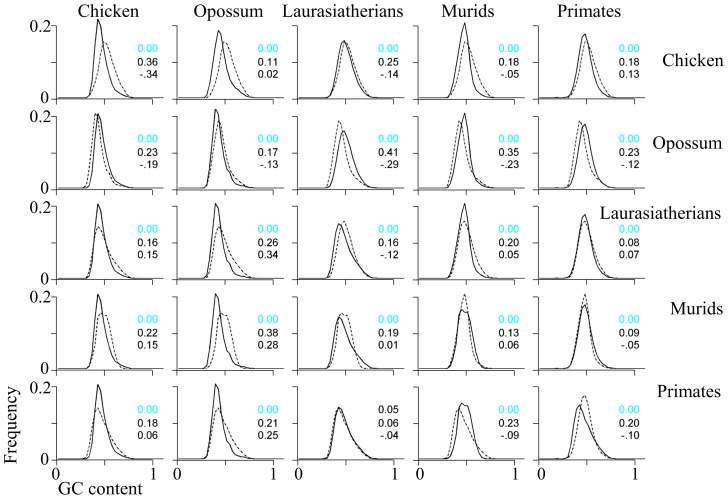
Pairwise comparisons of domain GC content distributions for five taxa. Homogeneous-domain lengths are shown above the diagonal; nonhomogeneous-domain lengths are below, where the distribution curves of the species on the X-axis are solid and those on the Y-axis are dashed. On the diagonal we compare homogeneous and nonhomogeneous domain GC content distributions within a taxon. The first value in each plot is the *p*-value of significant (Kolmogorov-Smirnov goodness-of-fit test) and the colors represent the actual *p*-value after correcting for multiple testing using the FDR method (black>0.05 and pink<0.05). The second and third values are effect size calculated as the nonoverlapping percentage of the two distributions and Cohen's *d* using the Hedges' *g* estimator, respectively.

The GC contents of the homogeneous and nonhomogeneous domains in eutherians exhibit a non-normal distribution (Lilliefors goodness-of-fit test, *p*<0.05) with a mean of 42–44% and a standard deviation of 5.7–8.5%. The GC distributions of compositional domains of the same type are significantly different from one another, particularly between related taxa (Kolmogorov-Smirnov goodness-of-fit test, *p*<0.01); however, this is due to the large sample sizes. Similar to the patterns observed in compositional domain lengths, the small differences in the GC contents of homogeneous and nonhomogeneous domains allow grouping the species into five taxonomic groups: Primates, Laurasiatheria, Muridae, opossum, and chicken (Figure 7). Of these groups, only the Primate and Laurasiatheria exhibit a high degree of similarity in compositional domain length distribution. Murids and opossum have the most variable GC distribution (38% area nonoverlap) (Figure 7).

With the exception of the murid genomes (*γ*≈0.29), the low frequency of short GC-poor domains and the abundance of medium GC-rich domains causes mammalian GC distributions to be highly right-skewed (0.56<*γ*<0.77) (Figure 7, Table S2). Opossum (*γ*≈1.12) and chicken (*γ*≈0.86) are the most right-skewed of all species, due to the high abundance of short GC-rich and medium-short GC-rich domains, respectively.

**Figure 8:**
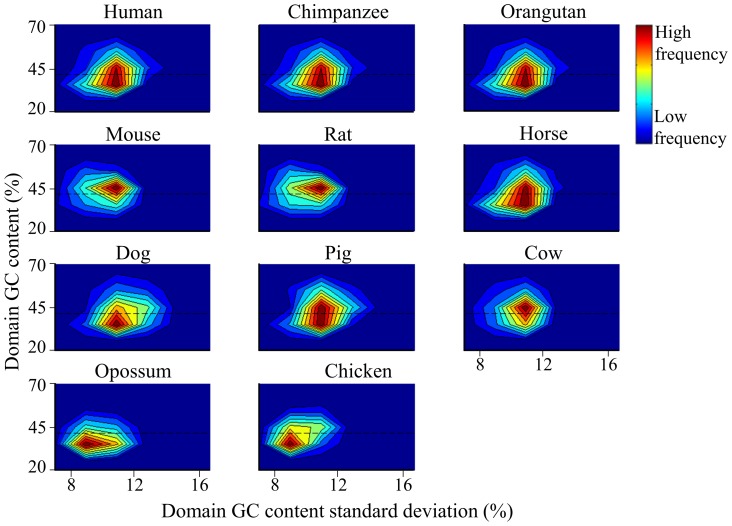
A two dimensional joint distribution of homogeneous domain GC content and its standard deviation (*GCσ*). Each domain GC content and *GCσ* are represented by a point on the map. The frequency of different points is represented by colors ranging from red (highest frequency) to blue (lowest frequency). The mean GC content of the mammalian genome is marked by horizontal line.

To further study the GC content fluctuations within compositional domains, we looked at their compositional variability. Compositional variability is measured from the standard deviation (*GCσ*) of the GC content of each domain calculated over short nonoverlapping windows within the domain (see Materials and Methods). Figure 8 presents two-dimensional joint distribution of homogeneous domain GC content and *GCσ*. Interestingly, the *GCσ* values of most mammalian domains are narrowly distributed around 11% *GCσ* and, with the exception of opossum that exhibits a smaller variation. In other words, GC-rich domains are more erratic in their composition (high *GCσ*) than GC-poor domains (low *GCσ*). The high compositional variability of horse and dog is also reflected in the wide range of *GCσ* values compared with those of the Cetartiodactyla species.

The opossum is exceptional in exhibiting a *GCσ* gradient toward smaller *GCσ*. The opossum compositional makeup characterized by its low GC content and narrow *GCσ* distribution appears to be an intermediate between mammals and murids. The narrow *GCσ* distribution in the murid genomes is also confounding. The murid joint distributions are largely symmetric about the x-axis (Figure 8), suggesting that the evolutionary processes that shaped the compositional organization of the genome were symmetrical. Similar trends were obtained for the nonhomogeneous domains (Figure S6).

### The joint distribution of compositional domain GC content and length

**Figure 9:**
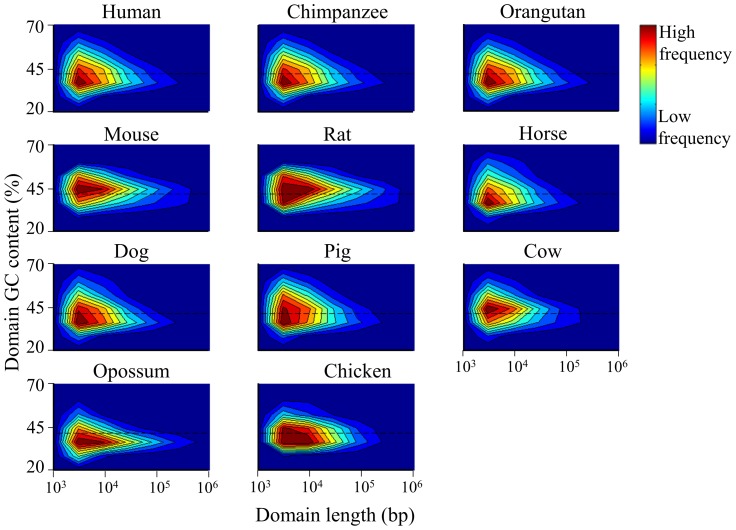
A two dimensional joint distribution of homogeneous domain GC content and length in a log scale. Each domain's GC content and length are represented by a point in the map. The frequency of different points is represented by colors ranging from red (highest frequency) to blue (lowest frequency). The mean GC content of the mammalian genome is marked by horizontal line.

The two-dimensional joint distributions of homogeneous domain GC content and length are shown in Figure 9. These measures are not correlated (*r* = ∼0). As shown before, the majority of domains in all genomes are short (6–8 kb), and their GC content distributes close to the mammalian genome mean GC content. With the exception of murids, homogeneous domains are significantly more AT-rich than nonhomogeneous domains (Kolmogorov-Smirnov goodness-of-fit test, *p*<0.01). The genomic landscape topologies of primates, laurasiatherian, and murids are remarkably similar with short (10^3^–10^4^ bp) GC-rich domains 1.3–1.7 times more frequent than GC-poor domains and medium-large (10^5^–10^6^ bp) GC-rich domains 1–2 times more frequent than GC-poor domains (Table S2). This ratio is opposite for both domain size groups (0.7 and 0.32, respectively) in opossum, which implies a major domain fusion process that affected the tetrapod genome.

Domains in the murid genome have a distinct length distribution compared to other mammals. The murid genome has an abundance of over 2,500 medium-long (10^5^–10^6^ bp) and long (>10^6^ bp) GC-rich domains compared to all other genomes (∼500–1,591) (Table S2). By comparison, in the AT-rich opossum genome, GC-poor domains are twice more frequent than GC-rich domains. The opossum genome is particularly enriched in over 3,500 medium-long and long GC-poor domains compared with only 486 GC-rich domains. Similar results were observed for nonhomogeneous domains (Figure S7).

### Compositional domains and phylogenetic hypotheses

Table 2 summarizes the supporting evidence for the two phylogenetic hypotheses contrasting the validity of Euarchontoglires clade based on the defined genetic attributes. Although the attributes are not independent, qualitatively they provide a strong support for the second hypothesis that places Primates with Laurasiatheria to the exclusion of Muridae, thereby invalidating clade Euarchontoglires (Figure 1).

**Table 2:** A summary of the supporting evidences for the two phylogenetic hypotheses (Figure 1) using seven genetic attributes as selection criteria.

Genomic attributes of compositional domains	Hypothesis I: Muridae clusters with Primates within clade Euarchontoglires	Hypothesis II: Laurasiatheria clusters with Primates to the exclusion of Muridae
Abundance	Murid nonhomogeneous domain counts are closer to those of primates (Table 1).	Murid homogeneous domain counts are closer to those of opossum (Table 1). Murids and opossum have the smallest fraction of homogeneous domains among all mammals (Tables 1, S1).
		Murids and opossum share similarities in mean homogeneous and non homogeneous domain lengths (Table 1).
Length		Murids and chicken exhibit similarity in the distribution of homogeneous domain lengths (Figure 2, Table 1). Short- and medium-length domains (<1 Mb) have similar length distributions in primates, laurasiatherians, and opossum - distinct from murids (Table S2, Figure 3). The murid and opossum genomes have the largest proportion of long homogeneous and nonhomogeneous domains among all species (Table S2).
Density		Domain densities in murid genomes are more similar to those of opossum than to mammals (Figure 4, S2-S3, Table 1).
Genome coverage		The Euclidean distance between the proportion of domains covering the genome show that murids are closer to opossum than to primates (Figure 5).
GC content	The mean GC content of all domain types in murids is similar to that of primates (Figure 7).	
The joint distribution of GC content and length	GC content of short domains exhibit similar topology in murids and primates (Figure 9, Table S2).	GC-rich medium-short domains are more frequenct than GC-poor domains in murids and opossum.

## Discussion

One of the most fascinating features of mammalian genomes is the uniformity of GC content over hundreds and hundreds of thousands base-pairs termed short- and long-range correlations, respectively. Although these structures have been known for over three decades [3], only few explanations were proposed in an evolutionary framework. Most of the explanations for the long-range correlations were related to the “isochore theory.” The “isochore theory” posits the mammalian genome is composed of a mosaic of isochores, long homogeneous domains (typically ≥300 kb) that cover the majority of the genome of “warm-blooded” vertebrates; whereas only a small portion of the genome consists of non-“isochoric” regions. The “cold-blooded” vertebrate genome was described as less compositionally heterogeneous and devoid of GC-rich isochores [5], [12]. Although the theory failed to explain the compositional patterns later found in fish and reptiles [e.g.], [ 43, 62], its importance has been in stimulating follow-up studies that attempted to correlate various biological phenomena with compositional and organizational features. Eventually, following conflicting findings [e.g.], [ 15], [ 36], [ 37], [ 62, 63], ambiguity as to the interpretation of the theory predictions [18] and contradictory revisions of the theory's main principles [e.g., 55](Table S3), the original theory was *de facto* abandoned by most scientists (with the exception of its proponents), leaving open the basic questions: how, when, and why in the course of evolution, did mammalian genomes acquire their current composition and organization?

The most effective approach to understanding the compositional organization of human and mammalian genomes is by comparative analysis – preferably a large-scale one. In a previous analysis of the human genome, Elhaik et al. [15] proposed a compositional domain model to explain its genomic architecture. The compositional domain model portrays the human genome as a mixture of mostly short and very few long homogeneous and nonhomogeneous domains in a ratio of 2∶1. Under this model, “isochoric” domains consist of only a small fraction of all compositional domains [15]. Here, we extended the analysis to ten mammalian genomes and tested whether the outcomes fit within the isochoric or the compositional-domain models using seven genomic attributes. Our findings are discussed under two different phylogenetic hypotheses, for which traditional phylogenetic analyses provided ambiguous answers (Figure 1). Table 2 summarizes the evidence in support of either hypothesis.

The mammalian genome is covered by a complex medley of nonhomogeneous domains of various lengths (32%), short (10^3^–10^4^ bp) homogeneous domains (36%), medium-short (10^4^–10^5^ bp) homogeneous domains (26%), medium-long (10^5^–10^6^ bp) homogeneous domains (4%), and only a miniscule fraction of 0.16% long (10^6^–10^7^ bp) homogeneous domains (Table S2). On average, homogeneous domains longer than 300 kb, i.e., isochores, constitute less than 2% of all domains and cover less than 28% of the mammalian genome (Table 1). Short homogeneous domains have wide GC content distributions and the GC content of long homogeneous domains is distributed slightly below the mammalian genome mean GC content (Figure 9)m whereas the GC content of long nonhomogeneous domains is distributed slightly above it.

Under the “isochore model” where the vast portion of the genome was considered to be composed of long homogeneous domains, their length distribution was thought to display a power-law distribution [18], [49], [53], [64]. We demonstrated that the power-law model is inconsistent with the data due to the high abundance of short domains and the scarcity of long domains (Figures 6, S4, S5). Short domains are major components of the mammalian genome and cannot be dismissed as “false positives“ [55]. Overall, our results support the compositional domain model and limit the applicability of the isochore model to less than 30% of the average mammalian genome.

Homogeneous or “relatively homogeneous” [9] domains were speculated to be biologically different from nonhomogeneous domains [7], [18], [55], yet we found only minor differences between and within chromosomes, most of which stemmed from the differences in the proportions of the two domain types (Tables 1, S2). Interestingly, with the exception of murid genomes, we found that homogeneous domains are significantly more AT-rich than nonhomogeneous domains (Figures 9, S7), which may suggest biological importance. To support such hypothesis, additional biological properties should be used to test whether or not this distinction is biologically meaningful.

Most genome characteristics within higher taxa follow phylogenetic relatedness. For example, the genomes of the three primates are very similar to each other, as are the genomes of the two murids. The genome characteristics of the Pegasoferae (horse and dog) differ slightly from those of cetartiodactyls (cow and pig), possibly adding support for the validity of clade Pegasoferae (Figure 1). However, the possibility that the similarity between horse and dog is due to the poor quality of their genomic sequences cannot be excluded. We have evidence obtained by comparing a draft of the cow genome (build 3.1) with the finished version (build 4.0) [1] that draft genomes contain an abundance (∼90%) of short compositional domains (<10 kb), thus rendering drafts genomes artificially similar to one another.

Overall, the laurasiatherian genomes are more similar to the primate genomes than the murid genomes, which, in turn, are more similar to the opossum genome than to any other genome (Table 2). The murid genome is distinguished from the primate and laurasiatherian genomes mainly by its narrow GC content distribution (Figure 7), larger GC-rich domains (Figures 2, 3), smaller GC content standard deviation for both GC-poor and -rich domains (Figure 8), and the unique shape of its joint distribution of compositional domain GC content and length (Figure 9). Differences in the compositional patterns between murids and other mammals were previously termed the “murid pattern” [65] or “murid shift” [66]. The “shift” was attributed to a smaller variation in the composition of isochoric domains compared to other mammals [66]; however, we found that the differences between the murid lineage to other mammals are found in the entire murid genomes and are not unique to “isochoric” domains. A possible explanation to the “shift” may be in the different evolutionary origin of murids (Figure 1b). Moreover, the similarity between the murid and opossum genomes suggests the effect was not unique to murids and may have originated in the eutherian ancestor.

The two phylogenetic hypotheses tested here differ in the validity of clade Euarchontoglires. According to the first hypothesis (Figure 1a), murids arose relatively late in mammalian evolution and are grouped with Primates under Euarchontoglires. Considering the relatively fast mutation rate of the murids [67], the most parsimonious explanation would be that their genomic organization is a derived state, possibly as a result of a “shift” or a genomic transition that affected the entire linage. Under this hypothesis, the genomic transition resulted in the fusion of nearly half of the short domains of extreme GC content together with other domains. Elongated domains were created due to the decrease in GC content variability and the fusion of neighboring domains. Subsequently, domain density was reduced and the compositional fluctuations were “flattened” resulting in higher homogeneity between domains. The process dramatically decreased the proportion of short domains (52%) that are highly frequent in other mammalian genomes (60%). Conversely, these fusions increased the proportion of longer domains (medium-short = 40%, medium-long = 7.5%, long = 0.28%) compared to all other mammalian domains (medium-short = 36%, medium-long = 4%, long = 0.15%). The proportion of long GC-poor domains increased as well but in smaller proportion than GC-rich domains. Further evidence for this transition can be found in the frequency distribution of GC content standard deviation that is relatively devoid of heterogeneous domains compared to other mammalian genomes (Figure 8). Moreover, Muridae have genomes that are markedly homogeneous in both poor- and GC-rich domains, as opposed to mammalians genomes that are highly heterogeneous in their GC-rich domains and homogeneous in their GC-poor domains (Table S2). We note that genome elongation could also result from segmental duplication; however, we do not know of a segmental duplication that acts selectively on segments with certain composition.

According to the second hypothesis (Figure 1b), murids arose early in the mammalian evolution and their genomic architecture reflects an ancestral state. The “typical” mammalian genome thus evolved from this ancestral pattern leading to a wider compositional distribution and shorter domains. This view is supported by the similar genomic structure (Tables 1, S2) and genome homogeneity shared between the murid and opossum genomes. A similar hypothesis was tested by Mouchiroud, Gautier, and Bernardi [68]; however, because they assumed the existence of isochores that cover the mammalian genome, their conclusions are limited to few “isochoric” domains.

Unfortunately, the representation of marsupial mammal as outgroup yielded more questions than answers as opossum reflected either unique genomic characteristics or oscillated between murid and non-murid characteristics (Tables 1–2). Thus, although the results showed a high resemblance between murids and opossum in support of the second hypothesis (Table 2), additional evidence would be necessary before ruling out the first hypothesis (Figure 1). It is possible that with the accumulation of additional genomic sequences of intermediate species this question would be answered. In light of these findings, it will be intriguing to identify which evolutionary mechanisms shaped the transitions that affected the murid and opossum genomes. Understanding these biological mechanisms and their evolutionary implications is a key factor in reconstructing the evolutionary history of mammalian genome evolution.

## Materials and Methods

### Data

Nine eutherian genomes that are either fully sequenced or have reliable genomic drafts were included in this study: human (*Homo sapiens* build 37.1), chimpanzee (*Pan troglodytes* build 2.1), orangutan (*Pongo abelii* build 1.2), mouse (*Mus musculus* build 37.1), rat (*Rattus norvegicus* build 4.1), horse (*Equus caballus* build 2.1), dog (*Canis familiaris* build 2.1), pig (*Sus scrofa* build 2.1), and cow (*Bos taurus* build 4.1). The gray short-tailed opossum (*Monodelphis domestica* build 2.1) was used as an outgroup to the eutherians, and chicken (*Gallus gallus* build 2.1) was used as an outgroup to the mammals. Genomes were downloaded from http://www.ncbi.nlm.nih.gov/Genomes/. Nulls, i.e., unknown, undetermined, or ambiguous characters in the genomic sequences, were discarded.

### Phylogenetic hypotheses

There are two phylogenetic hypotheses in the literature for the taxa under study (Figure 1). The two hypotheses are supported by molecular data though differ in their outcome. The difference between the two phylogenetic trees concerns the relative kinship of murids (mouse and rat) and laurasiatherians (horse, dog, cow, and pig) to primates (human, chimpanzee, and orangutan). In the first scheme [e.g.], [ 29], [ 69], [ 70]–[72], primates cluster with the murids within clade Euarchontoglires (Figure 1a). In the second scheme [e.g.], [ 30, 73], primates cluster with the laurasiatherians to the exclusion of murids (Figure 1b). The clustering of Perissodactyla (horse) and Carnivora (dog) into Pegasoferae to the exclusion of Cetartiodactyla (cow and pig) is accepted by both alternative phylogenies [69].

### Genome segmentation into compositional domains

Version 2 of IsoPlotter [15] of the IsoPlotter+ pipeline [28] was obtained from https://github.com/sean-dougherty/isoplotter/and used to partition each of the genomes into compositionally distinct domains. IsoPlotter recursively maximizes the difference in GC content between adjacent segments, as measured by the Jensen-Shannon divergence statistic [17]. The halting criterion was obtained via a dynamic threshold calculated in real-time according to the length of each segment and the standard deviation of its GC content. The compositional domains inferred by the segmentation procedure were classified into homogeneous and nonhomogeneous as in Elhaik et al. [15]. For convenience, domains are sometimes divided by order of magnitude of their length into: short (10^3^–10^4^ bp), medium-short (10^4^–10^5^ bp), medium-long (10^5^–10^6^ bp), and long (10^6^–10^7^ bp) domains.

The mean GC content of all mammalian genomes in this study (40.9%) was used as a critical value. A compositional domain was defined as GC-rich or GC-poor if its GC content was higher or lower, respectively, than the critical value.

### Comparisons of the distributions of domain length and domain composition

For each species and for each domain category, log domain-lengths were sorted and smoothed. Smoothing was carried by dividing the log domain-lengths into 1,000 groups of equal size and then using the mean domain length of each group to calculate a histogram with 38 bins ranging from 8 to 16. To test whether or not two distributions are different, we used the Kolmogorov-Smirnov goodness-of-fit test and the False Discovery Rate (FDR) correction for multiple tests [74]. Because the differences between the distributions were highly significant due to the huge sample sizes, we also calculated the effect size, first by using the nonoverlapping percentage of the two distributions, and then by using Hedges' *g* estimator of Cohen's *d* [75]. If the area overlap was larger than 98% and Cohen's *d* was smaller than 0.05, we considered the magnitude of the difference between the two distributions to be too small to be biologically meaningful.

The distributions of domain GC contents were calculated in a similar manner. To smooth the GC content distributions, domain GC contents were divided into 1,000 groups of equal size, and the mean domain GC content of each group was used to calculate a histogram with 38 bins ranging from 0 to 1. The remaining calculations were carried as described above.

To test whether the GC-content distributions of homogeneous and nonhomogeneous domains fit a normal distribution, we used the Lilliefors (1967) test. This test is a two-sided goodness-of-fit test suitable when a fully-specified null distribution is unknown and its parameters must be estimated. It tests the null hypothesis that domain GC contents come from a distribution in the normal family, against the alternative that they do not come from a normal distribution.

We also estimated the standardized skewness (*γ*) of the GC content distributions using the “skewness” function in Matlab, which first centralizes the distribution by subtracting it from its mean, calculates its third (*k_3_*) and second (*k_2_*) moments, and then computes the skewness, so that: *GC_0_* = *GC* – *μ*(*GC*), *k_3_* = *μ*(*GC_0_* ^3^), *k_2_* = *μ*(*GC_0_* ^2^), and *γ* = *k_3_*/*k_2_* ^1.5^.

### Fit to power-law distribution

We used two approaches to test the fit of the domain-length distributions to power-laws. First, the minimum domain length and the power-law exponent were estimated for the domain lengths of each genome according to the goodness-of-fit based method described in Clauset, Shalizi, and Newman [51]. The observed domain lengths were then compared to the domain lengths generated from the parameters previously estimated, and the similarity between the two distributions was calculated using the Kolmogorov-Smirnov statistic [76]. Based on the observed goodness-of-fit, we calculated a *p*-value that quantifies the probability that the data were drawn from the hypothesized distribution. We used the Matlab scripts plfit.m (version 1.0.5), plpva.m (version 1.0.6), and plplot.m (version 1.0) in www.santafe.edu/~aaronc/powerlaws/(Clauset, Shalizi, and Newman [51]. Second, Baek and et al. [52] showed that the random group formation (RGF) model is a form of general distribution, free from system-specific assumptions, of which pure power-laws are a special case. We used this model to test the data fitting into the power-law model using the online application http://www.tp.umu.se/~garuda/Comp.html.

## Supporting Information

Figure S1 **The cumulative distribution of medium-short (10^4^–10^5^) and medium-long (10^5^–10^6^) nonhomogeneous (a) and compositional (b) domain sizes in log scale.** For simplicity, the mean distributions of primates, murids, and laurasiatherians are shown.
(TIF)

Figure S2 **Compositional domain densities of A) homogeneous and B) nonhomogeneous domains over all chromosomes.** Box plots summarize medians, quartiles, and range.
(TIF)

Figure S3 **Frequency of domain density for (a) homogeneous domains, (b) nonhomogeneous domains, and (c) compositional domains.** GC-poor domains (red), GC-rich domains (blue), and all domains (black) are plotted.
(TIFF)

Figure S4 **The cumulative density function** ***P*** **(x) of compositional domain (a) and nonhomogeneous domain (b) sizes (x) (points) plotted on a log-log scale.** The solid lines represent the maximum likelihood power-law fits to the data.
(TIF)

Figure S5 **A comparison of compositional (a), homogeneous (b), and nonhomogeneous (c) domain lengths in the cumulative representation** ***C*** **(** ***k*** **).** The horizontal axis is plotted as *k*/*k*0, where *k*0, is the size of the smallest domain size group. Although the distributions are clearly different for all the animals, the deviation from a power-law (dashed line) and the “fat tails” are shared features.
(TIFF)

Figure S6 **A two dimensional joint distribution of nonhomogeneous domain GC content and its standard deviation (GCσ).** Each domain GC content and GCσ are represented by a point on the map. The frequency of different points is represented by colors ranging from red (highest frequency) to blue (lowest frequency). The mean GC content of the mammalian genome is marked by horizontal line.
(TIF)

Figure S7 **A two dimensional joint distribution of nonhomogeneous domain GC content and size in a log scale.** Each domain GC content and its size are represented by a point in the map. The frequency of different points is represented by colors ranging from red (highest frequency) to blue (lowest frequency).
(TIF)

Table S1 **Chromosome statistics for compositionally homogeneous, nonhomogeneous, and “isochoric” domains.**
(DOC)

Table S2 **Categories of compositional domains by length and GC content.**
(DOC)

Table S3 List of 49 publications by Professor Giorgio Bernardi and colleagues in which isochores are defined as compositionally homogeneous genomic stretches longer than 300 kb*#.
(DOC)
