# Supplementary figures and images for: A Comparative Study and a Phylogenetic Exploration of the Compositional Architectures of Mammalian Nuclear Genomes
Source: PLoS Comput Biol. 2014 Nov 6;10(11):e1003925. doi: 10.1371/journal.pcbi.1003925 (PMC4222635; doi:10.1371/journal.pcbi.1003925)

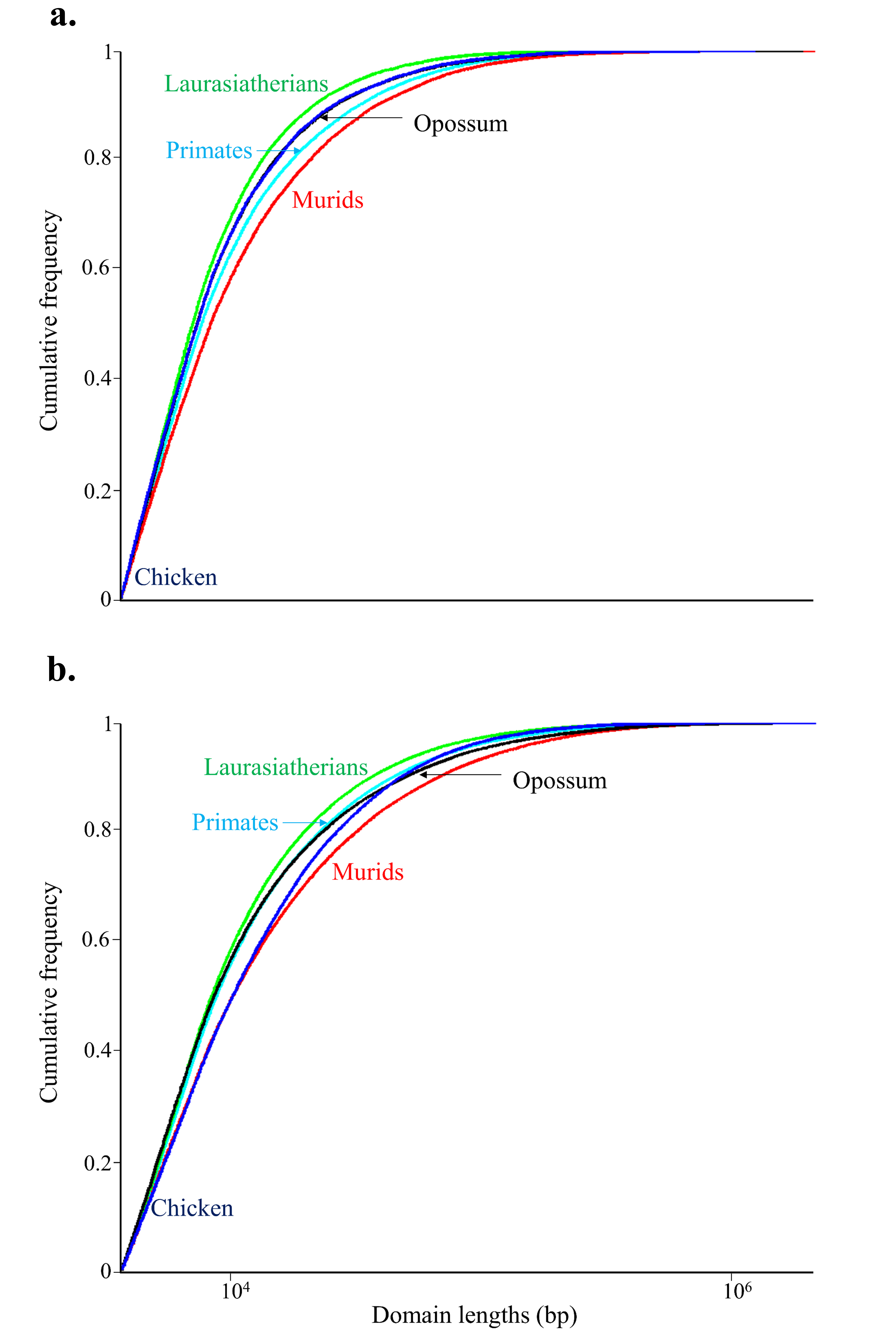

Supplement: Figure S1 — The cumulative distribution of medium-short (104–105) and medium-long (105–106) nonhomogeneous (a) and compositional (b) domain sizes in log scale. For simplicity, the mean distributions of primates, murids, and laurasiatherians are shown. (TIF) [file pcbi.1003925.s001.tif]

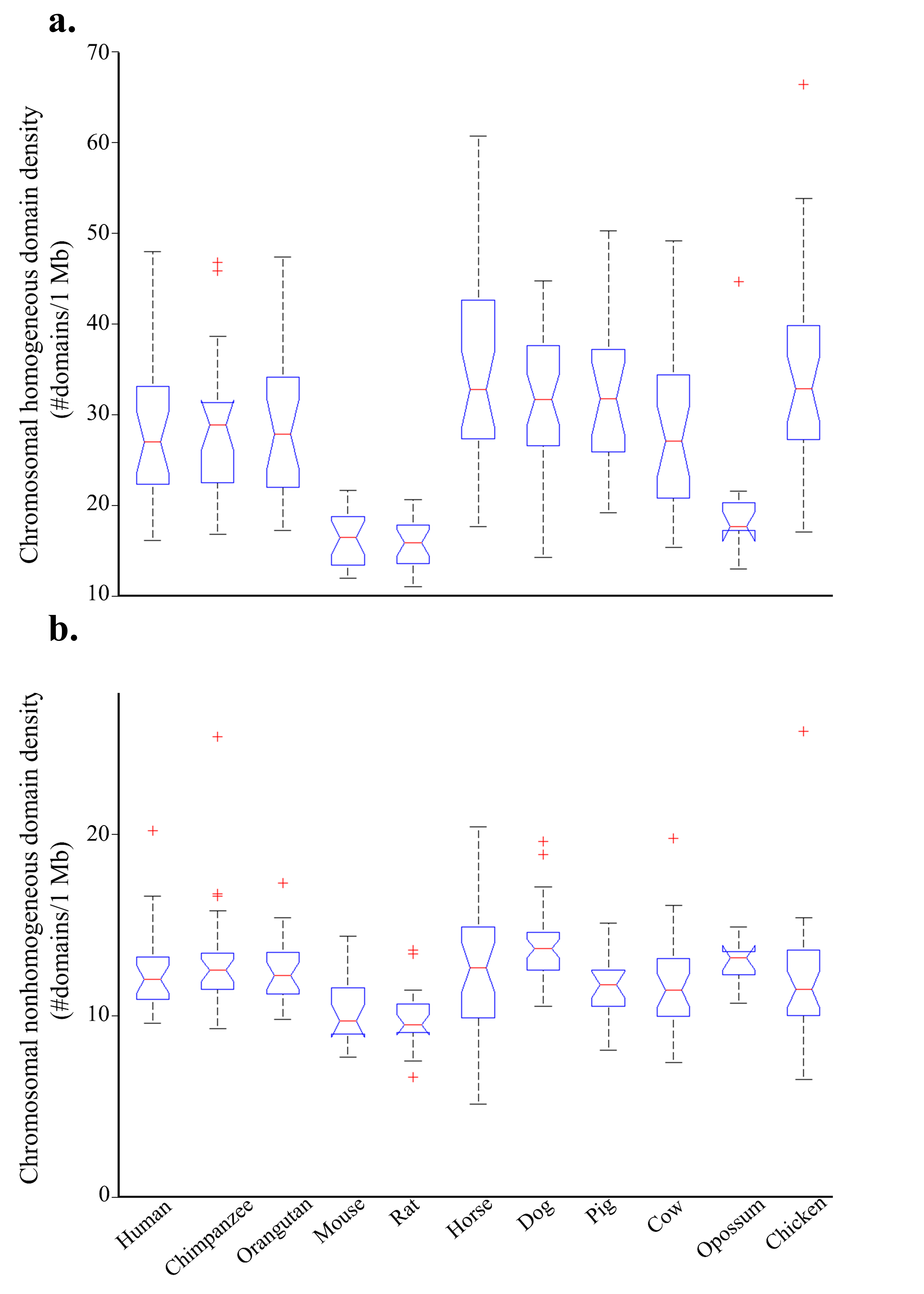

Supplement: Figure S2 — Compositional domain densities of A) homogeneous and B) nonhomogeneous domains over all chromosomes. Box plots summarize medians, quartiles, and range. (TIF) [file pcbi.1003925.s002.tif]

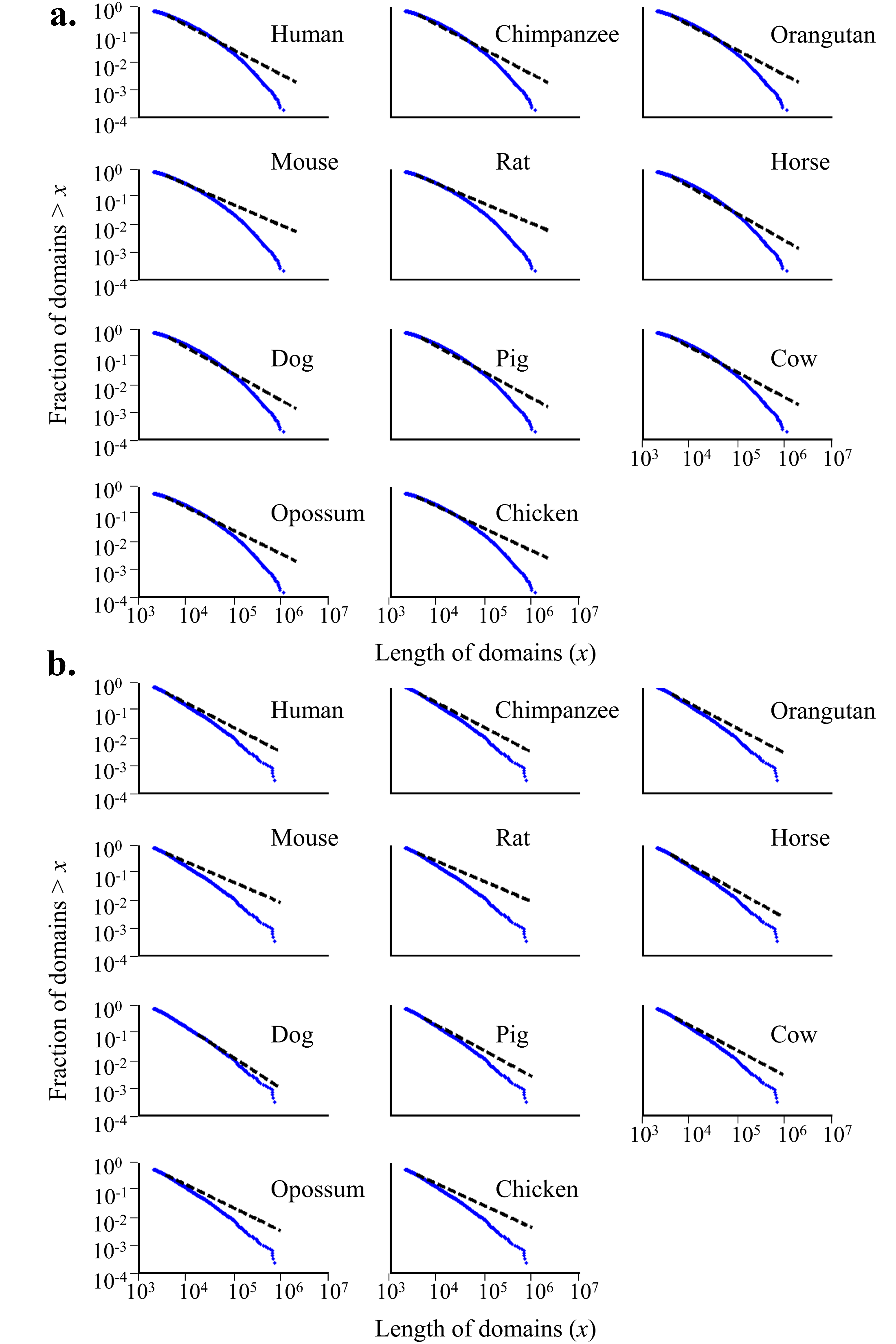

Supplement: Figure S4 — The cumulative density function P (x) of compositional domain (a) and nonhomogeneous domain (b) sizes (x) (points) plotted on a log-log scale. The solid lines represent the maximum likelihood power-law fits to the data. (TIF) [file pcbi.1003925.s004.tif]

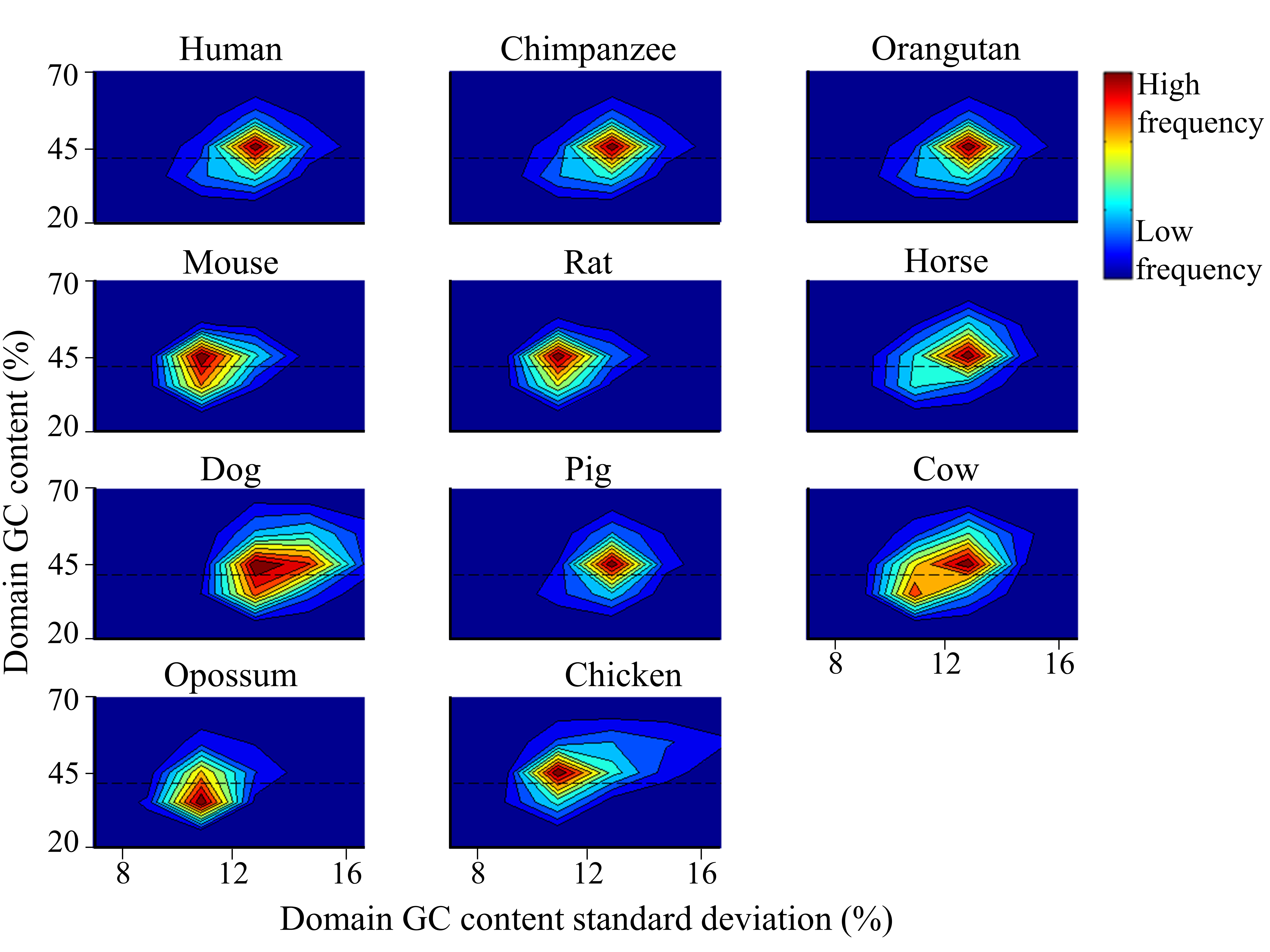

Supplement: Figure S6 — A two dimensional joint distribution of nonhomogeneous domain GC content and its standard deviation (GCσ). Each domain GC content and GCσ are represented by a point on the map. The frequency of different points is represented by colors ranging from red (highest frequency) to blue (lowest frequency). The mean GC content of the mammalian genome is marked by horizontal line. (TIF) [file pcbi.1003925.s006.tif]

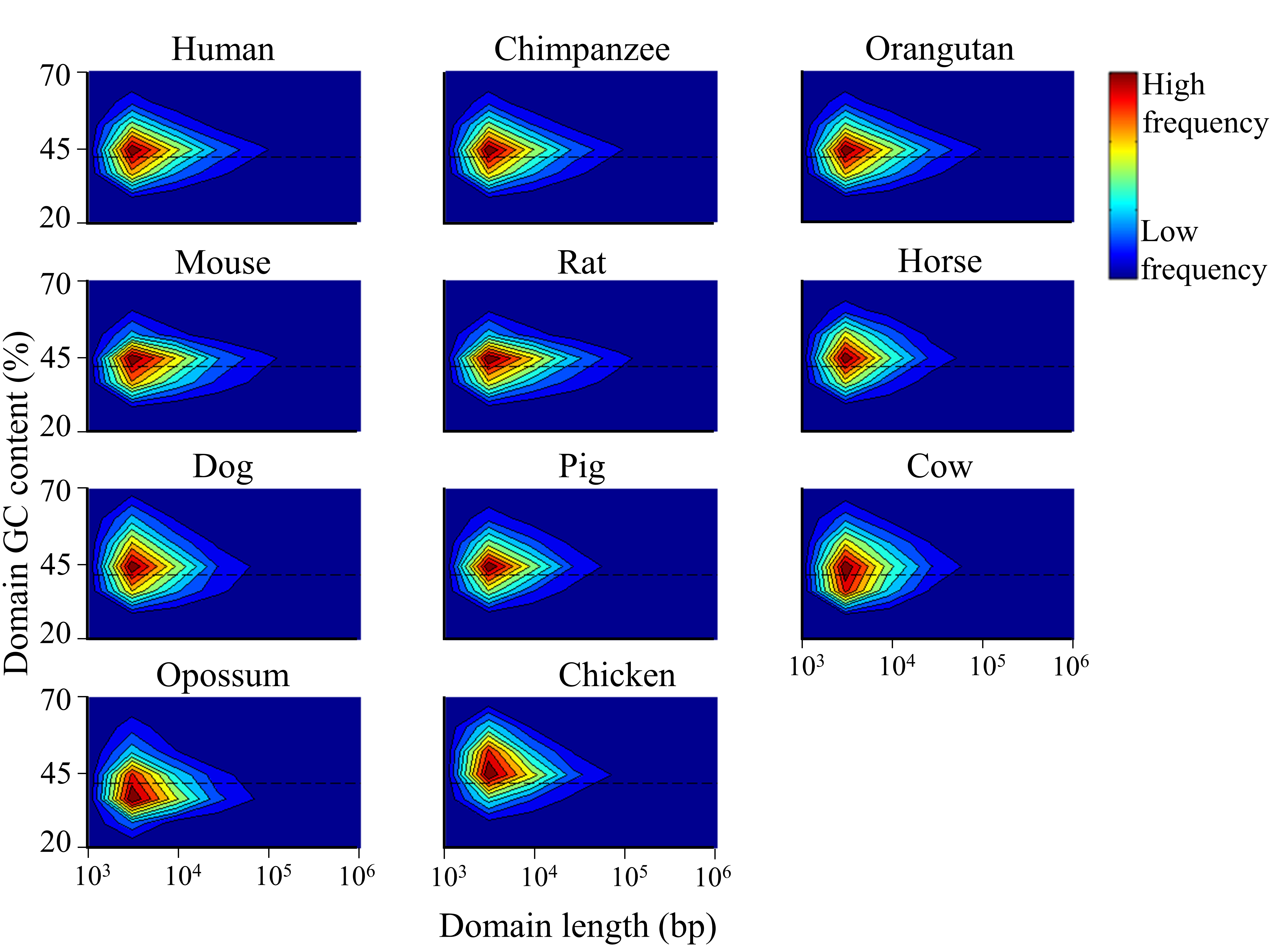

Supplement: Figure S7 — A two dimensional joint distribution of nonhomogeneous domain GC content and size in a log scale. Each domain GC content and its size are represented by a point in the map. The frequency of different points is represented by colors ranging from red (highest frequency) to blue (lowest frequency). (TIF) [file pcbi.1003925.s007.tif]
