# Supplementary material for: A Comparative Study and a Phylogenetic Exploration of the Compositional Architectures of Mammalian Nuclear Genomes
Source: PLoS Comput Biol. 2014 Nov 6;10(11):e1003925. doi: 10.1371/journal.pcbi.1003925 (PMC4222635; doi:10.1371/journal.pcbi.1003925)
Supplement: Table S1 — Chromosome statistics for compositionally homogeneous, nonhomogeneous, and “isochoric” domains. (DOC) [file pcbi.1003925.s008.doc]

**Table S1. Chromosome statistics for compositionally homogeneous, nonhomogeneous, and “isochoric” domains**

(a) Compositional domains

| **Species** | **Chr** | **Chr size** | **Density (per Mb)** | **Mean size (bp)** | **Median size (bp)** | **Mean GC%** | **Median GC%** | **#Domains** | **Genome coverage (%)** | **Longes**t **domain** |
| --- | --- | --- | --- | --- | --- | --- | --- | --- | --- | --- |
| Human | 1 | 217,916,736 | 40.6 | 24,601 | 7,936 | 43.1 | 43 | 8,858 | 100 | 2,875,136 |
| Human | 2 | 233,385,152 | 39.6 | 25,269 | 7,648 | 41.8 | 41 | 9,236 | 100 | 2,295,040 |
| Human | 3 | 194,309,312 | 32.5 | 30,804 | 8,352 | 41.2 | 40 | 6,308 | 100 | 3,243,616 |
| Human | 4 | 185,864,768 | 25.8 | 38,795 | 8,128 | 40 | 39 | 4,791 | 100 | 3,790,592 |
| Human | 5 | 174,600,768 | 35.4 | 28,216 | 7,920 | 41 | 40 | 6,188 | 100 | 3,259,072 |
| Human | 6 | 167,628,544 | 33.2 | 30,106 | 8,064 | 40.8 | 40 | 5,568 | 100 | 2,701,312 |
| Human | 7 | 151,582,880 | 37.6 | 26,598 | 7,712 | 42.7 | 42 | 5,699 | 100 | 2,859,264 |
| Human | 8 | 140,746,368 | 36.8 | 27,182 | 7,584 | 41.9 | 41 | 5,178 | 100 | 2,484,160 |
| Human | 9 | 109,434,656 | 45 | 22,211 | 7,872 | 43.3 | 42 | 4,927 | 100 | 1,134,400 |
| Human | 10 | 128,196,704 | 46.6 | 21,481 | 7,488 | 42.8 | 42 | 5,968 | 100 | 2,046,944 |
| Human | 11 | 130,274,560 | 43.8 | 22,807 | 7,744 | 43.7 | 43 | 5,712 | 100 | 1,807,808 |
| Human | 12 | 129,843,424 | 34.6 | 28,931 | 8,128 | 42.3 | 42 | 4,488 | 100 | 2,730,848 |
| Human | 13 | 94,957,600 | 30.6 | 32,733 | 8,032 | 39.7 | 39 | 2,901 | 100 | 4,510,208 |
| Human | 14 | 86,964,576 | 37.4 | 26,734 | 8,096 | 42.9 | 42 | 3,253 | 100 | 1,328,128 |
| Human | 15 | 77,770,144 | 49.3 | 20,274 | 7,712 | 43 | 42 | 3,836 | 100 | 1,032,608 |
| Human | 16 | 74,960,800 | 45.4 | 22,008 | 7,904 | 45.9 | 45 | 3,406 | 100 | 1,182,560 |
| Human | 17 | 75,513,088 | 51.1 | 19,553 | 7,840 | 46.9 | 47 | 3,862 | 100 | 1,347,680 |
| Human | 18 | 74,291,328 | 35 | 28,563 | 7,808 | 40.7 | 40 | 2,601 | 100 | 1,339,392 |
| Human | 19 | 54,533,856 | 61.8 | 16,187 | 7,680 | 48.8 | 49 | 3,369 | 100 | 686,112 |
| Human | 20 | 59,266,464 | 45.1 | 22,164 | 7,504 | 45.2 | 45 | 2,674 | 100 | 5,243,904 |
| Human | 21 | 33,211,648 | 44 | 22,748 | 7,168 | 43.2 | 42 | 1,460 | 100 | 842,688 |
| Human | 22 | 33,725,632 | 64.6 | 15,485 | 7,200 | 48.5 | 49 | 2,178 | 100 | 487,808 |
| Human | X | 135,172,992 | 31.7 | 31,502 | 7,968 | 41 | 40 | 4,291 | 100 | 4,823,840 |
| Human | Y | 18,186,240 | 45 | 22,205 | 7,744 | 39.8 | 39 | 819 | 100 | 674,656 |
| Chimpanzee | 1 | 217,189,792 | 42.5 | 23,544 | 7,968 | 43.1 | 43 | 9,225 | 100 | 2,890,400 |
| Chimpanzee | 2A | 105,873,440 | 44.2 | 22,627 | 7,456 | 42.5 | 42 | 4,679 | 100 | 2,945,760 |
| Chimpanzee | 2B | 127,869,088 | 36.7 | 27,282 | 7,872 | 41.5 | 40 | 4,687 | 100 | 1,896,704 |
| Chimpanzee | 3 | 194,962,656 | 34.1 | 29,340 | 8,352 | 41.2 | 40 | 6,645 | 100 | 2,527,744 |
| Chimpanzee | 4 | 186,955,680 | 27.7 | 36,043 | 8,288 | 39.9 | 39 | 5,187 | 100 | 6,451,104 |
| Chimpanzee | 5 | 175,225,344 | 35.3 | 28,294 | 7,968 | 41.2 | 40 | 6,193 | 100 | 4,092,448 |
| Chimpanzee | 6 | 164,697,664 | 32 | 31,222 | 8,128 | 40.8 | 40 | 5,275 | 100 | 3,734,208 |
| Chimpanzee | 7 | 151,069,600 | 38.8 | 25,749 | 7,776 | 42.3 | 41 | 5,867 | 100 | 3,055,232 |
| Chimpanzee | 8 | 138,150,976 | 38.5 | 25,954 | 7,776 | 41.5 | 41 | 5,323 | 100 | 2,512,032 |
| Chimpanzee | 9 | 109,295,936 | 42.5 | 23,530 | 7,744 | 43.2 | 42 | 4,645 | 100 | 2,176,352 |
| Chimpanzee | 10 | 125,696,576 | 44.4 | 22,522 | 7,616 | 42.6 | 42 | 5,581 | 100 | 2,019,232 |
| Chimpanzee | 11 | 123,596,640 | 45.5 | 21,973 | 7,584 | 44 | 43 | 5,625 | 100 | 2,789,280 |
| Chimpanzee | 12 | 129,867,936 | 35.7 | 28,001 | 8,224 | 42.3 | 42 | 4,638 | 100 | 2,485,024 |
| Chimpanzee | 13 | 87,794,784 | 29.6 | 33,819 | 7,600 | 40 | 39 | 2,596 | 100 | 2,574,464 |
| Chimpanzee | 14 | 86,251,392 | 39.1 | 25,556 | 7,776 | 43.1 | 42 | 3,375 | 100 | 2,124,480 |
| Chimpanzee | 15 | 76,971,936 | 47.3 | 21,135 | 7,616 | 43.2 | 43 | 3,642 | 100 | 2,496,064 |
| Chimpanzee | 16 | 74,505,664 | 46.8 | 21,379 | 8,000 | 45.7 | 45 | 3,485 | 100 | 1,239,136 |
| Chimpanzee | 17 | 73,429,760 | 51.5 | 19,400 | 7,808 | 47 | 47 | 3,785 | 100 | 1,173,408 |
| Chimpanzee | 18 | 74,181,056 | 34.8 | 28,741 | 7,744 | 40.9 | 40 | 2,581 | 100 | 1,574,752 |
| Chimpanzee | 19 | 51,997,056 | 61.7 | 16,198 | 7,616 | 48.6 | 49 | 3,210 | 100 | 967,360 |
| Chimpanzee | 20 | 58,102,080 | 44.5 | 22,459 | 7,648 | 45 | 45 | 2,587 | 100 | 5,290,400 |
| Chimpanzee | 21 | 32,706,016 | 40 | 24,985 | 7,136 | 43.5 | 42 | 1,309 | 100 | 1,040,576 |
| Chimpanzee | 22 | 32,341,152 | 63.5 | 15,753 | 7,136 | 48.3 | 48 | 2,053 | 100 | 611,072 |
| Chimpanzee | X | 130,929,824 | 29.9 | 33,469 | 8,224 | 40.8 | 40 | 3,912 | 100 | 2,963,680 |
| Chimpanzee | Y | 22,691,168 | 55.3 | 18,095 | 7,616 | 40.9 | 40 | 1,254 | 100 | 544,128 |
| Orangutan | 1 | 216,060,736 | 43.7 | 22,861 | 7,968 | 43.1 | 42 | 9,451 | 100 | 3,571,680 |
| Orangutan | 2A | 104,616,320 | 43.1 | 23,227 | 7,776 | 42.3 | 42 | 4,504 | 100 | 1,534,944 |
| Orangutan | 2B | 148,146,336 | 30.7 | 32,610 | 8,256 | 40.9 | 40 | 4,543 | 100 | 2,110,048 |
| Orangutan | 3 | 190,355,264 | 31.6 | 31,663 | 8,448 | 41.1 | 40 | 6,012 | 100 | 2,245,184 |
| Orangutan | 4 | 186,135,648 | 28.6 | 34,955 | 8,224 | 39.8 | 39 | 5,325 | 100 | 4,315,296 |
| Orangutan | 5 | 172,726,976 | 33.2 | 30,155 | 8,032 | 40.8 | 40 | 5,728 | 100 | 3,532,000 |
| Orangutan | 6 | 164,088,672 | 29.1 | 34,371 | 8,496 | 40.8 | 40 | 4,774 | 100 | 2,809,664 |
| Orangutan | 7 | 145,686,624 | 40.2 | 24,865 | 7,776 | 42.3 | 41 | 5,859 | 100 | 2,257,312 |
| Orangutan | 8 | 140,594,368 | 37 | 27,037 | 7,968 | 41.7 | 41 | 5,200 | 100 | 2,616,224 |
| Orangutan | 9 | 109,204,832 | 43.4 | 23,063 | 7,776 | 43.2 | 42 | 4,735 | 100 | 2,453,120 |
| Orangutan | 10 | 124,371,648 | 44.1 | 22,662 | 7,648 | 42.8 | 42 | 5,488 | 100 | 2,168,032 |
| Orangutan | 11 | 124,382,944 | 45.8 | 21,837 | 7,632 | 43.5 | 43 | 5,696 | 100 | 1,835,904 |
| Orangutan | 12 | 128,777,280 | 37.9 | 26,389 | 8,000 | 42 | 41 | 4,880 | 100 | 2,170,048 |
| Orangutan | 13 | 94,686,048 | 27.4 | 36,488 | 8,288 | 39.9 | 39 | 2,595 | 100 | 3,623,136 |
| Orangutan | 14 | 86,577,920 | 37.8 | 26,476 | 8,032 | 42.5 | 41 | 3,270 | 100 | 1,752,128 |
| Orangutan | 15 | 75,961,056 | 51.1 | 19,588 | 7,904 | 42.8 | 42 | 3,878 | 100 | 983,680 |
| Orangutan | 16 | 70,802,048 | 47.2 | 21,173 | 7,984 | 45.7 | 45 | 3,344 | 100 | 1,024,512 |
| Orangutan | 17 | 67,081,376 | 53.3 | 18,764 | 7,936 | 47 | 47 | 3,575 | 100 | 2,953,984 |
| Orangutan | 18 | 73,515,712 | 36.3 | 27,575 | 8,032 | 40.7 | 40 | 2,666 | 100 | 1,482,720 |
| Orangutan | 19 | 51,367,232 | 60 | 16,672 | 7,488 | 48.4 | 48 | 3,081 | 100 | 687,712 |
| Orangutan | 20 | 58,061,408 | 52.7 | 18,974 | 7,552 | 44.8 | 44 | 3,060 | 100 | 1,316,800 |
| Orangutan | 21 | 33,052,576 | 38.7 | 25,822 | 7,136 | 43.4 | 43 | 1,280 | 100 | 2,045,216 |
| Orangutan | 22 | 30,217,376 | 64.6 | 15,472 | 7,264 | 48.4 | 49 | 1,953 | 100 | 515,936 |
| Orangutan | X | 126,496,928 | 37.9 | 26,403 | 7,904 | 41.3 | 40 | 4,791 | 100 | 1,796,768 |
| Mouse | 1 | 191,477,376 | 22.3 | 44,927 | 9,264 | 42.6 | 42 | 4,262 | 100 | 5,166,272 |
| Mouse | 2 | 178,392,032 | 28.2 | 35,430 | 9,664 | 43.3 | 43 | 5,035 | 100 | 2,878,336 |
| Mouse | 3 | 156,393,856 | 22.5 | 44,367 | 9,440 | 41.2 | 41 | 3,525 | 100 | 3,088,992 |
| Mouse | 4 | 151,886,784 | 29.1 | 34,340 | 9,184 | 43.6 | 44 | 4,423 | 100 | 2,657,824 |
| Mouse | 5 | 147,721,152 | 24.2 | 41,355 | 9,568 | 44.1 | 44 | 3,572 | 100 | 3,583,136 |
| Mouse | 6 | 146,316,992 | 23.1 | 43,225 | 9,696 | 42.6 | 42 | 3,385 | 100 | 4,604,448 |
| Mouse | 7 | 141,878,176 | 34 | 29,441 | 8,832 | 44.3 | 44 | 4,819 | 100 | 3,184,416 |
| Mouse | 8 | 124,796,736 | 24.3 | 41,119 | 9,440 | 43.2 | 43 | 3,035 | 100 | 2,590,048 |
| Mouse | 9 | 120,720,160 | 30.8 | 32,452 | 8,896 | 43.9 | 44 | 3,720 | 100 | 1,519,232 |
| Mouse | 10 | 126,847,808 | 23.5 | 42,481 | 9,456 | 42.1 | 42 | 2,986 | 100 | 4,642,368 |
| Mouse | 11 | 118,743,520 | 32.4 | 30,883 | 9,504 | 45.3 | 45 | 3,845 | 100 | 2,125,216 |
| Mouse | 12 | 117,459,264 | 27.5 | 36,298 | 9,120 | 43.1 | 43 | 3,236 | 100 | 3,654,432 |
| Mouse | 13 | 116,370,848 | 24.8 | 40,309 | 9,184 | 42.4 | 42 | 2,887 | 100 | 2,748,672 |
| Mouse | 14 | 121,635,264 | 23.4 | 42,664 | 9,120 | 42.4 | 42 | 2,851 | 100 | 7,258,464 |
| Mouse | 15 | 100,439,936 | 28.5 | 35,107 | 8,640 | 43.7 | 44 | 2,861 | 100 | 2,246,272 |
| Mouse | 16 | 95,004,896 | 25 | 40,036 | 9,664 | 42.1 | 42 | 2,373 | 100 | 1,986,432 |
| Mouse | 17 | 91,898,144 | 28.4 | 35,264 | 8,896 | 43.8 | 44 | 2,606 | 100 | 4,089,664 |
| Mouse | 18 | 87,600,032 | 23 | 43,539 | 9,824 | 42.2 | 42 | 2,012 | 100 | 2,001,440 |
| Mouse | 19 | 58,142,176 | 28 | 35,758 | 9,136 | 44.1 | 44 | 1,626 | 100 | 1,282,016 |
| Mouse | X | 162,080,832 | 25.2 | 39,638 | 8,576 | 40.3 | 40 | 4,089 | 100 | 3,620,864 |
| Mouse | Y | 2,702,496 | 27.8 | 36,033 | 8,864 | 38.2 | 39 | 75 | 100 | 487,904 |
| Rat | 1 | 242,568,192 | 27.9 | 35,788 | 9,088 | 43.7 | 44 | 6,778 | 100 | 4,724,576 |
| Rat | 2 | 235,352,352 | 20.9 | 47,787 | 9,824 | 41.5 | 41 | 4,925 | 100 | 3,260,224 |
| Rat | 3 | 157,225,152 | 28.3 | 35,339 | 9,280 | 44.1 | 44 | 4,449 | 100 | 1,713,856 |
| Rat | 4 | 172,528,416 | 24.4 | 41,000 | 9,120 | 43.1 | 43 | 4,208 | 100 | 2,769,728 |
| Rat | 5 | 157,560,736 | 24.3 | 41,235 | 9,696 | 43.8 | 43 | 3,821 | 100 | 3,627,200 |
| Rat | 6 | 134,552,608 | 25.8 | 38,742 | 9,376 | 43.5 | 43 | 3,473 | 100 | 3,441,056 |
| Rat | 7 | 131,647,424 | 29.4 | 33,982 | 9,056 | 43.8 | 44 | 3,874 | 100 | 4,682,912 |
| Rat | 8 | 118,069,792 | 29.6 | 33,754 | 9,456 | 43.8 | 44 | 3,498 | 100 | 1,555,616 |
| Rat | 9 | 104,712,416 | 22.5 | 44,388 | 9,760 | 43 | 43 | 2,359 | 100 | 2,263,936 |
| Rat | 10 | 101,026,080 | 34.1 | 29,317 | 9,696 | 46.3 | 46 | 3,446 | 100 | 1,567,840 |
| Rat | 11 | 81,764,224 | 21.5 | 46,536 | 9,728 | 42.5 | 42 | 1,757 | 100 | 3,508,672 |
| Rat | 12 | 41,216,160 | 28.4 | 35,258 | 10,016 | 46.8 | 47 | 1,169 | 100 | 1,624,864 |
| Rat | 13 | 102,334,624 | 23.8 | 42,078 | 8,960 | 42.1 | 42 | 2,432 | 100 | 7,583,552 |
| Rat | 14 | 100,801,632 | 25.5 | 39,238 | 9,344 | 42.6 | 42 | 2,569 | 100 | 1,683,296 |
| Rat | 15 | 98,895,328 | 23.2 | 43,167 | 9,632 | 42.5 | 42 | 2,291 | 100 | 3,434,784 |
| Rat | 16 | 81,355,456 | 27.2 | 36,779 | 9,120 | 42.9 | 43 | 2,212 | 100 | 2,278,304 |
| Rat | 17 | 87,674,496 | 22.7 | 43,969 | 9,664 | 43.3 | 43 | 1,994 | 100 | 4,717,408 |
| Rat | 18 | 79,668,352 | 24.4 | 41,003 | 9,056 | 42.5 | 42 | 1,943 | 100 | 3,210,496 |
| Rat | 19 | 53,394,240 | 28.1 | 35,644 | 9,232 | 45.3 | 45 | 1,498 | 100 | 2,415,456 |
| Rat | 20 | 49,307,744 | 23.9 | 41,893 | 9,920 | 44.9 | 45 | 1,177 | 100 | 1,414,752 |
| Rat | X | 145,397,312 | 22.4 | 44,546 | 8,896 | 39.9 | 39 | 3,264 | 100 | 7,472,416 |
| Horse | 1 | 183,561,792 | 54.5 | 18,340 | 7,200 | 43.1 | 42 | 10,009 | 100 | 3,750,752 |
| Horse | 2 | 118,957,504 | 51.6 | 19,390 | 7,392 | 44.4 | 43 | 6,135 | 100 | 3,120,288 |
| Horse | 3 | 118,104,864 | 40.9 | 24,442 | 7,584 | 43.4 | 42 | 4,832 | 100 | 2,183,072 |
| Horse | 4 | 107,397,024 | 42.9 | 23,337 | 7,552 | 41.6 | 41 | 4,602 | 100 | 1,758,944 |
| Horse | 5 | 97,742,496 | 51.9 | 19,267 | 7,520 | 42.2 | 41 | 5,073 | 100 | 1,183,136 |
| Horse | 6 | 83,857,984 | 50.1 | 19,952 | 7,392 | 43.5 | 42 | 4,203 | 100 | 1,301,024 |
| Horse | 7 | 96,472,384 | 53.8 | 18,577 | 7,424 | 44.1 | 43 | 5,193 | 100 | 2,113,728 |
| Horse | 8 | 92,896,064 | 48.6 | 20,593 | 7,456 | 44.2 | 43 | 4,511 | 100 | 1,498,400 |
| Horse | 9 | 82,750,176 | 40.3 | 24,798 | 7,680 | 41.7 | 40 | 3,337 | 100 | 3,061,088 |
| Horse | 10 | 82,681,984 | 50.6 | 19,780 | 7,328 | 43.9 | 42 | 4,180 | 100 | 2,099,296 |
| Horse | 11 | 60,492,896 | 65.7 | 15,226 | 7,264 | 46.5 | 46 | 3,973 | 100 | 760,256 |
| Horse | 12 | 31,940,768 | 61.8 | 16,189 | 6,944 | 48.4 | 48 | 1,973 | 100 | 1,307,584 |
| Horse | 13 | 41,521,568 | 72.9 | 13,713 | 7,280 | 47.1 | 46 | 3,028 | 100 | 566,464 |
| Horse | 14 | 92,877,376 | 46.2 | 21,660 | 7,600 | 42.3 | 41 | 4,288 | 100 | 2,201,280 |
| Horse | 15 | 90,790,016 | 54.2 | 18,438 | 7,424 | 43 | 42 | 4,924 | 100 | 1,186,048 |
| Horse | 16 | 86,752,160 | 45.6 | 21,935 | 7,776 | 43 | 42 | 3,955 | 100 | 1,778,752 |
| Horse | 17 | 80,151,872 | 31.5 | 31,756 | 8,096 | 40.9 | 40 | 2,524 | 100 | 2,440,960 |
| Horse | 18 | 81,597,248 | 39.8 | 25,130 | 7,808 | 40.8 | 39 | 3,247 | 100 | 1,469,632 |
| Horse | 19 | 59,453,792 | 44.4 | 22,529 | 7,744 | 40.5 | 40 | 2,639 | 100 | 1,648,640 |
| Horse | 20 | 63,448,096 | 45.4 | 22,008 | 7,456 | 42.8 | 42 | 2,883 | 100 | 1,402,816 |
| Horse | 21 | 57,021,056 | 39.4 | 25,377 | 7,104 | 42.7 | 41 | 2,247 | 100 | 2,339,424 |
| Horse | 22 | 49,278,304 | 63.4 | 15,779 | 7,040 | 45.8 | 45 | 3,123 | 100 | 1,461,888 |
| Horse | 23 | 55,051,296 | 43.1 | 23,219 | 7,424 | 41.6 | 41 | 2,371 | 100 | 1,089,696 |
| Horse | 24 | 45,792,672 | 56.4 | 17,735 | 7,248 | 44.4 | 43 | 2,582 | 100 | 1,277,760 |
| Horse | 25 | 38,841,888 | 63.1 | 15,860 | 7,328 | 46.2 | 45 | 2,449 | 100 | 828,640 |
| Horse | 26 | 41,476,928 | 34 | 29,395 | 7,392 | 43.2 | 42 | 1,411 | 100 | 1,747,104 |
| Horse | 27 | 39,469,792 | 41.1 | 24,349 | 8,000 | 40.9 | 40 | 1,621 | 100 | 955,328 |
| Horse | 28 | 45,531,360 | 47.8 | 20,924 | 7,408 | 44.6 | 43 | 2,176 | 100 | 2,104,256 |
| Horse | 29 | 33,264,096 | 50.6 | 19,765 | 7,360 | 41.6 | 41 | 1,683 | 100 | 760,288 |
| Horse | 30 | 29,835,520 | 43.3 | 23,075 | 7,552 | 42.6 | 41 | 1,293 | 100 | 1,572,160 |
| Horse | 31 | 24,827,520 | 41.8 | 23,919 | 7,296 | 42.6 | 42 | 1,038 | 100 | 908,928 |
| Horse | X | 121,614,432 | 39.9 | 25,091 | 7,872 | 40.7 | 40 | 4,847 | 100 | 2,044,000 |
| Dog | 1 | 122,070,560 | 51.4 | 19,472 | 7,424 | 43.6 | 42 | 6,269 | 100 | 2,872,032 |
| Dog | 2 | 84,986,272 | 49.3 | 20,264 | 7,792 | 44.5 | 43 | 4,194 | 100 | 2,126,016 |
| Dog | 3 | 91,547,360 | 47.5 | 21,045 | 7,648 | 42.4 | 41 | 4,350 | 100 | 1,101,376 |
| Dog | 4 | 88,054,560 | 41.6 | 24,026 | 7,776 | 43.2 | 42 | 3,665 | 100 | 4,603,552 |
| Dog | 5 | 88,556,640 | 57.9 | 17,279 | 7,584 | 45.2 | 44 | 5,125 | 100 | 1,174,784 |
| Dog | 6 | 77,066,624 | 50.2 | 19,934 | 7,744 | 44.5 | 43 | 3,866 | 100 | 1,135,616 |
| Dog | 7 | 80,608,000 | 46.7 | 21,421 | 7,552 | 43.1 | 42 | 3,763 | 100 | 866,688 |
| Dog | 8 | 74,071,520 | 42.1 | 23,741 | 7,824 | 43.5 | 42 | 3,120 | 100 | 4,313,088 |
| Dog | 9 | 60,688,608 | 56.6 | 17,668 | 7,840 | 47.8 | 47 | 3,435 | 100 | 1,279,104 |
| Dog | 10 | 68,960,640 | 52 | 19,220 | 7,456 | 45 | 43 | 3,588 | 100 | 1,093,536 |
| Dog | 11 | 74,159,296 | 41 | 24,403 | 7,808 | 42.9 | 42 | 3,039 | 100 | 1,901,664 |
| Dog | 12 | 72,323,808 | 36.1 | 27,668 | 8,224 | 41.8 | 40 | 2,614 | 100 | 3,348,096 |
| Dog | 13 | 62,975,520 | 39.4 | 25,393 | 8,000 | 43.2 | 41 | 2,480 | 100 | 2,632,096 |
| Dog | 14 | 60,715,328 | 35.5 | 28,148 | 7,936 | 41.1 | 39 | 2,157 | 100 | 1,834,912 |
| Dog | 15 | 63,855,456 | 46.3 | 21,587 | 7,600 | 43.2 | 41 | 2,958 | 100 | 1,010,656 |
| Dog | 16 | 59,207,872 | 51 | 19,618 | 7,440 | 43 | 41 | 3,018 | 100 | 1,010,784 |
| Dog | 17 | 64,090,304 | 54.3 | 18,422 | 7,360 | 44.2 | 43 | 3,479 | 100 | 2,125,024 |
| Dog | 18 | 55,531,008 | 52 | 19,235 | 7,424 | 45.6 | 44 | 2,887 | 100 | 1,089,376 |
| Dog | 19 | 53,607,136 | 41.3 | 24,224 | 8,032 | 40.6 | 39 | 2,213 | 100 | 1,554,784 |
| Dog | 20 | 57,839,648 | 49.4 | 20,238 | 7,808 | 46.3 | 45 | 2,858 | 100 | 792,480 |
| Dog | 21 | 50,623,104 | 39.6 | 25,223 | 8,288 | 42 | 41 | 2,007 | 100 | 1,647,040 |
| Dog | 22 | 61,283,552 | 29 | 34,507 | 8,160 | 41.9 | 40 | 1,776 | 100 | 7,385,664 |
| Dog | 23 | 52,169,408 | 45.7 | 21,892 | 7,808 | 41.6 | 40 | 2,383 | 100 | 1,281,376 |
| Dog | 24 | 47,496,224 | 51.3 | 19,498 | 7,728 | 46.2 | 45 | 2,436 | 100 | 1,220,000 |
| Dog | 25 | 51,352,384 | 50 | 20,005 | 7,488 | 43.5 | 42 | 2,567 | 100 | 3,361,312 |
| Dog | 26 | 38,804,896 | 46.2 | 21,655 | 8,160 | 46.5 | 46 | 1,792 | 100 | 1,080,416 |
| Dog | 27 | 45,725,984 | 43.7 | 22,897 | 7,936 | 42.3 | 41 | 1,997 | 100 | 1,194,944 |
| Dog | 28 | 40,985,856 | 53.3 | 18,775 | 7,360 | 45.3 | 44 | 2,183 | 100 | 815,968 |
| Dog | 29 | 41,709,440 | 31.2 | 32,084 | 8,464 | 40.2 | 39 | 1,300 | 100 | 5,680,224 |
| Dog | 30 | 40,042,528 | 48.5 | 20,619 | 8,096 | 43.8 | 42 | 1,942 | 100 | 1,125,280 |
| Dog | 31 | 39,763,008 | 37.7 | 26,509 | 7,504 | 44 | 42 | 1,500 | 100 | 1,589,216 |
| Dog | 32 | 38,755,040 | 27.2 | 36,769 | 9,728 | 38.1 | 37 | 1,054 | 100 | 1,387,488 |
| Dog | 33 | 31,238,496 | 41.2 | 24,291 | 8,000 | 41.4 | 40 | 1,286 | 100 | 1,857,856 |
| Dog | 34 | 41,922,592 | 47.6 | 21,003 | 7,360 | 42.9 | 41 | 1,996 | 100 | 1,187,616 |
| Dog | 35 | 26,416,608 | 54.2 | 18,447 | 7,296 | 42.2 | 41 | 1,432 | 100 | 2,211,808 |
| Dog | 36 | 30,684,608 | 46.3 | 21,594 | 8,672 | 40 | 39 | 1,421 | 100 | 885,504 |
| Dog | 37 | 30,741,600 | 36.8 | 27,205 | 7,904 | 42.9 | 41 | 1,130 | 100 | 1,884,768 |
| Dog | 38 | 23,779,040 | 47.3 | 21,137 | 7,520 | 44.4 | 43 | 1,125 | 100 | 3,119,968 |
| Dog | X | 123,181,696 | 36.4 | 27,496 | 7,968 | 42.8 | 41 | 4,480 | 100 | 3,046,912 |
| Pig | 1 | 291,977,152 | 37.7 | 26,524 | 8,096 | 42.2 | 41 | 11,008 | 100 | 2,545,152 |
| Pig | 2 | 138,150,912 | 48.4 | 20,681 | 7,616 | 45 | 44 | 6,680 | 100 | 1,082,400 |
| Pig | 3 | 121,501,024 | 56.3 | 17,774 | 7,712 | 45.6 | 45 | 6,836 | 100 | 1,278,752 |
| Pig | 4 | 134,999,424 | 41.3 | 24,189 | 8,160 | 43.3 | 43 | 5,581 | 100 | 3,691,808 |
| Pig | 5 | 98,837,824 | 41.8 | 23,897 | 8,128 | 44.4 | 43 | 4,136 | 100 | 1,971,808 |
| Pig | 6 | 121,210,944 | 53.3 | 18,755 | 7,488 | 46.4 | 46 | 6,463 | 100 | 1,037,120 |
| Pig | 7 | 135,052,864 | 48.2 | 20,745 | 7,872 | 45 | 44 | 6,510 | 100 | 1,456,864 |
| Pig | 8 | 117,775,104 | 30.4 | 32,852 | 8,480 | 41.7 | 40 | 3,585 | 100 | 4,774,304 |
| Pig | 9 | 130,657,248 | 43.5 | 22,995 | 7,872 | 43.6 | 43 | 5,682 | 100 | 1,138,240 |
| Pig | 10 | 65,775,680 | 44.2 | 22,634 | 8,048 | 43.2 | 43 | 2,906 | 100 | 1,376,608 |
| Pig | 11 | 78,558,880 | 34.4 | 29,085 | 7,936 | 43.2 | 42 | 2,701 | 100 | 2,650,688 |
| Pig | 12 | 56,502,336 | 60.8 | 16,454 | 7,456 | 48.9 | 49 | 3,434 | 100 | 820,768 |
| Pig | 13 | 142,767,264 | 34.7 | 28,801 | 8,128 | 42.2 | 41 | 4,957 | 100 | 2,632,480 |
| Pig | 14 | 147,417,504 | 49.5 | 20,219 | 7,616 | 44.6 | 44 | 7,291 | 100 | 1,681,408 |
| Pig | 15 | 132,148,704 | 36.7 | 27,275 | 7,840 | 42.2 | 41 | 4,845 | 100 | 2,376,864 |
| Pig | 16 | 76,457,888 | 41.9 | 23,893 | 7,968 | 42.4 | 42 | 3,200 | 100 | 1,721,472 |
| Pig | 17 | 63,427,904 | 49.1 | 20,375 | 7,648 | 45.9 | 45 | 3,113 | 100 | 1,368,608 |
| Pig | 18 | 53,590,720 | 45.9 | 21,785 | 7,808 | 44.7 | 44 | 2,460 | 100 | 2,105,536 |
| Pig | X | 124,471,488 | 40.3 | 24,785 | 7,968 | 42.3 | 41 | 5,022 | 100 | 1,108,320 |
| Cow | 1 | 151,811,296 | 28.9 | 34,660 | 7,936 | 41.6 | 41 | 4,380 | 100 | 2,129,920 |
| Cow | 2 | 135,113,632 | 32.3 | 30,947 | 8,144 | 43 | 42 | 4,366 | 100 | 2,665,696 |
| Cow | 3 | 119,266,624 | 41 | 24,385 | 7,872 | 43.6 | 43 | 4,891 | 100 | 1,302,464 |
| Cow | 4 | 117,710,720 | 32.6 | 30,678 | 7,552 | 42.3 | 41 | 3,837 | 100 | 5,363,424 |
| Cow | 5 | 117,624,064 | 35.1 | 28,460 | 7,808 | 44.1 | 43 | 4,133 | 100 | 3,389,312 |
| Cow | 6 | 113,387,872 | 24.8 | 40,323 | 7,984 | 42.4 | 41 | 2,812 | 100 | 4,395,744 |
| Cow | 7 | 106,864,480 | 39.8 | 25,097 | 7,680 | 44.4 | 43 | 4,258 | 100 | 2,089,536 |
| Cow | 8 | 108,989,536 | 36.9 | 27,092 | 7,840 | 42.6 | 42 | 4,023 | 100 | 2,730,176 |
| Cow | 9 | 102,186,560 | 27.7 | 36,045 | 7,936 | 41 | 40 | 2,835 | 100 | 2,480,832 |
| Cow | 10 | 100,506,688 | 38.5 | 25,977 | 8,128 | 42.8 | 42 | 3,869 | 100 | 2,266,048 |
| Cow | 11 | 103,892,640 | 47.9 | 20,879 | 7,584 | 44.7 | 44 | 4,976 | 100 | 1,432,416 |
| Cow | 12 | 80,766,656 | 25 | 39,944 | 8,208 | 42.7 | 42 | 2,022 | 100 | 5,460,160 |
| Cow | 13 | 79,882,464 | 46.6 | 21,480 | 7,584 | 44.9 | 44 | 3,719 | 100 | 1,232,384 |
| Cow | 16 | 72,475,040 | 45.4 | 22,049 | 7,776 | 44.4 | 43 | 3,287 | 100 | 1,746,240 |
| Cow | 17 | 71,031,328 | 34.1 | 29,364 | 8,128 | 45.5 | 44 | 2,419 | 100 | 5,221,472 |
| Cow | 18 | 61,271,040 | 59.2 | 16,902 | 7,296 | 47.1 | 46 | 3,625 | 100 | 1,108,608 |
| Cow | 19 | 60,848,224 | 59.8 | 16,726 | 7,392 | 47.4 | 47 | 3,638 | 100 | 1,227,104 |
| Cow | 20 | 70,909,696 | 30.3 | 32,981 | 7,808 | 43.3 | 42 | 2,150 | 100 | 6,105,280 |
| Cow | 21 | 65,176,704 | 45.3 | 22,094 | 7,232 | 44.8 | 44 | 2,950 | 100 | 3,427,520 |
| Cow | 22 | 58,557,152 | 43 | 23,274 | 7,872 | 45.3 | 44 | 2,516 | 100 | 1,689,376 |
| Cow | 23 | 49,505,216 | 38.5 | 25,960 | 8,000 | 45.1 | 44 | 1,907 | 100 | 1,460,832 |
| Cow | 24 | 61,194,496 | 33.7 | 29,706 | 7,840 | 43.5 | 43 | 2,060 | 100 | 1,703,040 |
| Cow | 25 | 41,451,456 | 60.3 | 16,581 | 7,280 | 47.8 | 47 | 2,500 | 100 | 840,096 |
| Cow | 26 | 48,502,016 | 44.8 | 22,310 | 7,360 | 44.5 | 44 | 2,174 | 100 | 1,410,816 |
| Cow | 27 | 45,288,160 | 34.8 | 28,736 | 7,936 | 43.4 | 43 | 1,576 | 100 | 2,678,784 |
| Cow | 28 | 43,186,240 | 41.4 | 24,140 | 7,488 | 43.8 | 43 | 1,789 | 100 | 2,173,536 |
| Cow | 29 | 48,141,824 | 49.8 | 20,084 | 7,168 | 46.6 | 46 | 2,397 | 100 | 1,552,288 |
| Cow | X | 83,036,672 | 41.7 | 23,999 | 7,808 | 41.7 | 41 | 3,460 | 100 | 2,017,728 |
| Cow | Y | 38,719,968 | 59.7 | 16,747 | 9,120 | 42.6 | 41 | 2,312 | 100 | 444,384 |
| Opossum | 1 | 733,412,928 | 30.4 | 32,872 | 7,712 | 39.4 | 38 | 22,311 | 100 | 4,972,672 |
| Opossum | 2 | 527,981,664 | 32.2 | 31,014 | 7,616 | 40.3 | 39 | 17,024 | 100 | 7,789,664 |
| Opossum | 3 | 515,087,168 | 30.1 | 33,221 | 7,712 | 39.1 | 38 | 15,505 | 100 | 6,875,168 |
| Opossum | 4 | 422,697,408 | 31.1 | 32,159 | 7,648 | 39.6 | 38 | 13,144 | 100 | 5,873,632 |
| Opossum | 5 | 298,042,848 | 31.4 | 31,852 | 7,808 | 38.7 | 38 | 9,357 | 100 | 3,080,672 |
| Opossum | 6 | 284,524,512 | 33 | 30,298 | 7,648 | 39.7 | 39 | 9,391 | 100 | 6,446,304 |
| Opossum | 7 | 254,715,584 | 23.7 | 42,178 | 7,808 | 38.7 | 37 | 6,039 | 100 | 10,514,048 |
| Opossum | 8 | 303,158,720 | 33.7 | 29,634 | 7,424 | 40.3 | 39 | 10,230 | 100 | 5,223,456 |
| Opossum | X | 72,972,128 | 59.7 | 16,756 | 6,816 | 42.3 | 42 | 4,355 | 100 | 2,114,560 |
| Chicken | 1 | 195,191,328 | 37.5 | 26,665 | 8,832 | 41.4 | 41 | 7,320 | 100 | 2,855,424 |
| Chicken | 2 | 150,357,856 | 37.4 | 26,768 | 8,832 | 41.1 | 40 | 5,617 | 100 | 1,428,256 |
| Chicken | 3 | 110,204,896 | 37.8 | 26,453 | 9,424 | 41.7 | 41 | 4,166 | 100 | 1,768,480 |
| Chicken | 4 | 90,936,160 | 35.7 | 27,989 | 9,536 | 42 | 41 | 3,249 | 100 | 4,008,128 |
| Chicken | 5 | 59,512,608 | 41.7 | 23,958 | 9,152 | 42.6 | 41 | 2,484 | 100 | 1,401,248 |
| Chicken | 6 | 35,175,936 | 43.2 | 23,173 | 9,696 | 43.4 | 42 | 1,518 | 100 | 521,312 |
| Chicken | 7 | 36,212,288 | 38.8 | 25,756 | 9,792 | 42.4 | 41 | 1,406 | 100 | 1,799,808 |
| Chicken | 8 | 28,570,208 | 41.7 | 24,009 | 9,056 | 44.1 | 43 | 1,190 | 100 | 1,584,928 |
| Chicken | 9 | 23,541,728 | 41.8 | 23,949 | 10,048 | 44.5 | 43 | 983 | 100 | 579,168 |
| Chicken | 10 | 20,484,704 | 47 | 21,294 | 9,408 | 44.3 | 43 | 962 | 100 | 653,856 |
| Chicken | 11 | 20,811,168 | 42.4 | 23,569 | 9,952 | 44.6 | 43 | 883 | 100 | 589,600 |
| Chicken | 12 | 19,573,280 | 42.5 | 23,526 | 9,920 | 45.2 | 44 | 832 | 100 | 706,336 |
| Chicken | 13 | 17,932,000 | 43.4 | 23,019 | 10,336 | 46 | 45 | 779 | 100 | 1,065,024 |
| Chicken | 14 | 15,306,464 | 47 | 21,289 | 10,208 | 46.6 | 46 | 719 | 100 | 587,200 |
| Chicken | 15 | 12,585,504 | 41.8 | 23,927 | 10,336 | 47.2 | 46 | 526 | 100 | 809,216 |
| Chicken | 16 | 405,312 | 51.8 | 19,301 | 7,520 | 51.1 | 53 | 21 | 100 | 97,344 |
| Chicken | 17 | 10,337,984 | 51.8 | 19,287 | 9,456 | 47.8 | 47 | 536 | 100 | 324,448 |
| Chicken | 18 | 10,513,248 | 46.9 | 21,325 | 9,824 | 48.6 | 48 | 493 | 100 | 358,560 |
| Chicken | 19 | 9,570,432 | 52.1 | 19,179 | 9,600 | 48.1 | 47 | 499 | 100 | 306,368 |
| Chicken | 20 | 13,560,512 | 45.4 | 22,014 | 9,536 | 47 | 46 | 616 | 100 | 489,664 |
| Chicken | 21 | 6,656,928 | 50.9 | 19,637 | 10,176 | 47.8 | 47 | 339 | 100 | 300,928 |
| Chicken | 22 | 3,809,632 | 54.1 | 18,493 | 8,928 | 47.8 | 46 | 206 | 100 | 301,696 |
| Chicken | 23 | 5,248,000 | 61.7 | 16,198 | 8,480 | 50.3 | 49 | 324 | 100 | 272,256 |
| Chicken | 24 | 6,138,368 | 41.1 | 24,359 | 10,928 | 49.6 | 49 | 252 | 100 | 533,600 |
| Chicken | 25 | 1,367,840 | 81.9 | 12,213 | 7,840 | 53.6 | 54 | 112 | 100 | 89,568 |
| Chicken | 26 | 4,830,272 | 49.9 | 20,043 | 9,344 | 50.9 | 51 | 241 | 100 | 482,528 |
| Chicken | 27 | 4,538,432 | 54.4 | 18,374 | 8,192 | 50.2 | 49 | 247 | 100 | 649,984 |
| Chicken | 28 | 3,711,296 | 60.4 | 16,568 | 8,416 | 51.2 | 51 | 224 | 100 | 226,304 |
| Chicken | W | 233,824 | 42.8 | 23,382 | 9,008 | 41.2 | 43 | 10 | 100 | 107,136 |
| Chicken | Z | 67,535,552 | 39.9 | 25,060 | 8,288 | 41.4 | 40 | 2,695 | 100 | 1,215,264 |

(b) Homogeneous domains

| **Species** | **Chr** | **Chr size** | **Density (per Mb)** | **Mean size (bp)** | **Median size (bp)** | **Mean GC%** | **Median GC%** | **#Domains** | **Genome coverage (%)** | **Longes**t **domain** |
| --- | --- | --- | --- | --- | --- | --- | --- | --- | --- | --- |
| Human | 1 | 217,916,736 | 30.6 | 27,164 | 8,352 | 42.8 | 42 | 6,667 | 83 | 2,875,136 |
| Human | 2 | 233,385,152 | 27 | 28,457 | 8,192 | 41 | 40 | 6,292 | 77 | 1,864,160 |
| Human | 3 | 194,309,312 | 20.9 | 37,617 | 9,216 | 40.4 | 39 | 4,065 | 79 | 3,243,616 |
| Human | 4 | 185,864,768 | 16.2 | 49,152 | 8,864 | 38.9 | 37 | 3,011 | 80 | 3,790,592 |
| Human | 5 | 174,600,768 | 23.4 | 34,661 | 8,656 | 40 | 39 | 4,080 | 81 | 3,259,072 |
| Human | 6 | 167,628,544 | 21.2 | 35,568 | 8,544 | 39.7 | 38 | 3,547 | 75 | 2,701,312 |
| Human | 7 | 151,582,880 | 25.8 | 31,669 | 8,416 | 41.8 | 40 | 3,909 | 82 | 2,859,264 |
| Human | 8 | 140,746,368 | 25.3 | 31,715 | 8,224 | 41.3 | 40 | 3,563 | 80 | 2,484,160 |
| Human | 9 | 109,434,656 | 33.1 | 24,717 | 8,544 | 42.8 | 41 | 3,624 | 82 | 1,134,400 |
| Human | 10 | 128,196,704 | 33 | 23,390 | 7,904 | 42.3 | 41 | 4,226 | 77 | 2,046,944 |
| Human | 11 | 130,274,560 | 34 | 25,417 | 8,288 | 43.4 | 42 | 4,434 | 87 | 1,807,808 |
| Human | 12 | 129,843,424 | 24.1 | 33,519 | 8,896 | 41.5 | 40 | 3,127 | 81 | 2,730,848 |
| Human | 13 | 94,957,600 | 19.5 | 38,804 | 8,800 | 38.3 | 37 | 1,856 | 76 | 4,510,208 |
| Human | 14 | 86,964,576 | 27 | 30,481 | 8,448 | 42.5 | 41 | 2,351 | 82 | 1,328,128 |
| Human | 15 | 77,770,144 | 33.2 | 21,965 | 7,840 | 42.7 | 42 | 2,580 | 73 | 1,032,608 |
| Human | 16 | 74,960,800 | 34 | 24,082 | 8,592 | 45.8 | 45 | 2,550 | 82 | 1,182,560 |
| Human | 17 | 75,513,088 | 38.9 | 21,729 | 8,256 | 47.3 | 47 | 2,934 | 84 | 1,347,680 |
| Human | 18 | 74,291,328 | 21.3 | 34,327 | 8,480 | 39.5 | 38 | 1,585 | 73 | 1,339,392 |
| Human | 19 | 54,533,856 | 45.9 | 18,465 | 8,224 | 49.4 | 50 | 2,502 | 85 | 686,112 |
| Human | 20 | 59,266,464 | 32.2 | 24,976 | 7,968 | 45.2 | 45 | 1,911 | 81 | 5,243,904 |
| Human | 21 | 33,211,648 | 33.1 | 26,418 | 7,632 | 42.7 | 41 | 1,100 | 87 | 842,688 |
| Human | 22 | 33,725,632 | 48 | 16,767 | 7,504 | 49.4 | 50 | 1,618 | 80 | 487,808 |
| Human | X | 135,172,992 | 19.2 | 38,347 | 8,256 | 40.1 | 39 | 2,595 | 74 | 4,823,840 |
| Human | Y | 18,186,240 | 24.9 | 25,868 | 8,512 | 40.1 | 39 | 452 | 64 | 489,472 |
| Chimpanzee | 1 | 217,189,792 | 31.5 | 26,265 | 8,480 | 42.9 | 42 | 6,837 | 83 | 2,890,400 |
| Chimpanzee | 2A | 105,873,440 | 28.9 | 25,296 | 8,000 | 41.9 | 41 | 3,065 | 73 | 2,945,760 |
| Chimpanzee | 2B | 127,869,088 | 24 | 33,287 | 8,640 | 40.4 | 39 | 3,072 | 80 | 1,896,704 |
| Chimpanzee | 3 | 194,962,656 | 22 | 35,956 | 9,216 | 40.3 | 39 | 4,296 | 79 | 2,527,744 |
| Chimpanzee | 4 | 186,955,680 | 16.8 | 46,053 | 9,296 | 38.6 | 37 | 3,150 | 78 | 6,451,104 |
| Chimpanzee | 5 | 175,225,344 | 22.7 | 35,167 | 8,912 | 40.2 | 39 | 3,982 | 80 | 4,092,448 |
| Chimpanzee | 6 | 164,697,664 | 20.6 | 36,270 | 8,784 | 39.5 | 38 | 3,386 | 75 | 3,734,208 |
| Chimpanzee | 7 | 151,069,600 | 26.6 | 30,105 | 8,512 | 41.4 | 40 | 4,026 | 80 | 3,055,232 |
| Chimpanzee | 8 | 138,150,976 | 26 | 30,138 | 8,480 | 40.8 | 40 | 3,593 | 78 | 2,512,032 |
| Chimpanzee | 9 | 109,295,936 | 30.8 | 27,074 | 8,448 | 42.6 | 41 | 3,366 | 83 | 2,176,352 |
| Chimpanzee | 10 | 125,696,576 | 31.3 | 24,481 | 8,128 | 42 | 41 | 3,934 | 77 | 2,019,232 |
| Chimpanzee | 11 | 123,596,640 | 34.4 | 24,900 | 8,288 | 43.8 | 43 | 4,249 | 86 | 2,789,280 |
| Chimpanzee | 12 | 129,867,936 | 24.6 | 32,160 | 8,832 | 41.4 | 40 | 3,194 | 79 | 2,485,024 |
| Chimpanzee | 13 | 87,794,784 | 18.2 | 42,269 | 8,496 | 38.3 | 37 | 1,600 | 77 | 2,574,464 |
| Chimpanzee | 14 | 86,251,392 | 27.7 | 29,099 | 8,320 | 42.6 | 41 | 2,385 | 80 | 2,124,480 |
| Chimpanzee | 15 | 76,971,936 | 30.7 | 24,319 | 7,776 | 42.7 | 42 | 2,365 | 75 | 2,496,064 |
| Chimpanzee | 16 | 74,505,664 | 34.3 | 23,903 | 8,832 | 45.5 | 45 | 2,555 | 82 | 1,239,136 |
| Chimpanzee | 17 | 73,429,760 | 38.7 | 21,441 | 8,336 | 47.4 | 48 | 2,842 | 83 | 1,173,408 |
| Chimpanzee | 18 | 74,181,056 | 21.4 | 33,911 | 8,608 | 39.6 | 39 | 1,584 | 72 | 1,574,752 |
| Chimpanzee | 19 | 51,997,056 | 45.9 | 18,153 | 8,064 | 49 | 49 | 2,388 | 83 | 967,360 |
| Chimpanzee | 20 | 58,102,080 | 30.9 | 25,985 | 8,224 | 44.8 | 45 | 1,794 | 80 | 5,290,400 |
| Chimpanzee | 21 | 32,706,016 | 30.8 | 28,732 | 7,664 | 43 | 41 | 1,006 | 88 | 1,040,576 |
| Chimpanzee | 22 | 32,341,152 | 46.8 | 17,669 | 7,680 | 49.2 | 50 | 1,512 | 83 | 611,072 |
| Chimpanzee | X | 130,929,824 | 17.7 | 41,978 | 8,832 | 39.8 | 39 | 2,313 | 74 | 2,963,680 |
| Chimpanzee | Y | 22,691,168 | 29.9 | 20,186 | 7,984 | 41.5 | 41 | 678 | 60 | 544,128 |
| Orangutan | 1 | 216,060,736 | 32.2 | 25,503 | 8,352 | 42.7 | 42 | 6,962 | 82 | 3,571,680 |
| Orangutan | 2A | 104,616,320 | 28.2 | 26,063 | 8,128 | 41.8 | 41 | 2,947 | 73 | 1,534,944 |
| Orangutan | 2B | 126,496,928 | 24.5 | 32,448 | 8,544 | 40.1 | 38 | 3,095 | 79 | 1,796,768 |
| Orangutan | 3 | 190,355,264 | 20.1 | 39,192 | 9,360 | 40.2 | 39 | 3,822 | 79 | 2,245,184 |
| Orangutan | 4 | 186,135,648 | 17.5 | 45,371 | 9,200 | 38.5 | 37 | 3,252 | 79 | 4,315,296 |
| Orangutan | 5 | 172,726,976 | 21.5 | 36,908 | 8,704 | 39.8 | 38 | 3,712 | 79 | 3,532,000 |
| Orangutan | 6 | 164,088,672 | 17.9 | 40,804 | 9,248 | 39.5 | 38 | 2,945 | 73 | 2,809,664 |
| Orangutan | 7 | 145,686,624 | 27.6 | 29,569 | 8,416 | 41.2 | 40 | 4,014 | 81 | 2,257,312 |
| Orangutan | 8 | 140,594,368 | 24.9 | 32,201 | 8,800 | 41 | 40 | 3,503 | 80 | 2,616,224 |
| Orangutan | 9 | 109,204,832 | 30.7 | 26,551 | 8,384 | 42.6 | 41 | 3,357 | 82 | 2,453,120 |
| Orangutan | 10 | 124,371,648 | 31 | 25,318 | 8,032 | 42.2 | 41 | 3,861 | 79 | 2,168,032 |
| Orangutan | 11 | 124,382,944 | 34.5 | 24,887 | 8,256 | 43.2 | 42 | 4,288 | 86 | 1,835,904 |
| Orangutan | 12 | 128,777,280 | 26.9 | 29,604 | 8,544 | 41.3 | 40 | 3,468 | 80 | 2,170,048 |
| Orangutan | 13 | 94,686,048 | 17.3 | 44,958 | 9,216 | 38.4 | 37 | 1,635 | 78 | 3,623,136 |
| Orangutan | 14 | 86,577,920 | 27.2 | 30,219 | 8,640 | 41.9 | 40 | 2,356 | 82 | 1,752,128 |
| Orangutan | 15 | 75,961,056 | 33.8 | 21,245 | 8,416 | 42.4 | 41 | 2,565 | 72 | 983,680 |
| Orangutan | 16 | 70,802,048 | 35 | 23,670 | 9,024 | 45.5 | 44 | 2,476 | 83 | 1,024,512 |
| Orangutan | 17 | 67,081,376 | 40.6 | 20,482 | 8,512 | 47.3 | 47 | 2,724 | 83 | 2,953,984 |
| Orangutan | 18 | 73,515,712 | 22.6 | 31,921 | 8,608 | 39.6 | 39 | 1,665 | 72 | 1,482,720 |
| Orangutan | 19 | 51,367,232 | 44.6 | 18,884 | 8,064 | 49 | 49 | 2,292 | 84 | 687,712 |
| Orangutan | 20 | 58,061,408 | 38.4 | 20,799 | 7,840 | 44.5 | 44 | 2,229 | 80 | 1,316,800 |
| Orangutan | 21 | 33,052,576 | 29 | 29,750 | 7,712 | 42.9 | 42 | 957 | 86 | 2,045,216 |
| Orangutan | 22 | 30,217,376 | 47.4 | 17,220 | 7,712 | 49.3 | 50 | 1,431 | 82 | 515,936 |
| Orangutan | X | 148,146,336 | 19.1 | 39,581 | 8,640 | 39.9 | 39 | 2,828 | 76 | 2,110,048 |
| Mouse | 1 | 191,477,376 | 13 | 60,681 | 11,104 | 43.2 | 43 | 2,495 | 79 | 5,166,272 |
| Mouse | 2 | 178,392,032 | 18.5 | 45,579 | 11,840 | 43.6 | 43 | 3,296 | 84 | 2,878,336 |
| Mouse | 3 | 156,393,856 | 12.3 | 61,791 | 11,264 | 41.1 | 41 | 1,919 | 76 | 3,088,992 |
| Mouse | 4 | 151,886,784 | 20.2 | 41,843 | 10,656 | 43.8 | 44 | 3,067 | 84 | 2,657,824 |
| Mouse | 5 | 147,721,152 | 16.5 | 53,360 | 11,600 | 44.3 | 45 | 2,432 | 88 | 3,583,136 |
| Mouse | 6 | 146,316,992 | 14.6 | 55,518 | 11,312 | 42.9 | 43 | 2,138 | 81 | 4,604,448 |
| Mouse | 7 | 141,878,176 | 21.7 | 36,910 | 9,984 | 44.4 | 45 | 3,074 | 80 | 3,184,416 |
| Mouse | 8 | 124,796,736 | 16.5 | 51,535 | 11,648 | 43.5 | 43 | 2,059 | 85 | 2,590,048 |
| Mouse | 9 | 120,720,160 | 18.7 | 40,584 | 10,016 | 44.5 | 45 | 2,258 | 76 | 1,519,232 |
| Mouse | 10 | 126,847,808 | 14.4 | 56,781 | 11,664 | 42.1 | 41 | 1,826 | 82 | 4,642,368 |
| Mouse | 11 | 118,743,520 | 20.7 | 37,881 | 11,008 | 45.9 | 46 | 2,459 | 78 | 2,125,216 |
| Mouse | 12 | 117,459,264 | 17.7 | 45,171 | 10,720 | 43.7 | 44 | 2,075 | 80 | 3,654,432 |
| Mouse | 13 | 116,370,848 | 13.5 | 52,574 | 11,024 | 43 | 43 | 1,568 | 71 | 2,377,728 |
| Mouse | 14 | 121,635,264 | 14 | 49,462 | 10,432 | 42.9 | 43 | 1,698 | 69 | 3,683,104 |
| Mouse | 15 | 100,439,936 | 19.1 | 44,442 | 9,904 | 44.2 | 44 | 1,920 | 85 | 2,246,272 |
| Mouse | 16 | 95,004,896 | 15.9 | 50,568 | 12,096 | 42.4 | 42 | 1,515 | 81 | 1,986,432 |
| Mouse | 17 | 91,898,144 | 20.1 | 43,221 | 10,624 | 43.9 | 44 | 1,846 | 87 | 4,089,664 |
| Mouse | 18 | 87,600,032 | 12 | 61,433 | 12,912 | 42.5 | 42 | 1,048 | 73 | 2,001,440 |
| Mouse | 19 | 58,142,176 | 16.5 | 46,449 | 11,456 | 44.3 | 44 | 958 | 77 | 1,282,016 |
| Mouse | X | 162,080,832 | 12.9 | 46,043 | 9,376 | 40.9 | 40 | 2,096 | 60 | 3,620,864 |
| Mouse | Y | 2,702,496 | 13.3 | 34,083 | 11,760 | 40.6 | 41 | 36 | 45 | 218,624 |
| Rat | 1 | 242,568,192 | 17.4 | 46,795 | 10,720 | 43.8 | 44 | 4,215 | 81 | 4,724,576 |
| Rat | 2 | 235,352,352 | 11.1 | 65,745 | 12,992 | 41.4 | 41 | 2,612 | 73 | 2,533,696 |
| Rat | 3 | 157,225,152 | 19.2 | 44,833 | 11,008 | 44.4 | 45 | 3,022 | 86 | 1,713,856 |
| Rat | 4 | 172,528,416 | 15 | 55,046 | 10,752 | 43.4 | 43 | 2,588 | 83 | 2,769,728 |
| Rat | 5 | 157,560,736 | 16.8 | 51,204 | 11,744 | 43.8 | 43 | 2,641 | 86 | 3,627,200 |
| Rat | 6 | 134,552,608 | 16.8 | 48,313 | 10,976 | 43.8 | 44 | 2,267 | 81 | 3,441,056 |
| Rat | 7 | 131,647,424 | 20.3 | 42,323 | 10,784 | 43.9 | 43 | 2,675 | 86 | 4,682,912 |
| Rat | 8 | 118,069,792 | 18.2 | 41,672 | 11,072 | 44.3 | 45 | 2,151 | 76 | 1,555,616 |
| Rat | 9 | 104,712,416 | 13.7 | 60,307 | 11,472 | 43.5 | 43 | 1,436 | 83 | 2,263,936 |
| Rat | 10 | 101,026,080 | 20.7 | 36,367 | 10,976 | 47.1 | 48 | 2,093 | 75 | 1,567,840 |
| Rat | 11 | 81,764,224 | 13.3 | 64,382 | 12,624 | 42.6 | 42 | 1,086 | 86 | 3,508,672 |
| Rat | 12 | 41,216,160 | 14.8 | 45,016 | 11,520 | 47 | 48 | 608 | 66 | 1,624,864 |
| Rat | 13 | 102,334,624 | 14.3 | 55,778 | 11,216 | 42.3 | 42 | 1,464 | 80 | 7,583,552 |
| Rat | 14 | 100,801,632 | 15.9 | 50,718 | 11,616 | 42.9 | 42 | 1,599 | 80 | 1,683,296 |
| Rat | 15 | 98,895,328 | 13.7 | 51,990 | 11,920 | 42.9 | 43 | 1,352 | 71 | 1,359,904 |
| Rat | 16 | 81,355,456 | 17.8 | 46,813 | 11,136 | 43 | 43 | 1,449 | 83 | 2,278,304 |
| Rat | 17 | 87,674,496 | 12.7 | 59,078 | 12,608 | 43.5 | 44 | 1,117 | 75 | 4,717,408 |
| Rat | 18 | 79,668,352 | 13.5 | 56,235 | 11,776 | 42.7 | 42 | 1,079 | 76 | 3,210,496 |
| Rat | 19 | 53,394,240 | 18.2 | 44,404 | 11,232 | 45.8 | 46 | 973 | 81 | 2,415,456 |
| Rat | 20 | 49,307,744 | 17.3 | 52,094 | 11,328 | 45.1 | 45 | 853 | 90 | 1,414,752 |
| Rat | X | 145,397,312 | 11.5 | 52,579 | 10,144 | 40 | 39 | 1,665 | 60 | 2,678,432 |
| Horse | 1 | 183,561,792 | 40.7 | 20,992 | 7,840 | 42.4 | 41 | 7,473 | 85 | 3,750,752 |
| Horse | 2 | 118,957,504 | 44.3 | 21,181 | 7,904 | 44.1 | 42 | 5,272 | 94 | 3,120,288 |
| Horse | 3 | 118,104,864 | 32.1 | 28,389 | 8,224 | 42.6 | 41 | 3,786 | 91 | 2,183,072 |
| Horse | 4 | 107,397,024 | 27.7 | 29,464 | 8,768 | 40.4 | 39 | 2,970 | 81 | 1,758,944 |
| Horse | 5 | 97,742,496 | 37 | 22,381 | 8,352 | 41.5 | 41 | 3,616 | 83 | 1,183,136 |
| Horse | 6 | 83,857,984 | 38.5 | 23,079 | 8,000 | 42.9 | 41 | 3,227 | 89 | 1,301,024 |
| Horse | 7 | 96,472,384 | 45 | 20,294 | 7,872 | 43.5 | 42 | 4,346 | 91 | 2,113,728 |
| Horse | 8 | 92,896,064 | 38.2 | 23,145 | 7,808 | 43.4 | 42 | 3,545 | 88 | 1,498,400 |
| Horse | 9 | 82,750,176 | 28 | 30,551 | 8,512 | 40.8 | 39 | 2,315 | 85 | 3,061,088 |
| Horse | 10 | 82,681,984 | 39.3 | 22,495 | 7,744 | 43.1 | 41 | 3,252 | 88 | 2,099,296 |
| Horse | 11 | 60,492,896 | 56.3 | 16,259 | 7,616 | 46.5 | 46 | 3,403 | 91 | 760,256 |
| Horse | 12 | 31,940,768 | 56.6 | 17,084 | 7,296 | 48.4 | 48 | 1,809 | 97 | 1,307,584 |
| Horse | 13 | 41,521,568 | 60.8 | 14,765 | 7,680 | 46.9 | 46 | 2,525 | 90 | 566,464 |
| Horse | 14 | 92,877,376 | 33.3 | 25,692 | 8,544 | 41.3 | 40 | 3,091 | 86 | 2,201,280 |
| Horse | 15 | 90,790,016 | 37.5 | 21,593 | 8,064 | 42.1 | 41 | 3,401 | 81 | 1,186,048 |
| Horse | 16 | 86,752,160 | 32.3 | 25,554 | 8,384 | 42.2 | 41 | 2,805 | 83 | 1,778,752 |
| Horse | 17 | 80,151,872 | 17.7 | 45,239 | 9,152 | 38.7 | 38 | 1,415 | 80 | 2,440,960 |
| Horse | 18 | 81,597,248 | 24.9 | 33,099 | 9,024 | 39 | 37 | 2,033 | 82 | 1,469,632 |
| Horse | 19 | 59,453,792 | 25.3 | 28,261 | 8,928 | 39.3 | 38 | 1,504 | 71 | 1,648,640 |
| Horse | 20 | 63,448,096 | 31.2 | 26,370 | 8,192 | 41.8 | 40 | 1,980 | 82 | 1,402,816 |
| Horse | 21 | 57,021,056 | 29.1 | 30,519 | 7,520 | 41.7 | 40 | 1,657 | 89 | 2,339,424 |
| Horse | 22 | 49,278,304 | 49.8 | 17,813 | 7,552 | 45.6 | 45 | 2,456 | 89 | 1,461,888 |
| Horse | 23 | 55,051,296 | 27.1 | 28,564 | 8,368 | 40.2 | 39 | 1,494 | 78 | 1,089,696 |
| Horse | 24 | 45,792,672 | 45.2 | 19,959 | 7,936 | 44 | 43 | 2,069 | 90 | 1,277,760 |
| Horse | 25 | 38,841,888 | 54.9 | 17,075 | 7,776 | 46 | 45 | 2,131 | 94 | 828,640 |
| Horse | 26 | 41,476,928 | 24.5 | 35,830 | 8,128 | 41.8 | 40 | 1,015 | 88 | 1,747,104 |
| Horse | 27 | 39,469,792 | 24.4 | 30,744 | 8,816 | 39.2 | 38 | 964 | 75 | 955,328 |
| Horse | 28 | 45,531,360 | 41.1 | 22,833 | 7,840 | 44.3 | 42 | 1,872 | 94 | 2,104,256 |
| Horse | 29 | 33,264,096 | 30.2 | 23,663 | 7,872 | 39.9 | 39 | 1,005 | 71 | 760,288 |
| Horse | 30 | 29,835,520 | 32.1 | 27,460 | 8,288 | 41.6 | 39 | 959 | 88 | 1,572,160 |
| Horse | 31 | 24,827,520 | 25.7 | 30,303 | 8,704 | 41.5 | 40 | 639 | 78 | 908,928 |
| Horse | X | 121,614,432 | 25.8 | 31,302 | 8,768 | 39.5 | 38 | 3,140 | 81 | 2,044,000 |
| Dog | 1 | 122,070,560 | 35.1 | 22,461 | 8,192 | 42.3 | 40 | 4,287 | 79 | 2,872,032 |
| Dog | 2 | 84,986,272 | 36.1 | 22,661 | 8,544 | 43.6 | 42 | 3,066 | 82 | 2,126,016 |
| Dog | 3 | 91,547,360 | 33.7 | 23,707 | 8,416 | 41.4 | 40 | 3,086 | 80 | 1,101,376 |
| Dog | 4 | 88,054,560 | 29.1 | 28,432 | 8,448 | 42.2 | 41 | 2,566 | 83 | 4,603,552 |
| Dog | 5 | 88,556,640 | 43.7 | 18,633 | 8,064 | 44.9 | 44 | 3,866 | 81 | 1,174,784 |
| Dog | 6 | 77,066,624 | 38.1 | 22,188 | 8,192 | 43.9 | 42 | 2,937 | 85 | 1,135,616 |
| Dog | 7 | 80,608,000 | 33.4 | 24,549 | 8,192 | 42.2 | 40 | 2,692 | 82 | 866,688 |
| Dog | 8 | 74,071,520 | 30.6 | 28,142 | 8,592 | 42.6 | 41 | 2,266 | 86 | 4,313,088 |
| Dog | 9 | 60,688,608 | 44.8 | 19,017 | 8,096 | 48.1 | 48 | 2,719 | 85 | 1,279,104 |
| Dog | 10 | 68,960,640 | 38.3 | 21,940 | 8,128 | 44 | 42 | 2,638 | 84 | 1,093,536 |
| Dog | 11 | 74,159,296 | 27.2 | 28,608 | 8,320 | 41.8 | 40 | 2,020 | 78 | 1,901,664 |
| Dog | 12 | 72,323,808 | 23.5 | 34,096 | 9,280 | 40.2 | 38 | 1,700 | 80 | 3,348,096 |
| Dog | 13 | 62,975,520 | 28.8 | 30,903 | 9,120 | 41.8 | 40 | 1,811 | 89 | 2,632,096 |
| Dog | 14 | 60,715,328 | 21.4 | 35,881 | 9,840 | 39.4 | 38 | 1,300 | 77 | 1,662,752 |
| Dog | 15 | 63,855,456 | 31.7 | 25,593 | 8,288 | 41.8 | 39 | 2,026 | 81 | 1,010,656 |
| Dog | 16 | 59,207,872 | 35.4 | 22,458 | 7,968 | 41.6 | 40 | 2,098 | 80 | 1,010,784 |
| Dog | 17 | 64,090,304 | 38.9 | 20,236 | 7,776 | 43.4 | 42 | 2,490 | 79 | 2,125,024 |
| Dog | 18 | 55,531,008 | 41.1 | 21,557 | 7,968 | 45.1 | 43 | 2,285 | 89 | 1,089,376 |
| Dog | 19 | 53,607,136 | 24.4 | 32,474 | 9,504 | 38.6 | 37 | 1,309 | 79 | 1,554,784 |
| Dog | 20 | 57,839,648 | 38.9 | 22,219 | 8,256 | 46 | 45 | 2,249 | 86 | 792,480 |
| Dog | 21 | 50,623,104 | 26.4 | 30,739 | 9,424 | 41.2 | 40 | 1,334 | 81 | 1,647,040 |
| Dog | 22 | 61,283,552 | 15.9 | 51,683 | 9,760 | 39.2 | 38 | 977 | 82 | 7,385,664 |
| Dog | 23 | 52,169,408 | 29 | 24,912 | 8,832 | 40.2 | 39 | 1,515 | 72 | 840,896 |
| Dog | 24 | 47,496,224 | 38.6 | 21,352 | 8,416 | 45.9 | 45 | 1,834 | 82 | 1,220,000 |
| Dog | 25 | 51,352,384 | 38.2 | 22,364 | 8,000 | 42.5 | 41 | 1,961 | 85 | 3,361,312 |
| Dog | 26 | 38,804,896 | 32.3 | 24,177 | 8,960 | 46.7 | 46 | 1,255 | 78 | 1,080,416 |
| Dog | 27 | 45,725,984 | 30.2 | 26,974 | 8,768 | 41.3 | 39 | 1,380 | 81 | 1,194,944 |
| Dog | 28 | 40,985,856 | 39.4 | 20,721 | 7,936 | 44.5 | 43 | 1,615 | 82 | 815,968 |
| Dog | 29 | 41,709,440 | 16.5 | 46,975 | 10,128 | 37.4 | 37 | 690 | 78 | 5,680,224 |
| Dog | 30 | 40,042,528 | 33.9 | 23,693 | 8,672 | 43.1 | 41 | 1,356 | 80 | 1,125,280 |
| Dog | 31 | 39,763,008 | 27.2 | 32,596 | 8,320 | 42.3 | 40 | 1,082 | 89 | 1,589,216 |
| Dog | 32 | 38,755,040 | 14.3 | 43,203 | 12,352 | 36 | 36 | 556 | 62 | 1,226,528 |
| Dog | 33 | 31,238,496 | 24.9 | 30,105 | 9,248 | 38.9 | 38 | 777 | 75 | 1,857,856 |
| Dog | 34 | 41,922,592 | 30.5 | 25,575 | 8,320 | 41.3 | 40 | 1,280 | 78 | 1,187,616 |
| Dog | 35 | 26,416,608 | 34.6 | 18,709 | 7,744 | 40.4 | 40 | 915 | 65 | 617,984 |
| Dog | 36 | 30,684,608 | 27.4 | 24,663 | 9,984 | 37.3 | 37 | 840 | 68 | 885,504 |
| Dog | 37 | 30,741,600 | 24 | 33,121 | 9,136 | 41.1 | 39 | 738 | 80 | 1,884,768 |
| Dog | 38 | 23,779,040 | 36.3 | 24,411 | 8,224 | 43.8 | 42 | 863 | 89 | 3,119,968 |
| Dog | X | 123,181,696 | 22.4 | 34,611 | 8,880 | 41.9 | 40 | 2,762 | 78 | 3,046,912 |
| Pig | 1 | 291,977,152 | 25.7 | 32,256 | 9,056 | 41.5 | 40 | 7,515 | 83 | 2,545,152 |
| Pig | 2 | 138,150,912 | 40.3 | 23,053 | 8,128 | 44.9 | 44 | 5,563 | 93 | 1,082,400 |
| Pig | 3 | 121,501,024 | 45.4 | 19,614 | 8,352 | 45.4 | 44 | 5,522 | 89 | 1,278,752 |
| Pig | 4 | 134,999,424 | 29.7 | 27,926 | 9,024 | 42.9 | 42 | 4,015 | 83 | 3,691,808 |
| Pig | 5 | 98,837,824 | 32.2 | 28,043 | 9,056 | 44 | 42 | 3,184 | 90 | 1,971,808 |
| Pig | 6 | 121,210,944 | 44.6 | 20,804 | 8,032 | 46.4 | 46 | 5,400 | 93 | 1,037,120 |
| Pig | 7 | 135,052,864 | 37 | 23,523 | 8,448 | 44.7 | 44 | 5,000 | 87 | 1,456,864 |
| Pig | 8 | 117,775,104 | 19.2 | 42,503 | 9,824 | 40.2 | 39 | 2,264 | 82 | 4,774,304 |
| Pig | 9 | 130,657,248 | 31.8 | 27,052 | 8,640 | 43.4 | 42 | 4,155 | 86 | 1,138,240 |
| Pig | 10 | 65,775,680 | 29.1 | 27,018 | 8,672 | 42.3 | 41 | 1,913 | 79 | 1,376,608 |
| Pig | 11 | 78,558,880 | 24.2 | 35,140 | 8,896 | 41.8 | 41 | 1,902 | 85 | 2,650,688 |
| Pig | 12 | 56,502,336 | 50.3 | 18,112 | 7,952 | 49.2 | 49 | 2,844 | 91 | 820,768 |
| Pig | 13 | 142,767,264 | 22.2 | 36,214 | 8,960 | 41.8 | 40 | 3,164 | 80 | 2,632,480 |
| Pig | 14 | 147,417,504 | 37.3 | 23,043 | 8,320 | 44.6 | 44 | 5,499 | 86 | 1,681,408 |
| Pig | 15 | 132,148,704 | 24.8 | 33,447 | 8,416 | 41.2 | 39 | 3,274 | 83 | 2,376,864 |
| Pig | 16 | 76,457,888 | 28.9 | 28,466 | 8,768 | 41.5 | 41 | 2,209 | 82 | 1,721,472 |
| Pig | 17 | 63,427,904 | 35.7 | 23,587 | 8,224 | 46 | 46 | 2,262 | 84 | 1,368,608 |
| Pig | 18 | 53,590,720 | 34.1 | 24,863 | 8,512 | 44.3 | 43 | 1,828 | 85 | 2,105,536 |
| Pig | X | 124,471,488 | 26.8 | 30,047 | 8,768 | 41.2 | 40 | 3,337 | 81 | 1,108,320 |
| Cow | 1 | 151,811,296 | 17.5 | 47,528 | 9,248 | 41 | 40 | 2,654 | 83 | 2,129,920 |
| Cow | 2 | 135,113,632 | 23.1 | 38,712 | 9,280 | 42.7 | 42 | 3,119 | 89 | 2,665,696 |
| Cow | 3 | 119,266,624 | 30.9 | 28,651 | 8,800 | 43.3 | 42 | 3,689 | 89 | 1,302,464 |
| Cow | 4 | 117,710,720 | 21.2 | 40,244 | 8,704 | 41.5 | 41 | 2,491 | 85 | 5,363,424 |
| Cow | 5 | 117,624,064 | 26.1 | 34,557 | 8,576 | 43.7 | 42 | 3,074 | 90 | 3,389,312 |
| Cow | 6 | 113,387,872 | 16.1 | 54,888 | 9,104 | 41.5 | 41 | 1,820 | 88 | 4,395,744 |
| Cow | 7 | 106,864,480 | 31 | 29,098 | 8,592 | 44 | 43 | 3,318 | 90 | 2,089,536 |
| Cow | 8 | 108,989,536 | 22.5 | 35,370 | 9,120 | 42.3 | 42 | 2,456 | 80 | 2,730,176 |
| Cow | 9 | 102,186,560 | 16.2 | 49,140 | 9,248 | 39.9 | 40 | 1,654 | 80 | 2,480,832 |
| Cow | 10 | 100,506,688 | 24.9 | 32,930 | 9,248 | 42.3 | 42 | 2,499 | 82 | 2,266,048 |
| Cow | 11 | 103,892,640 | 37.3 | 24,138 | 8,320 | 44.4 | 43 | 3,873 | 90 | 1,432,416 |
| Cow | 12 | 80,766,656 | 15.4 | 53,977 | 9,728 | 41.4 | 41 | 1,247 | 83 | 5,460,160 |
| Cow | 13 | 79,882,464 | 32.5 | 25,123 | 8,544 | 44.6 | 44 | 2,597 | 82 | 1,232,384 |
| Cow | 16 | 72,475,040 | 35.2 | 25,373 | 8,512 | 44.2 | 43 | 2,551 | 89 | 1,746,240 |
| Cow | 17 | 71,031,328 | 26.6 | 34,462 | 8,992 | 45 | 44 | 1,891 | 92 | 5,221,472 |
| Cow | 18 | 61,271,040 | 47.6 | 18,645 | 8,000 | 46.9 | 46 | 2,918 | 89 | 1,108,608 |
| Cow | 19 | 60,848,224 | 49.2 | 18,158 | 7,776 | 47.3 | 47 | 2,992 | 89 | 1,227,104 |
| Cow | 20 | 70,909,696 | 19.5 | 44,130 | 9,008 | 42.6 | 42 | 1,380 | 86 | 6,105,280 |
| Cow | 21 | 65,176,704 | 34.2 | 25,257 | 7,808 | 44.4 | 43 | 2,227 | 86 | 3,427,520 |
| Cow | 22 | 58,557,152 | 31.9 | 27,326 | 8,576 | 45 | 44 | 1,866 | 87 | 1,689,376 |
| Cow | 23 | 49,505,216 | 27.1 | 29,874 | 8,864 | 44.4 | 43 | 1,342 | 81 | 1,460,832 |
| Cow | 24 | 61,194,496 | 19.8 | 38,840 | 8,576 | 42.4 | 42 | 1,213 | 77 | 1,703,040 |
| Cow | 25 | 41,451,456 | 47.4 | 18,164 | 7,744 | 47.5 | 46 | 1,963 | 86 | 840,096 |
| Cow | 26 | 48,502,016 | 33.2 | 26,406 | 8,160 | 44 | 43 | 1,609 | 88 | 1,410,816 |
| Cow | 27 | 45,288,160 | 19.1 | 39,012 | 8,800 | 42.2 | 42 | 866 | 75 | 2,678,784 |
| Cow | 28 | 43,186,240 | 29.2 | 29,025 | 8,240 | 43.9 | 43 | 1,260 | 85 | 2,173,536 |
| Cow | 29 | 48,141,824 | 41.3 | 22,402 | 7,552 | 46.5 | 45 | 1,987 | 92 | 1,552,288 |
| Cow | X | 83,036,672 | 25.6 | 29,711 | 8,512 | 41.7 | 41 | 2,125 | 76 | 2,017,728 |
| Cow | Y | 38,719,968 | 40 | 17,935 | 9,504 | 43.6 | 42 | 1,547 | 72 | 444,384 |
| Opossum | 1 | 733,412,928 | 17 | 45,145 | 9,296 | 38.4 | 37 | 12,440 | 77 | 4,972,672 |
| Opossum | 2 | 527,981,664 | 19.9 | 41,721 | 8,864 | 39.2 | 38 | 10,520 | 83 | 7,789,664 |
| Opossum | 3 | 515,087,168 | 17.3 | 47,199 | 9,248 | 38 | 37 | 8,916 | 82 | 6,875,168 |
| Opossum | 4 | 422,697,408 | 17.6 | 45,407 | 8,992 | 38.6 | 37 | 7,451 | 80 | 5,873,632 |
| Opossum | 5 | 298,042,848 | 17.7 | 44,589 | 9,568 | 37.6 | 36 | 5,288 | 79 | 3,080,672 |
| Opossum | 6 | 284,524,512 | 19.8 | 39,704 | 8,928 | 38.7 | 38 | 5,643 | 79 | 6,446,304 |
| Opossum | 7 | 254,715,584 | 13 | 61,026 | 9,312 | 37.8 | 37 | 3,320 | 80 | 10,514,048 |
| Opossum | 8 | 303,158,720 | 21.6 | 39,084 | 8,672 | 39.2 | 38 | 6,550 | 84 | 5,223,456 |
| Opossum | X | 72,972,128 | 44.7 | 18,463 | 7,232 | 42.3 | 42 | 3,265 | 83 | 1,003,072 |
| Chicken | 1 | 195,191,328 | 25.9 | 31,956 | 10,720 | 40.2 | 39 | 5,046 | 83 | 2,855,424 |
| Chicken | 2 | 150,357,856 | 24.8 | 32,216 | 11,456 | 39.4 | 39 | 3,728 | 80 | 1,428,256 |
| Chicken | 3 | 110,204,896 | 26.2 | 31,129 | 11,680 | 39.9 | 39 | 2,886 | 82 | 1,768,480 |
| Chicken | 4 | 90,936,160 | 25.7 | 33,135 | 11,872 | 40 | 39 | 2,341 | 85 | 4,008,128 |
| Chicken | 5 | 59,512,608 | 31.4 | 27,525 | 10,880 | 40.7 | 40 | 1,866 | 86 | 1,401,248 |
| Chicken | 6 | 35,175,936 | 29.6 | 26,811 | 12,160 | 41 | 40 | 1,041 | 79 | 521,312 |
| Chicken | 7 | 36,212,288 | 27.3 | 28,659 | 12,032 | 40.5 | 39 | 989 | 78 | 1,373,888 |
| Chicken | 8 | 28,570,208 | 31.3 | 27,934 | 10,240 | 42.2 | 41 | 894 | 87 | 1,584,928 |
| Chicken | 9 | 23,541,728 | 31.9 | 26,936 | 11,168 | 42.8 | 42 | 750 | 86 | 579,168 |
| Chicken | 10 | 20,484,704 | 35.6 | 23,087 | 11,072 | 42.4 | 41 | 730 | 82 | 493,312 |
| Chicken | 11 | 20,811,168 | 32.4 | 26,723 | 11,776 | 43.1 | 41 | 675 | 87 | 589,600 |
| Chicken | 12 | 19,573,280 | 31.1 | 26,873 | 11,552 | 43.3 | 42 | 608 | 83 | 706,336 |
| Chicken | 13 | 17,932,000 | 33.4 | 25,631 | 11,328 | 44.5 | 44 | 599 | 86 | 1,065,024 |
| Chicken | 14 | 15,306,464 | 39.5 | 22,308 | 10,992 | 45.4 | 44 | 604 | 88 | 587,200 |
| Chicken | 15 | 12,585,504 | 31.8 | 26,161 | 11,488 | 45.6 | 44 | 400 | 83 | 809,216 |
| Chicken | 16 | 405,312 | 37 | 22,758 | 10,336 | 51.4 | 53 | 15 | 84 | 97,344 |
| Chicken | 17 | 10,337,984 | 40.1 | 21,036 | 10,144 | 46.6 | 46 | 415 | 84 | 324,448 |
| Chicken | 18 | 10,513,248 | 37.7 | 23,360 | 10,928 | 47.4 | 46 | 396 | 88 | 358,560 |
| Chicken | 19 | 9,570,432 | 40.4 | 19,953 | 10,560 | 46.6 | 45 | 387 | 81 | 266,208 |
| Chicken | 20 | 13,560,512 | 34.7 | 24,991 | 11,136 | 45.4 | 44 | 470 | 87 | 489,664 |
| Chicken | 21 | 6,656,928 | 35.9 | 20,951 | 11,936 | 46 | 45 | 239 | 75 | 300,928 |
| Chicken | 22 | 3,809,632 | 39.9 | 19,994 | 9,392 | 46 | 44 | 152 | 80 | 301,696 |
| Chicken | 23 | 5,248,000 | 46.7 | 17,567 | 9,216 | 49.1 | 48 | 245 | 82 | 272,256 |
| Chicken | 24 | 6,138,368 | 25.9 | 27,157 | 11,712 | 48.4 | 48 | 159 | 70 | 533,600 |
| Chicken | 25 | 1,367,840 | 66.5 | 12,645 | 7,936 | 53.5 | 54 | 91 | 84 | 89,568 |
| Chicken | 26 | 4,830,272 | 41.6 | 19,801 | 9,600 | 50.2 | 50 | 201 | 82 | 482,528 |
| Chicken | 27 | 4,538,432 | 43.2 | 18,550 | 8,400 | 49.3 | 48 | 196 | 80 | 649,984 |
| Chicken | 28 | 3,711,296 | 53.9 | 16,235 | 8,560 | 50.6 | 50 | 200 | 87 | 195,456 |
| Chicken | W | 233,824 | 17.1 | 17,096 | 6,560 | 41.5 | 41 | 4 | 29 | 51,456 |
| Chicken | Z | 67,535,552 | 26.9 | 29,416 | 9,952 | 40 | 39 | 1,814 | 79 | 1,181,600 |

**(c) Nonhomogeneous domains**

| **Species** | **Chr** | **Chromosome size** | **Density (per Mb)** | **Mean size (bp)** | **Median size (bp)** | **Mean GC%** | **Median GC%** | **#Domains** | **Genome coverage (%)** | **Longest domain** |
| --- | --- | --- | --- | --- | --- | --- | --- | --- | --- | --- |
| Human | 1 | 217,916,736 | 10.1 | 16,801 | 6,784 | 44.1 | 44 | 2,191 | 17 | 734,336 |
| Human | 2 | 233,385,152 | 12.6 | 18,456 | 6,720 | 43.5 | 43 | 2,944 | 23 | 2,295,040 |
| Human | 3 | 194,309,312 | 11.5 | 18,455 | 7,008 | 42.6 | 42 | 2,243 | 21 | 1,507,296 |
| Human | 4 | 185,864,768 | 9.6 | 21,274 | 7,104 | 41.8 | 41 | 1,780 | 20 | 1,599,424 |
| Human | 5 | 174,600,768 | 12.1 | 15,743 | 6,688 | 42.9 | 42 | 2,108 | 19 | 695,392 |
| Human | 6 | 167,628,544 | 12.1 | 20,518 | 7,360 | 42.9 | 42 | 2,021 | 25 | 1,217,568 |
| Human | 7 | 151,582,880 | 11.8 | 15,525 | 6,624 | 44.9 | 44 | 1,790 | 18 | 497,824 |
| Human | 8 | 140,746,368 | 11.5 | 17,180 | 6,560 | 43.2 | 43 | 1,615 | 20 | 1,181,984 |
| Human | 9 | 109,434,656 | 11.9 | 15,242 | 6,656 | 44.6 | 44 | 1,303 | 18 | 705,824 |
| Human | 10 | 128,196,704 | 13.6 | 16,849 | 6,560 | 44 | 43 | 1,742 | 23 | 854,528 |
| Human | 11 | 130,274,560 | 9.8 | 13,752 | 6,176 | 44.7 | 44 | 1,278 | 13 | 462,848 |
| Human | 12 | 129,843,424 | 10.5 | 18,391 | 6,784 | 44.1 | 44 | 1,361 | 19 | 923,904 |
| Human | 13 | 94,957,600 | 11 | 21,949 | 7,008 | 42.4 | 42 | 1,045 | 24 | 1,370,112 |
| Human | 14 | 86,964,576 | 10.4 | 16,965 | 7,200 | 43.8 | 43 | 902 | 18 | 304,512 |
| Human | 15 | 77,770,144 | 16.2 | 16,800 | 7,408 | 43.7 | 43 | 1,256 | 27 | 759,936 |
| Human | 16 | 74,960,800 | 11.4 | 15,830 | 6,336 | 46.3 | 46 | 856 | 18 | 773,120 |
| Human | 17 | 75,513,088 | 12.3 | 12,674 | 6,560 | 45.6 | 45 | 928 | 16 | 204,448 |
| Human | 18 | 74,291,328 | 13.7 | 19,570 | 6,960 | 42.7 | 42 | 1,016 | 27 | 578,144 |
| Human | 19 | 54,533,856 | 15.9 | 9,614 | 6,464 | 47 | 47 | 867 | 15 | 128,832 |
| Human | 20 | 59,266,464 | 12.9 | 15,121 | 6,368 | 45.4 | 45 | 763 | 19 | 383,936 |
| Human | 21 | 33,211,648 | 10.8 | 11,533 | 6,144 | 44.6 | 44 | 360 | 13 | 289,856 |
| Human | 22 | 33,725,632 | 16.6 | 11,779 | 6,208 | 45.9 | 46 | 560 | 20 | 419,264 |
| Human | X | 135,172,992 | 12.5 | 21,028 | 7,520 | 42.4 | 42 | 1,696 | 26 | 1,701,696 |
| Human | Y | 18,186,240 | 20.2 | 17,695 | 7,168 | 39.4 | 38 | 367 | 36 | 674,656 |
| Chimpanzee | 1 | 217,189,792 | 11 | 15,752 | 6,688 | 43.9 | 43 | 2,388 | 17 | 605,856 |
| Chimpanzee | 2A | 105,873,440 | 15.2 | 17,560 | 6,720 | 43.6 | 43 | 1,614 | 27 | 1,794,688 |
| Chimpanzee | 2B | 127,869,088 | 12.6 | 15,858 | 6,784 | 43.5 | 43 | 1,615 | 20 | 929,856 |
| Chimpanzee | 3 | 194,962,656 | 12 | 17,240 | 7,072 | 42.8 | 42 | 2,349 | 21 | 439,424 |
| Chimpanzee | 4 | 186,955,680 | 10.9 | 20,563 | 7,136 | 42 | 41 | 2,037 | 22 | 1,156,640 |
| Chimpanzee | 5 | 175,225,344 | 12.6 | 15,917 | 6,880 | 42.9 | 42 | 2,211 | 20 | 801,600 |
| Chimpanzee | 6 | 164,697,664 | 11.5 | 22,174 | 7,232 | 43 | 42 | 1,889 | 25 | 1,912,032 |
| Chimpanzee | 7 | 151,069,600 | 12.2 | 16,223 | 6,688 | 44.3 | 44 | 1,841 | 20 | 1,186,848 |
| Chimpanzee | 8 | 138,150,976 | 12.5 | 17,263 | 6,656 | 42.9 | 42 | 1,730 | 22 | 1,167,616 |
| Chimpanzee | 9 | 109,295,936 | 11.7 | 14,204 | 6,432 | 45 | 45 | 1,279 | 17 | 555,360 |
| Chimpanzee | 10 | 125,696,576 | 13.1 | 17,844 | 6,624 | 44.1 | 43 | 1,647 | 23 | 1,435,424 |
| Chimpanzee | 11 | 123,596,640 | 11.1 | 12,935 | 6,128 | 44.8 | 44 | 1,376 | 14 | 613,824 |
| Chimpanzee | 12 | 129,867,936 | 11.1 | 18,801 | 6,992 | 44.1 | 44 | 1,444 | 21 | 553,568 |
| Chimpanzee | 13 | 87,794,784 | 11.3 | 20,246 | 7,040 | 42.6 | 42 | 996 | 23 | 1,393,056 |
| Chimpanzee | 14 | 86,251,392 | 11.5 | 17,021 | 6,656 | 44.3 | 43 | 990 | 20 | 355,232 |
| Chimpanzee | 15 | 76,971,936 | 16.6 | 15,237 | 7,168 | 44 | 43 | 1,277 | 25 | 378,208 |
| Chimpanzee | 16 | 74,505,664 | 12.5 | 14,445 | 6,480 | 46.2 | 46 | 930 | 18 | 768,064 |
| Chimpanzee | 17 | 73,429,760 | 12.8 | 13,250 | 6,496 | 46 | 45 | 943 | 17 | 459,648 |
| Chimpanzee | 18 | 74,181,056 | 13.4 | 20,528 | 6,912 | 42.8 | 42 | 997 | 28 | 762,784 |
| Chimpanzee | 19 | 51,997,056 | 15.8 | 10,522 | 6,528 | 47.3 | 47 | 822 | 17 | 300,928 |
| Chimpanzee | 20 | 58,102,080 | 13.6 | 14,483 | 6,496 | 45.4 | 45 | 793 | 20 | 390,304 |
| Chimpanzee | 21 | 32,706,016 | 9.3 | 12,548 | 5,824 | 45.1 | 44 | 303 | 12 | 301,504 |
| Chimpanzee | 22 | 32,341,152 | 16.7 | 10,399 | 6,240 | 45.9 | 46 | 541 | 17 | 159,456 |
| Chimpanzee | X | 130,929,824 | 12.2 | 21,160 | 7,616 | 42.3 | 42 | 1,599 | 26 | 839,040 |
| Chimpanzee | Y | 22,691,168 | 25.4 | 15,633 | 7,440 | 40.2 | 39 | 576 | 40 | 477,312 |
| Orangutan | 1 | 216,060,736 | 11.5 | 15,470 | 7,072 | 44 | 43 | 2,489 | 18 | 598,496 |
| Orangutan | 2A | 104,616,320 | 14.9 | 17,861 | 7,072 | 43.3 | 43 | 1,557 | 27 | 1,399,136 |
| Orangutan | 2B | 148,146,336 | 11.6 | 21,114 | 7,584 | 42.5 | 42 | 1,715 | 24 | 1,329,248 |
| Orangutan | 3 | 190,355,264 | 11.5 | 18,522 | 7,200 | 42.7 | 42 | 2,190 | 21 | 798,048 |
| Orangutan | 4 | 186,135,648 | 11.1 | 18,615 | 7,168 | 42 | 41 | 2,073 | 21 | 1,297,888 |
| Orangutan | 5 | 172,726,976 | 11.7 | 17,721 | 6,992 | 42.7 | 42 | 2,016 | 21 | 1,528,576 |
| Orangutan | 6 | 164,088,672 | 11.1 | 24,014 | 7,584 | 42.9 | 43 | 1,829 | 27 | 2,108,448 |
| Orangutan | 7 | 145,686,624 | 12.7 | 14,632 | 6,848 | 44.6 | 44 | 1,845 | 19 | 440,224 |
| Orangutan | 8 | 140,594,368 | 12.1 | 16,378 | 6,752 | 43.2 | 43 | 1,697 | 20 | 825,664 |
| Orangutan | 9 | 109,204,832 | 12.6 | 14,567 | 6,656 | 44.4 | 44 | 1,378 | 18 | 594,272 |
| Orangutan | 10 | 124,371,648 | 13.1 | 16,360 | 6,784 | 44.2 | 44 | 1,627 | 21 | 1,066,464 |
| Orangutan | 11 | 124,382,944 | 11.3 | 12,547 | 6,144 | 44.4 | 44 | 1,408 | 14 | 344,704 |
| Orangutan | 12 | 128,777,280 | 11 | 18,493 | 6,832 | 43.9 | 44 | 1,412 | 20 | 835,584 |
| Orangutan | 13 | 94,686,048 | 10.1 | 22,063 | 7,312 | 42.4 | 42 | 960 | 22 | 1,473,696 |
| Orangutan | 14 | 86,577,920 | 10.6 | 16,829 | 6,992 | 43.9 | 43 | 914 | 18 | 325,152 |
| Orangutan | 15 | 75,961,056 | 17.3 | 16,350 | 7,200 | 43.7 | 43 | 1,313 | 28 | 702,432 |
| Orangutan | 16 | 70,802,048 | 12.3 | 14,050 | 6,256 | 46.5 | 46 | 868 | 17 | 437,600 |
| Orangutan | 17 | 67,081,376 | 12.7 | 13,264 | 6,496 | 46 | 45 | 851 | 17 | 240,768 |
| Orangutan | 18 | 73,515,712 | 13.6 | 20,347 | 7,104 | 42.6 | 42 | 1,001 | 28 | 650,752 |
| Orangutan | 19 | 51,367,232 | 15.4 | 10,249 | 6,240 | 46.8 | 47 | 789 | 16 | 200,832 |
| Orangutan | 20 | 58,061,408 | 14.3 | 14,080 | 6,656 | 45.5 | 45 | 831 | 20 | 373,792 |
| Orangutan | 21 | 33,052,576 | 9.8 | 14,186 | 6,080 | 45 | 44 | 323 | 14 | 315,360 |
| Orangutan | 22 | 30,217,376 | 17.3 | 10,681 | 6,288 | 45.9 | 46 | 522 | 18 | 310,240 |
| Orangutan | X | 126,496,928 | 13.4 | 15,372 | 6,944 | 43.5 | 43 | 1,696 | 21 | 511,744 |
| Mouse | 1 | 191,477,376 | 9.2 | 22,681 | 7,680 | 41.8 | 42 | 1,767 | 21 | 1,505,760 |
| Mouse | 2 | 178,392,032 | 9.7 | 16,195 | 7,328 | 42.7 | 43 | 1,739 | 16 | 627,072 |
| Mouse | 3 | 156,393,856 | 10.3 | 23,547 | 7,936 | 41.3 | 41 | 1,606 | 24 | 826,176 |
| Mouse | 4 | 151,886,784 | 8.9 | 17,370 | 6,976 | 43.3 | 43 | 1,356 | 16 | 888,640 |
| Mouse | 5 | 147,721,152 | 7.7 | 15,744 | 7,136 | 43.7 | 44 | 1,140 | 12 | 733,120 |
| Mouse | 6 | 146,316,992 | 8.5 | 22,148 | 7,616 | 42 | 42 | 1,247 | 19 | 1,016,992 |
| Mouse | 7 | 141,878,176 | 12.3 | 16,285 | 7,424 | 44 | 44 | 1,745 | 20 | 508,192 |
| Mouse | 8 | 124,796,736 | 7.8 | 19,146 | 6,656 | 42.7 | 43 | 976 | 15 | 686,112 |
| Mouse | 9 | 120,720,160 | 12.1 | 19,892 | 7,424 | 43 | 43 | 1,462 | 24 | 755,968 |
| Mouse | 10 | 126,847,808 | 9.1 | 19,970 | 7,456 | 42 | 42 | 1,160 | 18 | 630,016 |
| Mouse | 11 | 118,743,520 | 11.7 | 18,466 | 7,328 | 44.3 | 44 | 1,386 | 22 | 774,912 |
| Mouse | 12 | 117,459,264 | 9.9 | 20,439 | 7,680 | 42.1 | 42 | 1,161 | 20 | 1,612,320 |
| Mouse | 13 | 116,370,848 | 11.3 | 25,727 | 7,872 | 41.8 | 41 | 1,319 | 29 | 2,748,672 |
| Mouse | 14 | 121,635,264 | 9.5 | 32,652 | 7,520 | 41.5 | 41 | 1,153 | 31 | 7,258,464 |
| Mouse | 15 | 100,439,936 | 9.4 | 16,059 | 7,040 | 42.7 | 43 | 941 | 15 | 722,976 |
| Mouse | 16 | 95,004,896 | 9 | 21,438 | 7,328 | 41.6 | 41 | 858 | 19 | 1,705,920 |
| Mouse | 17 | 91,898,144 | 8.3 | 15,938 | 6,720 | 43.5 | 43 | 760 | 13 | 646,304 |
| Mouse | 18 | 87,600,032 | 11 | 24,085 | 8,016 | 42 | 42 | 964 | 27 | 642,112 |
| Mouse | 19 | 58,142,176 | 11.5 | 20,426 | 7,200 | 43.8 | 43 | 668 | 23 | 1,168,640 |
| Mouse | X | 162,080,832 | 12.3 | 32,903 | 7,776 | 39.7 | 39 | 1,993 | 40 | 2,662,816 |
| Mouse | Y | 2,702,496 | 14.4 | 37,834 | 8,384 | 36 | 36 | 39 | 55 | 487,904 |
| Rat | 1 | 242,568,192 | 10.6 | 17,685 | 7,424 | 43.6 | 43 | 2,563 | 19 | 556,224 |
| Rat | 2 | 235,352,352 | 9.8 | 27,508 | 7,936 | 41.5 | 42 | 2,313 | 27 | 3,260,224 |
| Rat | 3 | 157,225,152 | 9.1 | 15,234 | 6,816 | 43.3 | 43 | 1,427 | 14 | 671,776 |
| Rat | 4 | 172,528,416 | 9.4 | 18,561 | 7,424 | 42.7 | 43 | 1,620 | 17 | 871,072 |
| Rat | 5 | 157,560,736 | 7.5 | 18,925 | 7,008 | 43.8 | 44 | 1,180 | 14 | 380,960 |
| Rat | 6 | 134,552,608 | 9 | 20,752 | 7,328 | 42.9 | 43 | 1,206 | 19 | 988,000 |
| Rat | 7 | 131,647,424 | 9.1 | 15,374 | 6,944 | 43.7 | 44 | 1,199 | 14 | 626,304 |
| Rat | 8 | 118,069,792 | 11.4 | 21,108 | 7,552 | 43.1 | 43 | 1,347 | 24 | 495,936 |
| Rat | 9 | 104,712,416 | 8.8 | 19,622 | 7,456 | 42.3 | 42 | 923 | 17 | 712,512 |
| Rat | 10 | 101,026,080 | 13.4 | 18,411 | 7,872 | 45.1 | 45 | 1,353 | 25 | 625,536 |
| Rat | 11 | 81,764,224 | 8.2 | 17,654 | 7,008 | 42.3 | 43 | 671 | 14 | 401,568 |
| Rat | 12 | 41,216,160 | 13.6 | 24,681 | 8,608 | 46.5 | 46 | 561 | 34 | 859,552 |
| Rat | 13 | 102,334,624 | 9.5 | 21,359 | 7,072 | 41.8 | 42 | 968 | 20 | 1,605,696 |
| Rat | 14 | 100,801,632 | 9.6 | 20,314 | 7,328 | 42.2 | 42 | 970 | 20 | 1,014,240 |
| Rat | 15 | 98,895,328 | 9.5 | 30,463 | 7,840 | 41.9 | 42 | 939 | 29 | 3,434,784 |
| Rat | 16 | 81,355,456 | 9.4 | 17,724 | 7,072 | 42.6 | 42 | 763 | 17 | 863,392 |
| Rat | 17 | 87,674,496 | 10 | 24,725 | 8,000 | 43 | 43 | 877 | 25 | 3,432,896 |
| Rat | 18 | 79,668,352 | 10.8 | 21,980 | 7,248 | 42.2 | 42 | 864 | 24 | 472,384 |
| Rat | 19 | 53,394,240 | 9.8 | 19,408 | 6,848 | 44.3 | 44 | 525 | 19 | 906,656 |
| Rat | 20 | 49,307,744 | 6.6 | 15,036 | 7,680 | 44.3 | 45 | 324 | 10 | 166,560 |
| Rat | X | 145,397,312 | 11 | 36,181 | 8,032 | 39.8 | 39 | 1,599 | 40 | 7,472,416 |
| Horse | 1 | 183,561,792 | 13.8 | 10,525 | 6,016 | 45.2 | 45 | 2,536 | 15 | 307,424 |
| Horse | 2 | 118,957,504 | 7.3 | 8,451 | 5,504 | 46.4 | 46 | 863 | 6 | 262,208 |
| Horse | 3 | 118,104,864 | 8.9 | 10,156 | 5,712 | 46.4 | 46 | 1,046 | 9 | 180,352 |
| Horse | 4 | 107,397,024 | 15.2 | 12,186 | 6,240 | 43.9 | 43 | 1,632 | 19 | 287,040 |
| Horse | 5 | 97,742,496 | 14.9 | 11,539 | 6,176 | 43.8 | 44 | 1,457 | 17 | 529,568 |
| Horse | 6 | 83,857,984 | 11.6 | 9,614 | 6,304 | 45.3 | 45 | 976 | 11 | 98,656 |
| Horse | 7 | 96,472,384 | 8.8 | 9,770 | 5,728 | 47.4 | 47 | 847 | 9 | 251,872 |
| Horse | 8 | 92,896,064 | 10.4 | 11,228 | 6,448 | 46.8 | 47 | 966 | 12 | 292,896 |
| Horse | 9 | 82,750,176 | 12.4 | 11,765 | 6,272 | 43.9 | 43 | 1,022 | 15 | 171,808 |
| Horse | 10 | 82,681,984 | 11.2 | 10,266 | 6,144 | 46.7 | 46 | 928 | 12 | 187,552 |
| Horse | 11 | 60,492,896 | 9.4 | 9,056 | 5,456 | 46.6 | 46 | 570 | 9 | 387,680 |
| Horse | 12 | 31,940,768 | 5.1 | 6,320 | 4,960 | 47.9 | 48 | 164 | 3 | 39,776 |
| Horse | 13 | 41,521,568 | 12.1 | 8,429 | 5,952 | 48 | 48 | 503 | 10 | 63,168 |
| Horse | 14 | 92,877,376 | 12.9 | 11,247 | 6,176 | 44.9 | 45 | 1,197 | 14 | 221,664 |
| Horse | 15 | 90,790,016 | 16.8 | 11,393 | 6,240 | 44.9 | 44 | 1,523 | 19 | 226,976 |
| Horse | 16 | 86,752,160 | 13.3 | 13,107 | 6,592 | 44.9 | 44 | 1,150 | 17 | 470,368 |
| Horse | 17 | 80,151,872 | 13.8 | 14,552 | 6,816 | 43.7 | 43 | 1,109 | 20 | 282,976 |
| Horse | 18 | 81,597,248 | 14.9 | 11,784 | 6,624 | 43.8 | 43 | 1,214 | 18 | 200,000 |
| Horse | 19 | 59,453,792 | 19.1 | 14,934 | 6,688 | 42.1 | 42 | 1,135 | 29 | 413,824 |
| Horse | 20 | 63,448,096 | 14.2 | 12,443 | 6,176 | 44.9 | 44 | 903 | 18 | 382,880 |
| Horse | 21 | 57,021,056 | 10.3 | 10,934 | 6,160 | 45.6 | 45 | 590 | 11 | 290,304 |
| Horse | 22 | 49,278,304 | 13.5 | 8,291 | 5,696 | 46.8 | 46 | 667 | 11 | 67,008 |
| Horse | 23 | 55,051,296 | 15.9 | 14,112 | 6,432 | 44.1 | 43 | 877 | 22 | 879,968 |
| Horse | 24 | 45,792,672 | 11.2 | 8,768 | 5,728 | 46.2 | 46 | 513 | 10 | 170,784 |
| Horse | 25 | 38,841,888 | 8.2 | 7,723 | 5,456 | 47.7 | 48 | 318 | 6 | 54,144 |
| Horse | 26 | 41,476,928 | 9.5 | 12,904 | 6,384 | 46.7 | 46 | 396 | 12 | 270,208 |
| Horse | 27 | 39,469,792 | 16.6 | 14,966 | 7,040 | 43.4 | 43 | 657 | 25 | 556,256 |
| Horse | 28 | 45,531,360 | 6.7 | 9,168 | 5,728 | 46.4 | 46 | 304 | 6 | 230,048 |
| Horse | 29 | 33,264,096 | 20.4 | 13,987 | 6,688 | 44 | 44 | 678 | 29 | 214,528 |
| Horse | 30 | 29,835,520 | 11.2 | 10,484 | 6,480 | 45.5 | 45 | 334 | 12 | 161,376 |
| Horse | 31 | 24,827,520 | 16.1 | 13,695 | 6,144 | 44.4 | 44 | 399 | 22 | 542,080 |
| Horse | X | 121,614,432 | 14 | 13,665 | 6,560 | 42.8 | 42 | 1,707 | 19 | 1,244,672 |
| Dog | 1 | 122,070,560 | 16.2 | 13,007 | 6,400 | 46.4 | 46 | 1,982 | 21 | 811,296 |
| Dog | 2 | 84,986,272 | 13.3 | 13,747 | 6,432 | 47.2 | 46 | 1,128 | 18 | 325,184 |
| Dog | 3 | 91,547,360 | 13.8 | 14,547 | 6,336 | 45 | 44 | 1,264 | 20 | 637,696 |
| Dog | 4 | 88,054,560 | 12.5 | 13,739 | 6,528 | 45.5 | 44 | 1,099 | 17 | 546,496 |
| Dog | 5 | 88,556,640 | 14.2 | 13,124 | 6,400 | 46.1 | 45 | 1,259 | 19 | 667,808 |
| Dog | 6 | 77,066,624 | 12.1 | 12,810 | 6,528 | 46.4 | 46 | 929 | 15 | 618,688 |
| Dog | 7 | 80,608,000 | 13.3 | 13,560 | 6,464 | 45.5 | 45 | 1,071 | 18 | 387,264 |
| Dog | 8 | 74,071,520 | 11.5 | 12,064 | 6,608 | 45.8 | 44 | 854 | 14 | 272,192 |
| Dog | 9 | 60,688,608 | 11.8 | 12,544 | 6,624 | 46.6 | 45 | 716 | 15 | 296,864 |
| Dog | 10 | 68,960,640 | 13.8 | 11,667 | 6,160 | 47.8 | 47 | 950 | 16 | 212,768 |
| Dog | 11 | 74,159,296 | 13.7 | 16,066 | 6,752 | 45 | 44 | 1,019 | 22 | 458,528 |
| Dog | 12 | 72,323,808 | 12.6 | 15,712 | 6,960 | 44.7 | 43 | 914 | 20 | 393,120 |
| Dog | 13 | 62,975,520 | 10.6 | 10,479 | 6,240 | 46.8 | 46 | 669 | 11 | 259,552 |
| Dog | 14 | 60,715,328 | 14.1 | 16,417 | 6,816 | 43.7 | 42 | 857 | 23 | 1,834,912 |
| Dog | 15 | 63,855,456 | 14.6 | 12,880 | 6,640 | 46.3 | 45 | 932 | 19 | 246,528 |
| Dog | 16 | 59,207,872 | 15.5 | 13,143 | 6,432 | 46.2 | 45 | 920 | 20 | 223,968 |
| Dog | 17 | 64,090,304 | 15.4 | 13,856 | 6,528 | 45.9 | 45 | 989 | 21 | 265,792 |
| Dog | 18 | 55,531,008 | 10.8 | 10,420 | 6,080 | 47.7 | 47 | 602 | 11 | 264,320 |
| Dog | 19 | 53,607,136 | 16.9 | 12,277 | 6,704 | 43.6 | 43 | 904 | 21 | 302,944 |
| Dog | 20 | 57,839,648 | 10.5 | 12,923 | 6,720 | 47.2 | 46 | 609 | 14 | 350,336 |
| Dog | 21 | 50,623,104 | 13.3 | 14,291 | 6,304 | 43.7 | 43 | 673 | 19 | 214,816 |
| Dog | 22 | 61,283,552 | 13 | 13,504 | 6,656 | 45.3 | 44 | 799 | 18 | 409,344 |
| Dog | 23 | 52,169,408 | 16.6 | 16,622 | 6,864 | 43.9 | 43 | 868 | 28 | 1,281,376 |
| Dog | 24 | 47,496,224 | 12.7 | 13,847 | 6,288 | 46.9 | 45 | 602 | 18 | 845,792 |
| Dog | 25 | 51,352,384 | 11.8 | 12,372 | 6,112 | 46.8 | 46 | 606 | 15 | 349,216 |
| Dog | 26 | 38,804,896 | 13.8 | 15,759 | 6,432 | 46.1 | 45 | 537 | 22 | 418,272 |
| Dog | 27 | 45,725,984 | 13.5 | 13,780 | 6,400 | 44.6 | 43 | 617 | 19 | 398,720 |
| Dog | 28 | 40,985,856 | 13.9 | 13,243 | 6,128 | 47.6 | 46 | 568 | 18 | 711,776 |
| Dog | 29 | 41,709,440 | 14.6 | 15,240 | 7,152 | 43.3 | 42 | 610 | 22 | 491,296 |
| Dog | 30 | 40,042,528 | 14.6 | 13,506 | 7,232 | 45.3 | 44 | 586 | 20 | 281,728 |
| Dog | 31 | 39,763,008 | 10.5 | 10,750 | 6,320 | 48.5 | 48 | 418 | 11 | 180,064 |
| Dog | 32 | 38,755,040 | 12.8 | 29,587 | 7,728 | 40.5 | 39 | 498 | 38 | 1,387,488 |
| Dog | 33 | 31,238,496 | 16.3 | 15,416 | 6,912 | 45.2 | 44 | 509 | 25 | 456,608 |
| Dog | 34 | 41,922,592 | 17.1 | 12,831 | 6,240 | 45.7 | 45 | 716 | 22 | 484,352 |
| Dog | 35 | 26,416,608 | 19.6 | 17,984 | 6,496 | 45.3 | 44 | 517 | 35 | 2,211,808 |
| Dog | 36 | 30,684,608 | 18.9 | 17,156 | 7,008 | 43.9 | 43 | 581 | 32 | 803,872 |
| Dog | 37 | 30,741,600 | 12.8 | 16,068 | 6,432 | 46.3 | 45 | 392 | 20 | 740,480 |
| Dog | 38 | 23,779,040 | 11 | 10,353 | 6,272 | 46.4 | 46 | 262 | 11 | 87,136 |
| Dog | X | 123,181,696 | 13.9 | 16,057 | 6,784 | 44.2 | 43 | 1,718 | 22 | 593,696 |
| Pig | 1 | 291,977,152 | 12 | 14,192 | 6,528 | 43.9 | 43 | 3,493 | 17 | 498,624 |
| Pig | 2 | 138,150,912 | 8.1 | 8,870 | 5,952 | 45.7 | 45 | 1,117 | 7 | 221,504 |
| Pig | 3 | 121,501,024 | 10.8 | 10,040 | 5,984 | 46.3 | 46 | 1,314 | 11 | 240,224 |
| Pig | 4 | 134,999,424 | 11.6 | 14,609 | 6,528 | 44.5 | 44 | 1,566 | 17 | 409,152 |
| Pig | 5 | 98,837,824 | 9.6 | 10,032 | 6,064 | 45.7 | 45 | 952 | 10 | 136,096 |
| Pig | 6 | 121,210,944 | 8.8 | 8,345 | 5,760 | 46.3 | 46 | 1,063 | 7 | 119,264 |
| Pig | 7 | 135,052,864 | 11.2 | 11,548 | 6,400 | 45.9 | 45 | 1,510 | 13 | 411,840 |
| Pig | 8 | 117,775,104 | 11.2 | 16,313 | 7,104 | 44.3 | 43 | 1,321 | 18 | 773,888 |
| Pig | 9 | 130,657,248 | 11.7 | 11,956 | 6,336 | 44.3 | 44 | 1,527 | 14 | 630,304 |
| Pig | 10 | 65,775,680 | 15.1 | 14,190 | 7,008 | 44.9 | 44 | 993 | 21 | 297,536 |
| Pig | 11 | 78,558,880 | 10.2 | 14,672 | 6,272 | 46.4 | 46 | 799 | 15 | 798,528 |
| Pig | 12 | 56,502,336 | 10.4 | 8,462 | 5,856 | 47.6 | 47 | 590 | 9 | 59,520 |
| Pig | 13 | 142,767,264 | 12.6 | 15,721 | 7,040 | 42.9 | 42 | 1,793 | 20 | 1,250,048 |
| Pig | 14 | 147,417,504 | 12.2 | 11,554 | 6,240 | 44.8 | 44 | 1,792 | 14 | 321,344 |
| Pig | 15 | 132,148,704 | 11.9 | 14,413 | 6,912 | 44.2 | 44 | 1,571 | 17 | 577,504 |
| Pig | 16 | 76,457,888 | 13 | 13,700 | 6,656 | 44.4 | 44 | 991 | 18 | 404,384 |
| Pig | 17 | 63,427,904 | 13.4 | 11,839 | 6,464 | 45.5 | 45 | 851 | 16 | 893,312 |
| Pig | 18 | 53,590,720 | 11.8 | 12,882 | 6,432 | 45.9 | 46 | 632 | 15 | 201,952 |
| Pig | X | 124,471,488 | 13.5 | 14,364 | 6,848 | 44.4 | 44 | 1,685 | 19 | 737,632 |
| Cow | 1 | 151,811,296 | 11.4 | 14,873 | 6,528 | 42.5 | 42 | 1,726 | 17 | 579,264 |
| Cow | 2 | 135,113,632 | 9.2 | 11,524 | 6,240 | 43.8 | 43 | 1,247 | 11 | 335,520 |
| Cow | 3 | 119,266,624 | 10.1 | 11,293 | 6,128 | 44.7 | 44 | 1,202 | 11 | 423,936 |
| Cow | 4 | 117,710,720 | 11.4 | 12,974 | 6,176 | 43.8 | 43 | 1,346 | 15 | 1,798,592 |
| Cow | 5 | 117,624,064 | 9 | 10,760 | 6,112 | 45.2 | 45 | 1,059 | 10 | 187,456 |
| Cow | 6 | 113,387,872 | 8.7 | 13,600 | 6,656 | 44 | 43 | 992 | 12 | 531,424 |
| Cow | 7 | 106,864,480 | 8.8 | 10,976 | 5,952 | 45.6 | 45 | 940 | 10 | 519,328 |
| Cow | 8 | 108,989,536 | 14.4 | 14,117 | 6,400 | 43.1 | 43 | 1,567 | 20 | 742,048 |
| Cow | 9 | 102,186,560 | 11.6 | 17,704 | 6,752 | 42.4 | 42 | 1,181 | 20 | 1,967,584 |
| Cow | 10 | 100,506,688 | 13.6 | 13,295 | 6,816 | 43.8 | 43 | 1,370 | 18 | 391,456 |
| Cow | 11 | 103,892,640 | 10.6 | 9,435 | 5,792 | 46 | 46 | 1,103 | 10 | 213,856 |
| Cow | 12 | 80,766,656 | 9.6 | 17,365 | 6,720 | 44.7 | 44 | 775 | 17 | 1,031,072 |
| Cow | 13 | 79,882,464 | 14 | 13,047 | 6,112 | 45.5 | 45 | 1,122 | 18 | 746,976 |
| Cow | 16 | 72,475,040 | 10.2 | 10,527 | 6,192 | 45 | 45 | 736 | 11 | 236,832 |
| Cow | 17 | 71,031,328 | 7.4 | 11,104 | 6,352 | 47.3 | 47 | 528 | 8 | 98,048 |
| Cow | 18 | 61,271,040 | 11.5 | 9,712 | 5,856 | 47.8 | 48 | 707 | 11 | 197,632 |
| Cow | 19 | 60,848,224 | 10.6 | 10,094 | 6,000 | 47.8 | 47 | 646 | 11 | 181,504 |
| Cow | 20 | 70,909,696 | 10.9 | 13,001 | 6,672 | 44.5 | 44 | 770 | 14 | 532,704 |
| Cow | 21 | 65,176,704 | 11.1 | 12,350 | 5,888 | 46 | 45 | 723 | 14 | 634,976 |
| Cow | 22 | 58,557,152 | 11.1 | 11,641 | 6,544 | 46.3 | 46 | 650 | 13 | 143,840 |
| Cow | 23 | 49,505,216 | 11.4 | 16,662 | 6,688 | 46.8 | 47 | 565 | 19 | 1,184,672 |
| Cow | 24 | 61,194,496 | 13.8 | 16,625 | 7,008 | 44.9 | 45 | 847 | 23 | 480,960 |
| Cow | 25 | 41,451,456 | 13 | 10,792 | 6,272 | 49.2 | 49 | 537 | 14 | 271,104 |
| Cow | 26 | 48,502,016 | 11.6 | 10,644 | 5,920 | 46 | 45 | 565 | 12 | 321,184 |
| Cow | 27 | 45,288,160 | 15.7 | 16,203 | 7,040 | 44.9 | 44 | 710 | 25 | 397,824 |
| Cow | 28 | 43,186,240 | 12.2 | 12,505 | 6,208 | 43.6 | 43 | 529 | 15 | 286,560 |
| Cow | 29 | 48,141,824 | 8.5 | 8,853 | 5,920 | 47.4 | 47 | 410 | 8 | 269,152 |
| Cow | X | 83,036,672 | 16.1 | 14,907 | 6,880 | 41.5 | 40 | 1,335 | 24 | 784,224 |
| Cow | Y | 38,719,968 | 19.8 | 14,347 | 9,120 | 40.5 | 39 | 765 | 28 | 204,480 |
| Opossum | 1 | 733,412,928 | 13.5 | 17,406 | 6,560 | 40.7 | 40 | 9,871 | 23 | 2,904,928 |
| Opossum | 2 | 527,981,664 | 12.3 | 13,695 | 6,368 | 42.1 | 41 | 6,504 | 17 | 958,080 |
| Opossum | 3 | 515,087,168 | 12.8 | 14,305 | 6,464 | 40.7 | 40 | 6,589 | 18 | 592,192 |
| Opossum | 4 | 422,697,408 | 13.5 | 14,820 | 6,528 | 40.9 | 40 | 5,693 | 20 | 1,173,920 |
| Opossum | 5 | 298,042,848 | 13.7 | 15,299 | 6,528 | 40 | 39 | 4,069 | 21 | 700,928 |
| Opossum | 6 | 284,524,512 | 13.2 | 16,135 | 6,576 | 41.3 | 40 | 3,748 | 21 | 1,718,656 |
| Opossum | 7 | 254,715,584 | 10.7 | 19,165 | 6,752 | 39.9 | 39 | 2,719 | 20 | 1,554,784 |
| Opossum | 8 | 303,158,720 | 12.1 | 12,815 | 6,160 | 42.1 | 41 | 3,680 | 16 | 707,136 |
| Opossum | X | 72,972,128 | 14.9 | 11,643 | 5,984 | 42.1 | 41 | 1,090 | 17 | 2,114,560 |
| Chicken | 1 | 195,191,328 | 11.7 | 14,926 | 6,368 | 44.2 | 44 | 2,274 | 17 | 1,243,648 |
| Chicken | 2 | 150,357,856 | 12.6 | 16,018 | 6,208 | 44.6 | 44 | 1,889 | 20 | 1,358,432 |
| Chicken | 3 | 110,204,896 | 11.6 | 15,910 | 6,528 | 45.8 | 45 | 1,280 | 18 | 1,301,984 |
| Chicken | 4 | 90,936,160 | 10 | 14,720 | 6,176 | 46.9 | 46 | 908 | 15 | 872,384 |
| Chicken | 5 | 59,512,608 | 10.4 | 13,190 | 6,432 | 48.5 | 48 | 618 | 14 | 460,576 |
| Chicken | 6 | 35,175,936 | 13.6 | 15,233 | 6,720 | 48.6 | 48 | 477 | 21 | 482,656 |
| Chicken | 7 | 36,212,288 | 11.5 | 18,870 | 6,496 | 47 | 46 | 417 | 22 | 1,799,808 |
| Chicken | 8 | 28,570,208 | 10.4 | 12,153 | 6,912 | 49.8 | 49 | 296 | 13 | 179,904 |
| Chicken | 9 | 23,541,728 | 9.9 | 14,333 | 6,976 | 50.2 | 49 | 233 | 14 | 151,392 |
| Chicken | 10 | 20,484,704 | 11.3 | 15,653 | 6,576 | 50.2 | 50 | 232 | 18 | 653,856 |
| Chicken | 11 | 20,811,168 | 10 | 13,333 | 6,944 | 49.4 | 48 | 208 | 13 | 222,880 |
| Chicken | 12 | 19,573,280 | 11.4 | 14,440 | 6,816 | 50.1 | 50 | 224 | 17 | 208,832 |
| Chicken | 13 | 17,932,000 | 10 | 14,326 | 7,104 | 51.1 | 51 | 180 | 14 | 174,304 |
| Chicken | 14 | 15,306,464 | 7.5 | 15,937 | 5,344 | 53.1 | 53 | 115 | 12 | 341,184 |
| Chicken | 15 | 12,585,504 | 10 | 16,834 | 6,960 | 52.5 | 52 | 126 | 17 | 288,896 |
| Chicken | 16 | 405,312 | 14.8 | 10,656 | 5,904 | 50.5 | 54 | 6 | 16 | 30,560 |
| Chicken | 17 | 10,337,984 | 11.7 | 13,290 | 8,064 | 51.9 | 51 | 121 | 16 | 177,184 |
| Chicken | 18 | 10,513,248 | 9.2 | 13,018 | 6,336 | 53.8 | 54 | 97 | 12 | 119,392 |
| Chicken | 19 | 9,570,432 | 11.7 | 16,504 | 6,160 | 53.1 | 53 | 112 | 19 | 306,368 |
| Chicken | 20 | 13,560,512 | 10.8 | 12,430 | 6,464 | 52.3 | 51 | 146 | 13 | 208,896 |
| Chicken | 21 | 6,656,928 | 15 | 16,496 | 6,624 | 52 | 51 | 100 | 25 | 247,488 |
| Chicken | 22 | 3,809,632 | 14.2 | 14,270 | 7,984 | 52.6 | 53 | 54 | 20 | 105,568 |
| Chicken | 23 | 5,248,000 | 15.1 | 11,950 | 6,464 | 53.9 | 54 | 79 | 18 | 72,032 |
| Chicken | 24 | 6,138,368 | 15.2 | 19,575 | 8,960 | 51.7 | 51 | 93 | 30 | 228,992 |
| Chicken | 25 | 1,367,840 | 15.4 | 10,342 | 6,848 | 54.1 | 55 | 21 | 16 | 45,984 |
| Chicken | 26 | 4,830,272 | 8.3 | 21,258 | 7,632 | 54 | 54 | 40 | 18 | 179,808 |
| Chicken | 27 | 4,538,432 | 11.2 | 17,700 | 8,128 | 53.7 | 53 | 51 | 20 | 336,256 |
| Chicken | 28 | 3,711,296 | 6.5 | 19,343 | 7,296 | 56.3 | 58 | 24 | 13 | 226,304 |
| Chicken | W | 233,824 | 25.7 | 27,573 | 13,616 | 40.9 | 43 | 6 | 71 | 107,136 |
| Chicken | Z | 67,535,552 | 13 | 16,090 | 6,400 | 44.2 | 44 | 881 | 21 | 1,215,264 |

(d) “Isochoric” domains

| **Species** | **Chr** | **Chromosome size** | **#Isochoric Domains** | **Isochoric Mean size** | **Isochoric Median size** | **Isochoric Mean GC %** | **Isochoric Median GC%** | **Isochoric Genome coverage (%)** |
| --- | --- | --- | --- | --- | --- | --- | --- | --- |
| Human | 1 | 217,916,736 | 82 | 598,505 | 423,088 | 40 | 39 | 23 |
| Human | 2 | 233,385,152 | 91 | 563,980 | 474,176 | 38 | 38 | 22 |
| Human | 3 | 194,309,312 | 88 | 663,570 | 508,192 | 38 | 38 | 30 |
| Human | 4 | 185,864,768 | 93 | 799,464 | 590,240 | 37 | 36 | 40 |
| Human | 5 | 174,600,768 | 71 | 720,378 | 506,112 | 38 | 38 | 29 |
| Human | 6 | 167,628,544 | 81 | 584,657 | 426,720 | 38 | 37 | 28 |
| Human | 7 | 151,582,880 | 64 | 700,760 | 512,512 | 38 | 37 | 30 |
| Human | 8 | 140,746,368 | 55 | 728,080 | 538,432 | 37 | 38 | 28 |
| Human | 9 | 109,434,656 | 32 | 524,935 | 484,640 | 38 | 38 | 15 |
| Human | 10 | 128,196,704 | 30 | 726,502 | 478,592 | 39 | 39 | 17 |
| Human | 11 | 130,274,560 | 50 | 564,057 | 462,864 | 38 | 37 | 22 |
| Human | 12 | 129,843,424 | 47 | 741,947 | 488,288 | 38 | 38 | 27 |
| Human | 13 | 94,957,600 | 45 | 691,877 | 447,584 | 37 | 37 | 33 |
| Human | 14 | 86,964,576 | 36 | 632,915 | 518,720 | 38 | 38 | 26 |
| Human | 15 | 77,770,144 | 20 | 459,477 | 417,728 | 39 | 39 | 12 |
| Human | 16 | 74,960,800 | 23 | 535,928 | 488,608 | 42 | 41 | 16 |
| Human | 17 | 75,513,088 | 21 | 535,538 | 490,656 | 42 | 40 | 15 |
| Human | 18 | 74,291,328 | 34 | 555,501 | 467,168 | 38 | 37 | 25 |
| Human | 19 | 54,533,856 | 8 | 455,012 | 429,824 | 45 | 44 | 7 |
| Human | 20 | 59,266,464 | 15 | 863,979 | 419,232 | 43 | 43 | 22 |
| Human | 21 | 33,211,648 | 19 | 522,050 | 504,064 | 38 | 36 | 30 |
| Human | 22 | 33,725,632 | 7 | 383,799 | 387,200 | 45 | 46 | 8 |
| Human | X | 135,172,992 | 53 | 825,437 | 570,176 | 38 | 37 | 32 |
| Human | Y | 18,186,240 | 6 | 390,224 | 365,552 | 37 | 37 | 13 |
| Chimpanzee | 1 | 217,189,792 | 74 | 589,182 | 411,680 | 40 | 39 | 20 |
| Chimpanzee | 2A | 105,873,440 | 29 | 701,657 | 509,888 | 38 | 38 | 19 |
| Chimpanzee | 2B | 127,869,088 | 57 | 611,889 | 546,752 | 37 | 37 | 27 |
| Chimpanzee | 3 | 194,962,656 | 92 | 633,810 | 476,032 | 38 | 38 | 30 |
| Chimpanzee | 4 | 186,955,680 | 93 | 747,935 | 478,912 | 37 | 37 | 37 |
| Chimpanzee | 5 | 175,225,344 | 81 | 645,786 | 470,912 | 38 | 37 | 30 |
| Chimpanzee | 6 | 164,697,664 | 67 | 697,120 | 487,904 | 38 | 38 | 28 |
| Chimpanzee | 7 | 151,069,600 | 57 | 704,608 | 520,064 | 38 | 37 | 27 |
| Chimpanzee | 8 | 138,150,976 | 54 | 616,786 | 487,904 | 38 | 38 | 24 |
| Chimpanzee | 9 | 109,295,936 | 40 | 628,938 | 516,576 | 39 | 38 | 23 |
| Chimpanzee | 10 | 125,696,576 | 32 | 711,916 | 451,632 | 39 | 40 | 18 |
| Chimpanzee | 11 | 123,596,640 | 38 | 663,580 | 496,960 | 37 | 37 | 20 |
| Chimpanzee | 12 | 129,867,936 | 46 | 655,281 | 504,656 | 38 | 37 | 23 |
| Chimpanzee | 13 | 87,794,784 | 45 | 698,930 | 500,736 | 37 | 37 | 36 |
| Chimpanzee | 14 | 86,251,392 | 39 | 610,530 | 520,448 | 38 | 37 | 28 |
| Chimpanzee | 15 | 76,971,936 | 22 | 630,282 | 399,040 | 40 | 40 | 18 |
| Chimpanzee | 16 | 74,505,664 | 24 | 505,948 | 416,096 | 43 | 42 | 16 |
| Chimpanzee | 17 | 73,429,760 | 19 | 569,142 | 538,144 | 41 | 40 | 15 |
| Chimpanzee | 18 | 74,181,056 | 31 | 601,498 | 457,056 | 38 | 38 | 25 |
| Chimpanzee | 19 | 51,997,056 | 6 | 638,651 | 605,408 | 47 | 48 | 7 |
| Chimpanzee | 20 | 58,102,080 | 12 | 1,108,699 | 647,152 | 43 | 43 | 23 |
| Chimpanzee | 21 | 32,706,016 | 19 | 561,907 | 566,624 | 38 | 36 | 33 |
| Chimpanzee | 22 | 32,341,152 | 8 | 391,420 | 346,224 | 44 | 45 | 10 |
| Chimpanzee | X | 130,929,824 | 64 | 699,055 | 468,192 | 38 | 37 | 34 |
| Chimpanzee | Y | 22,691,168 | 3 | 488,256 | 538,080 | 38 | 38 | 6 |
| Orangutan | 1 | 216,060,736 | 72 | 593,614 | 412,848 | 39 | 38 | 20 |
| Orangutan | 2A | 104,616,320 | 35 | 658,416 | 542,624 | 38 | 38 | 22 |
| Orangutan | 2B | 126,496,928 | 56 | 609,787 | 527,648 | 37 | 37 | 27 |
| Orangutan | 3 | 190,355,264 | 94 | 635,062 | 478,976 | 38 | 37 | 31 |
| Orangutan | 4 | 186,135,648 | 92 | 745,335 | 485,104 | 37 | 37 | 37 |
| Orangutan | 5 | 172,726,976 | 81 | 688,924 | 489,600 | 38 | 37 | 32 |
| Orangutan | 6 | 164,088,672 | 78 | 705,700 | 480,480 | 38 | 37 | 34 |
| Orangutan | 7 | 145,686,624 | 57 | 646,302 | 510,912 | 38 | 37 | 25 |
| Orangutan | 8 | 140,594,368 | 59 | 680,306 | 517,632 | 38 | 38 | 29 |
| Orangutan | 9 | 109,204,832 | 37 | 651,082 | 487,584 | 39 | 38 | 22 |
| Orangutan | 10 | 124,371,648 | 44 | 629,097 | 464,416 | 40 | 40 | 22 |
| Orangutan | 11 | 124,382,944 | 44 | 611,287 | 537,952 | 38 | 38 | 22 |
| Orangutan | 12 | 128,777,280 | 52 | 587,785 | 436,112 | 38 | 38 | 24 |
| Orangutan | 13 | 94,686,048 | 43 | 835,039 | 487,392 | 37 | 36 | 38 |
| Orangutan | 14 | 86,577,920 | 39 | 613,088 | 490,464 | 39 | 38 | 28 |
| Orangutan | 15 | 75,961,056 | 19 | 453,250 | 399,040 | 40 | 40 | 11 |
| Orangutan | 16 | 70,802,048 | 21 | 519,532 | 464,576 | 42 | 41 | 15 |
| Orangutan | 17 | 67,081,376 | 12 | 699,157 | 509,328 | 42 | 41 | 13 |
| Orangutan | 18 | 73,515,712 | 34 | 590,891 | 492,384 | 38 | 38 | 27 |
| Orangutan | 19 | 51,367,232 | 7 | 509,646 | 523,072 | 49 | 51 | 7 |
| Orangutan | 20 | 58,061,408 | 16 | 540,964 | 470,080 | 41 | 40 | 15 |
| Orangutan | 21 | 33,052,576 | 18 | 557,920 | 444,544 | 37 | 37 | 30 |
| Orangutan | 22 | 30,217,376 | 5 | 415,264 | 398,336 | 44 | 45 | 7 |
| Orangutan | X | 148,146,336 | 69 | 714,182 | 566,656 | 38 | 37 | 33 |
| Mouse | 1 | 191,477,376 | 107 | 769,875 | 534,880 | 40 | 39 | 43 |
| Mouse | 2 | 178,392,032 | 98 | 591,873 | 447,008 | 41 | 41 | 33 |
| Mouse | 3 | 156,393,856 | 100 | 606,653 | 503,344 | 39 | 39 | 39 |
| Mouse | 4 | 151,886,784 | 82 | 591,976 | 444,640 | 40 | 39 | 32 |
| Mouse | 5 | 147,721,152 | 90 | 654,502 | 510,656 | 41 | 40 | 40 |
| Mouse | 6 | 146,316,992 | 90 | 630,789 | 446,112 | 40 | 39 | 39 |
| Mouse | 7 | 141,878,176 | 56 | 768,581 | 572,944 | 42 | 41 | 30 |
| Mouse | 8 | 124,796,736 | 71 | 626,946 | 522,976 | 41 | 40 | 36 |
| Mouse | 9 | 120,720,160 | 52 | 642,639 | 500,864 | 41 | 42 | 28 |
| Mouse | 10 | 126,847,808 | 76 | 638,207 | 431,200 | 40 | 39 | 38 |
| Mouse | 11 | 118,743,520 | 51 | 564,761 | 447,968 | 43 | 42 | 24 |
| Mouse | 12 | 117,459,264 | 49 | 698,274 | 514,432 | 41 | 39 | 29 |
| Mouse | 13 | 116,370,848 | 61 | 629,834 | 466,624 | 41 | 40 | 33 |
| Mouse | 14 | 121,635,264 | 51 | 733,000 | 576,224 | 41 | 41 | 31 |
| Mouse | 15 | 100,439,936 | 52 | 684,709 | 480,928 | 41 | 40 | 35 |
| Mouse | 16 | 95,004,896 | 45 | 603,703 | 436,640 | 40 | 40 | 29 |
| Mouse | 17 | 91,898,144 | 43 | 764,906 | 508,832 | 41 | 41 | 36 |
| Mouse | 18 | 87,600,032 | 51 | 582,810 | 496,192 | 41 | 41 | 34 |
| Mouse | 19 | 58,142,176 | 32 | 520,124 | 379,760 | 41 | 40 | 29 |
| Mouse | X | 162,080,832 | 55 | 828,607 | 547,744 | 39 | 39 | 28 |
| Mouse | Y | 2,702,496 | 0 | 0 | 0 | 0 | 0 | 0 |
| Rat | 1 | 242,568,192 | 115 | 687,019 | 543,040 | 42 | 41 | 33 |
| Rat | 2 | 235,352,352 | 130 | 651,793 | 483,744 | 40 | 39 | 36 |
| Rat | 3 | 157,225,152 | 93 | 588,336 | 497,984 | 41 | 40 | 35 |
| Rat | 4 | 172,528,416 | 115 | 613,714 | 473,472 | 40 | 39 | 41 |
| Rat | 5 | 157,560,736 | 85 | 660,696 | 484,128 | 41 | 40 | 36 |
| Rat | 6 | 134,552,608 | 74 | 653,059 | 493,824 | 40 | 39 | 36 |
| Rat | 7 | 131,647,424 | 71 | 571,552 | 479,392 | 41 | 40 | 31 |
| Rat | 8 | 118,069,792 | 57 | 535,258 | 427,776 | 41 | 40 | 26 |
| Rat | 9 | 104,712,416 | 61 | 697,485 | 593,984 | 41 | 41 | 41 |
| Rat | 10 | 101,026,080 | 37 | 564,106 | 439,584 | 43 | 43 | 21 |
| Rat | 11 | 81,764,224 | 54 | 622,146 | 432,048 | 40 | 39 | 41 |
| Rat | 12 | 41,216,160 | 14 | 664,475 | 539,472 | 46 | 46 | 23 |
| Rat | 13 | 102,334,624 | 48 | 831,471 | 472,592 | 41 | 40 | 39 |
| Rat | 14 | 100,801,632 | 64 | 548,399 | 432,432 | 40 | 39 | 35 |
| Rat | 15 | 98,895,328 | 52 | 552,156 | 456,544 | 41 | 41 | 29 |
| Rat | 16 | 81,355,456 | 50 | 547,438 | 448,096 | 41 | 40 | 34 |
| Rat | 17 | 87,674,496 | 37 | 824,084 | 542,656 | 42 | 41 | 35 |
| Rat | 18 | 79,668,352 | 45 | 604,289 | 438,656 | 41 | 40 | 34 |
| Rat | 19 | 53,394,240 | 20 | 739,611 | 540,528 | 43 | 42 | 28 |
| Rat | 20 | 49,307,744 | 33 | 542,802 | 471,072 | 42 | 41 | 36 |
| Rat | X | 145,397,312 | 62 | 693,109 | 538,416 | 40 | 39 | 30 |
| Horse | 1 | 183,561,792 | 60 | 481,170 | 376,064 | 39 | 39 | 16 |
| Horse | 2 | 118,957,504 | 36 | 674,446 | 500,752 | 37 | 37 | 20 |
| Horse | 3 | 118,104,864 | 59 | 599,087 | 465,568 | 38 | 37 | 30 |
| Horse | 4 | 107,397,024 | 44 | 576,561 | 469,360 | 37 | 37 | 24 |
| Horse | 5 | 97,742,496 | 26 | 490,868 | 376,144 | 39 | 38 | 13 |
| Horse | 6 | 83,857,984 | 35 | 504,430 | 408,352 | 38 | 38 | 21 |
| Horse | 7 | 96,472,384 | 29 | 568,073 | 434,304 | 38 | 37 | 17 |
| Horse | 8 | 92,896,064 | 30 | 522,064 | 423,168 | 39 | 38 | 17 |
| Horse | 9 | 82,750,176 | 31 | 712,785 | 428,768 | 38 | 37 | 27 |
| Horse | 10 | 82,681,984 | 27 | 600,955 | 412,768 | 38 | 38 | 20 |
| Horse | 11 | 60,492,896 | 9 | 495,164 | 510,112 | 40 | 38 | 7 |
| Horse | 12 | 31,940,768 | 9 | 582,688 | 458,688 | 38 | 38 | 16 |
| Horse | 13 | 41,521,568 | 3 | 455,488 | 462,272 | 42 | 41 | 3 |
| Horse | 14 | 92,877,376 | 30 | 577,279 | 383,024 | 38 | 37 | 19 |
| Horse | 15 | 90,790,016 | 25 | 551,672 | 500,704 | 38 | 38 | 15 |
| Horse | 16 | 86,752,160 | 27 | 640,219 | 428,800 | 39 | 39 | 20 |
| Horse | 17 | 80,151,872 | 34 | 902,855 | 662,688 | 37 | 37 | 38 |
| Horse | 18 | 81,597,248 | 38 | 557,850 | 445,504 | 37 | 37 | 26 |
| Horse | 19 | 59,453,792 | 18 | 545,204 | 376,544 | 38 | 37 | 17 |
| Horse | 20 | 63,448,096 | 28 | 546,227 | 522,064 | 39 | 39 | 24 |
| Horse | 21 | 57,021,056 | 29 | 604,407 | 417,024 | 38 | 39 | 31 |
| Horse | 22 | 49,278,304 | 12 | 551,856 | 456,928 | 41 | 40 | 13 |
| Horse | 23 | 55,051,296 | 23 | 497,699 | 436,864 | 39 | 39 | 21 |
| Horse | 24 | 45,792,672 | 8 | 648,248 | 516,688 | 40 | 40 | 11 |
| Horse | 25 | 38,841,888 | 8 | 492,800 | 425,680 | 40 | 39 | 10 |
| Horse | 26 | 41,476,928 | 25 | 671,946 | 527,296 | 37 | 36 | 41 |
| Horse | 27 | 39,469,792 | 19 | 480,148 | 371,456 | 37 | 37 | 23 |
| Horse | 28 | 45,531,360 | 11 | 749,143 | 728,704 | 39 | 37 | 18 |
| Horse | 29 | 33,264,096 | 9 | 421,454 | 398,528 | 39 | 39 | 11 |
| Horse | 30 | 29,835,520 | 13 | 487,490 | 402,144 | 38 | 38 | 21 |
| Horse | 31 | 24,827,520 | 10 | 548,198 | 439,472 | 40 | 41 | 22 |
| Horse | X | 121,614,432 | 46 | 663,787 | 482,352 | 37 | 36 | 25 |
| Dog | 1 | 122,070,560 | 26 | 686,801 | 418,480 | 38 | 38 | 15 |
| Dog | 2 | 84,986,272 | 19 | 687,352 | 502,208 | 41 | 39 | 15 |
| Dog | 3 | 91,547,360 | 33 | 506,649 | 407,840 | 37 | 36 | 18 |
| Dog | 4 | 88,054,560 | 28 | 782,579 | 420,992 | 37 | 37 | 25 |
| Dog | 5 | 88,556,640 | 15 | 457,199 | 385,024 | 41 | 40 | 8 |
| Dog | 6 | 77,066,624 | 19 | 547,885 | 463,776 | 39 | 40 | 14 |
| Dog | 7 | 80,608,000 | 25 | 526,138 | 477,088 | 38 | 38 | 16 |
| Dog | 8 | 74,071,520 | 25 | 748,805 | 471,424 | 38 | 37 | 25 |
| Dog | 9 | 60,688,608 | 10 | 580,602 | 461,376 | 42 | 40 | 10 |
| Dog | 10 | 68,960,640 | 18 | 587,182 | 555,264 | 39 | 39 | 15 |
| Dog | 11 | 74,159,296 | 29 | 596,307 | 444,640 | 38 | 37 | 23 |
| Dog | 12 | 72,323,808 | 29 | 744,151 | 548,608 | 36 | 36 | 30 |
| Dog | 13 | 62,975,520 | 22 | 720,598 | 428,352 | 36 | 36 | 25 |
| Dog | 14 | 60,715,328 | 31 | 569,267 | 473,696 | 37 | 36 | 29 |
| Dog | 15 | 63,855,456 | 20 | 472,774 | 411,280 | 37 | 36 | 15 |
| Dog | 16 | 59,207,872 | 18 | 459,922 | 397,232 | 38 | 37 | 14 |
| Dog | 17 | 64,090,304 | 14 | 608,409 | 434,928 | 37 | 36 | 13 |
| Dog | 18 | 55,531,008 | 18 | 517,115 | 415,664 | 38 | 37 | 17 |
| Dog | 19 | 53,607,136 | 24 | 585,656 | 455,952 | 36 | 36 | 26 |
| Dog | 20 | 57,839,648 | 16 | 492,046 | 426,720 | 38 | 38 | 14 |
| Dog | 21 | 50,623,104 | 17 | 638,199 | 478,752 | 39 | 38 | 21 |
| Dog | 22 | 61,283,552 | 27 | 968,640 | 552,576 | 36 | 35 | 43 |
| Dog | 23 | 52,169,408 | 13 | 522,801 | 514,272 | 38 | 38 | 13 |
| Dog | 24 | 47,496,224 | 13 | 671,729 | 636,576 | 41 | 41 | 18 |
| Dog | 25 | 51,352,384 | 11 | 798,985 | 537,696 | 38 | 38 | 17 |
| Dog | 26 | 38,804,896 | 10 | 675,027 | 670,848 | 44 | 45 | 17 |
| Dog | 27 | 45,725,984 | 14 | 583,335 | 487,648 | 37 | 37 | 18 |
| Dog | 28 | 40,985,856 | 12 | 467,765 | 420,480 | 40 | 40 | 14 |
| Dog | 29 | 41,709,440 | 16 | 834,226 | 424,096 | 37 | 37 | 32 |
| Dog | 30 | 40,042,528 | 12 | 489,843 | 416,816 | 37 | 37 | 15 |
| Dog | 31 | 39,763,008 | 19 | 647,207 | 534,176 | 36 | 35 | 31 |
| Dog | 32 | 38,755,040 | 16 | 515,538 | 447,760 | 36 | 36 | 21 |
| Dog | 33 | 31,238,496 | 9 | 708,181 | 456,544 | 36 | 36 | 20 |
| Dog | 34 | 41,922,592 | 17 | 510,744 | 461,088 | 37 | 37 | 21 |
| Dog | 35 | 26,416,608 | 3 | 462,699 | 429,088 | 40 | 38 | 5 |
| Dog | 36 | 30,684,608 | 4 | 652,432 | 691,920 | 36 | 36 | 9 |
| Dog | 37 | 30,741,600 | 13 | 622,902 | 389,792 | 37 | 37 | 26 |
| Dog | 38 | 23,779,040 | 8 | 815,520 | 482,432 | 36 | 36 | 27 |
| Dog | X | 123,181,696 | 48 | 765,985 | 468,928 | 38 | 38 | 30 |
| Pig | 1 | 291,977,152 | 142 | 574,317 | 456,608 | 38 | 38 | 28 |
| Pig | 2 | 138,150,912 | 52 | 492,794 | 444,944 | 38 | 37 | 19 |
| Pig | 3 | 121,501,024 | 30 | 543,831 | 436,528 | 39 | 38 | 13 |
| Pig | 4 | 134,999,424 | 51 | 566,360 | 464,256 | 39 | 38 | 21 |
| Pig | 5 | 98,837,824 | 42 | 517,672 | 418,272 | 39 | 38 | 22 |
| Pig | 6 | 121,210,944 | 30 | 531,193 | 465,200 | 42 | 39 | 13 |
| Pig | 7 | 135,052,864 | 42 | 535,179 | 486,608 | 41 | 40 | 17 |
| Pig | 8 | 117,775,104 | 58 | 689,094 | 491,904 | 37 | 37 | 34 |
| Pig | 9 | 130,657,248 | 56 | 526,493 | 424,480 | 38 | 38 | 23 |
| Pig | 10 | 65,775,680 | 17 | 558,889 | 433,120 | 42 | 42 | 14 |
| Pig | 11 | 78,558,880 | 32 | 737,130 | 468,928 | 37 | 37 | 30 |
| Pig | 12 | 56,502,336 | 14 | 505,509 | 459,248 | 42 | 40 | 13 |
| Pig | 13 | 142,767,264 | 66 | 655,974 | 445,808 | 38 | 38 | 30 |
| Pig | 14 | 147,417,504 | 45 | 531,356 | 435,584 | 42 | 41 | 16 |
| Pig | 15 | 132,148,704 | 70 | 558,440 | 433,632 | 38 | 38 | 30 |
| Pig | 16 | 76,457,888 | 26 | 590,807 | 492,608 | 37 | 37 | 20 |
| Pig | 17 | 63,427,904 | 22 | 553,846 | 505,840 | 41 | 41 | 19 |
| Pig | 18 | 53,590,720 | 20 | 549,448 | 387,120 | 40 | 39 | 21 |
| Pig | X | 124,471,488 | 57 | 531,409 | 474,368 | 38 | 37 | 24 |
| Cow | 1 | 151,811,296 | 94 | 681,180 | 513,888 | 39 | 38 | 42 |
| Cow | 2 | 135,113,632 | 75 | 702,233 | 558,272 | 39 | 39 | 39 |
| Cow | 3 | 119,266,624 | 50 | 551,004 | 446,704 | 39 | 39 | 23 |
| Cow | 4 | 117,710,720 | 64 | 694,168 | 507,344 | 39 | 39 | 38 |
| Cow | 5 | 117,624,064 | 56 | 687,241 | 495,440 | 39 | 39 | 33 |
| Cow | 6 | 113,387,872 | 71 | 798,067 | 585,888 | 39 | 39 | 50 |
| Cow | 7 | 106,864,480 | 47 | 646,365 | 500,128 | 39 | 38 | 28 |
| Cow | 8 | 108,989,536 | 47 | 672,788 | 467,552 | 40 | 40 | 29 |
| Cow | 9 | 102,186,560 | 57 | 673,462 | 531,840 | 39 | 39 | 38 |
| Cow | 10 | 100,506,688 | 38 | 697,664 | 562,720 | 40 | 40 | 26 |
| Cow | 11 | 103,892,640 | 39 | 579,673 | 474,368 | 40 | 39 | 22 |
| Cow | 12 | 80,766,656 | 42 | 815,278 | 517,824 | 39 | 39 | 42 |
| Cow | 13 | 79,882,464 | 29 | 496,908 | 425,696 | 42 | 41 | 18 |
| Cow | 16 | 72,475,040 | 25 | 595,112 | 485,664 | 39 | 39 | 21 |
| Cow | 17 | 71,031,328 | 33 | 760,412 | 552,288 | 40 | 39 | 35 |
| Cow | 18 | 61,271,040 | 11 | 497,876 | 362,272 | 41 | 41 | 9 |
| Cow | 19 | 60,848,224 | 16 | 443,036 | 364,848 | 44 | 44 | 12 |
| Cow | 20 | 70,909,696 | 32 | 883,445 | 497,472 | 39 | 39 | 40 |
| Cow | 21 | 65,176,704 | 16 | 896,812 | 634,656 | 40 | 39 | 22 |
| Cow | 22 | 58,557,152 | 21 | 687,695 | 449,632 | 40 | 40 | 25 |
| Cow | 23 | 49,505,216 | 20 | 572,598 | 484,048 | 41 | 41 | 23 |
| Cow | 24 | 61,194,496 | 34 | 620,969 | 562,000 | 40 | 39 | 35 |
| Cow | 25 | 41,451,456 | 6 | 565,157 | 572,256 | 44 | 44 | 8 |
| Cow | 26 | 48,502,016 | 19 | 582,781 | 497,760 | 41 | 40 | 23 |
| Cow | 27 | 45,288,160 | 21 | 649,464 | 439,808 | 40 | 39 | 30 |
| Cow | 28 | 43,186,240 | 20 | 635,218 | 395,120 | 40 | 41 | 29 |
| Cow | 29 | 48,141,824 | 15 | 771,072 | 671,104 | 40 | 39 | 24 |
| Cow | X | 83,036,672 | 30 | 622,838 | 480,608 | 39 | 39 | 23 |
| Cow | Y | 38,719,968 | 2 | 424,416 | 424,416 | 39 | 39 | 2 |
| Opossum | 1 | 733,412,928 | 336 | 787,673 | 532,304 | 37 | 37 | 36 |
| Opossum | 2 | 527,981,664 | 265 | 785,670 | 530,464 | 37 | 37 | 39 |
| Opossum | 3 | 515,087,168 | 281 | 728,012 | 516,576 | 36 | 36 | 40 |
| Opossum | 4 | 422,697,408 | 223 | 744,941 | 536,864 | 37 | 36 | 39 |
| Opossum | 5 | 298,042,848 | 158 | 633,582 | 465,744 | 36 | 36 | 34 |
| Opossum | 6 | 284,524,512 | 124 | 767,908 | 510,640 | 37 | 37 | 33 |
| Opossum | 7 | 254,715,584 | 146 | 831,837 | 475,696 | 36 | 36 | 48 |
| Opossum | 8 | 303,158,720 | 153 | 708,780 | 505,472 | 37 | 37 | 36 |
| Opossum | X | 72,972,128 | 19 | 516,205 | 455,040 | 39 | 38 | 13 |
| Chicken | 1 | 195,191,328 | 70 | 561,154 | 378,704 | 39 | 38 | 20 |
| Chicken | 2 | 150,357,856 | 55 | 517,229 | 423,488 | 38 | 38 | 19 |
| Chicken | 3 | 110,204,896 | 31 | 548,320 | 405,664 | 38 | 38 | 15 |
| Chicken | 4 | 90,936,160 | 24 | 763,475 | 478,720 | 39 | 39 | 20 |
| Chicken | 5 | 59,512,608 | 18 | 599,127 | 514,832 | 41 | 40 | 18 |
| Chicken | 6 | 35,175,936 | 8 | 364,556 | 344,320 | 40 | 40 | 8 |
| Chicken | 7 | 36,212,288 | 6 | 671,936 | 440,560 | 40 | 40 | 11 |
| Chicken | 8 | 28,570,208 | 9 | 589,049 | 451,328 | 38 | 38 | 19 |
| Chicken | 9 | 23,541,728 | 8 | 441,980 | 440,736 | 40 | 39 | 15 |
| Chicken | 10 | 20,484,704 | 4 | 383,280 | 369,888 | 45 | 45 | 7 |
| Chicken | 11 | 20,811,168 | 7 | 388,539 | 377,952 | 39 | 39 | 13 |
| Chicken | 12 | 19,573,280 | 3 | 576,192 | 640,544 | 42 | 42 | 9 |
| Chicken | 13 | 17,932,000 | 1 | 1,065,024 | 1,065,024 | 43 | 43 | 6 |
| Chicken | 14 | 15,306,464 | 3 | 442,507 | 427,616 | 44 | 43 | 9 |
| Chicken | 15 | 12,585,504 | 4 | 612,520 | 586,384 | 43 | 43 | 19 |
| Chicken | 16 | 405,312 | 0 | 0 | 0 | 0 | 0 | 0 |
| Chicken | 17 | 10,337,984 | 1 | 324,448 | 324,448 | 50 | 50 | 3 |
| Chicken | 18 | 10,513,248 | 1 | 358,560 | 358,560 | 46 | 46 | 3 |
| Chicken | 19 | 9,570,432 | 0 | 0 | 0 | 0 | 0 | 0 |
| Chicken | 20 | 13,560,512 | 4 | 413,768 | 431,760 | 43 | 44 | 12 |
| Chicken | 21 | 6,656,928 | 1 | 300,928 | 300,928 | 47 | 47 | 5 |
| Chicken | 22 | 3,809,632 | 1 | 301,696 | 301,696 | 43 | 43 | 8 |
| Chicken | 23 | 5,248,000 | 0 | 0 | 0 | 0 | 0 | 0 |
| Chicken | 24 | 6,138,368 | 2 | 500,064 | 500,064 | 50 | 50 | 16 |
| Chicken | 25 | 1,367,840 | 0 | 0 | 0 | 0 | 0 | 0 |
| Chicken | 26 | 4,830,272 | 1 | 482,528 | 482,528 | 51 | 51 | 10 |
| Chicken | 27 | 4,538,432 | 1 | 649,984 | 649,984 | 51 | 51 | 14 |
| Chicken | 28 | 3,711,296 | 0 | 0 | 0 | 0 | 0 | 0 |
| Chicken | W | 233,824 | 0 | 0 | 0 | 0 | 0 | 0 |
| Chicken | Z | 67,535,552 | 18 | 579,303 | 548,432 | 39 | 39 | 15 |
