# Supplementary material for: A Comparative Study and a Phylogenetic Exploration of the Compositional Architectures of Mammalian Nuclear Genomes
Source: PLoS Comput Biol. 2014 Nov 6;10(11):e1003925. doi: 10.1371/journal.pcbi.1003925 (PMC4222635; doi:10.1371/journal.pcbi.1003925)
Supplement: Table S2 — Categories of compositional domains by length and GC content. (DOC) [file pcbi.1003925.s009.doc]

**Table S2. Categories of compositional domains by length and GC content**

| Species | Domain type | GC content | Short (10^3^–10^4^ bp) | % | Medium-short (10^4^–10^5^ bp) | % | Medium-long (10^5^–10^6^ bp) | % | Long (10^6^–10^7^ bp) | % | Total for type | % |
| --- | --- | --- | --- | --- | --- | --- | --- | --- | --- | --- | --- | --- |
| Human | Compositionally nonhomogeneous domains | GC poor | 7,911 | 7 | 3,512 | 3 | 202 | 0 | 3 | 0.00 | 11,628 | 11 |
| Human | Compositionally nonhomogeneous domains | GC rich | 13,921 | 13 | 6,847 | 6 | 587 | 1 | 9 | 0.01 | 21,364 | 20 |
| Human | Compositionally nonhomogeneous domains | GC poor+rich | 21,832 | 20 | 10,359 | 10 | 789 | 1 | 12 | 0.01 | 32,992 | 31 |
| Human | Compositionally homogeneous domains | GC poor | 20,242 | 19 | 14,498 | 13 | 2,848 | 3 | 133 | 0.12 | 37,721 | 35 |
| Human | Compositionally homogeneous domains | GC rich | 22,202 | 21 | 13,638 | 13 | 1,004 | 1 | 14 | 0.01 | 36,858 | 34 |
| Human | Compositionally homogeneous domains | GC poor+rich | 42,444 | 39 | 28,136 | 26 | 3,852 | 4 | 147 | 0.14 | 74,579 | 69 |
| Human | All domains | GC poor | 28,153 | 26 | 18,010 | 17 | 3,050 | 3 | 136 | 0.13 | 49,349 | 46 |
| Human | All domains | GC rich | 36,123 | 34 | 20,485 | 19 | 1,591 | 1 | 23 | 0.02 | 58,222 | 54 |
| Human | All domains | GC poor+rich | 64,276 | 60 | 38,495 | 36 | 4,641 | 4 | 159 | 0.15 | 107,571 | 100 |
| Chimpanzee | Compositionally nonhomogeneous domains | GC poor | 8,142 | 8 | 3,710 | 3 | 233 | 0 | 1 | 0.00 | 12,086 | 11 |
| Chimpanzee | Compositionally nonhomogeneous domains | GC rich | 14,612 | 14 | 6,934 | 6 | 547 | 1 | 8 | 0.01 | 22,101 | 21 |
| Chimpanzee | Compositionally nonhomogeneous domains | GC poor+rich | 22,754 | 21 | 10,644 | 10 | 780 | 1 | 9 | 0.01 | 34,187 | 32 |
| Chimpanzee | Compositionally homogeneous domains | GC poor | 19,621 | 18 | 14,653 | 14 | 2,784 | 3 | 133 | 0.12 | 37,191 | 35 |
| Chimpanzee | Compositionally homogeneous domains | GC rich | 21,605 | 20 | 13,396 | 12 | 964 | 1 | 16 | 0.01 | 35,981 | 34 |
| Chimpanzee | Compositionally homogeneous domains | GC poor+rich | 41,226 | 38 | 28,049 | 26 | 3,748 | 3 | 149 | 0.14 | 73,172 | 68 |
| Chimpanzee | All domains | GC poor | 27,763 | 26 | 18,363 | 17 | 3,017 | 3 | 134 | 0.12 | 49,277 | 46 |
| Chimpanzee | All domains | GC rich | 36,217 | 34 | 20,330 | 19 | 1,511 | 1 | 24 | 0.02 | 58,082 | 54 |
| Chimpanzee | All domains | GC poor+rich | 63,980 | 60 | 38,693 | 36 | 4,528 | 4 | 158 | 0.15 | 107,359 | 100 |
| Orangutan | Compositionally nonhomogeneous domains | GC poor | 7,946 | 8 | 3,703 | 4 | 206 | 0 | 4 | 0.00 | 11,859 | 11 |
| Orangutan | Compositionally nonhomogeneous domains | GC rich | 14,013 | 13 | 6,864 | 6 | 561 | 1 | 7 | 0.01 | 21,445 | 20 |
| Orangutan | Compositionally nonhomogeneous domains | GC poor+rich | 21,959 | 21 | 10,567 | 10 | 767 | 1 | 11 | 0.01 | 33,304 | 32 |
| Orangutan | Compositionally homogeneous domains | GC poor | 19,722 | 19 | 14,678 | 14 | 2,753 | 3 | 142 | 0.13 | 37,295 | 35 |
| Orangutan | Compositionally homogeneous domains | GC rich | 21,000 | 20 | 13,211 | 13 | 863 | 1 | 15 | 0.01 | 35,089 | 33 |
| Orangutan | Compositionally homogeneous domains | GC poor+rich | 40,722 | 39 | 27,889 | 26 | 3,616 | 3 | 157 | 0.15 | 72,384 | 68 |
| Orangutan | All domains | GC poor | 27,668 | 26 | 18,381 | 17 | 2,959 | 3 | 146 | 0.14 | 49,154 | 47 |
| Orangutan | All domains | GC rich | 35,013 | 33 | 20,075 | 19 | 1,424 | 1 | 22 | 0.02 | 56,534 | 53 |
| Orangutan | All domains | GC poor+rich | 62,681 | 59 | 38,456 | 36 | 4,383 | 4 | 168 | 0.16 | 105,688 | 100 |
| Mouse | Compositionally nonhomogeneous domains | GC poor | 6,554 | 10 | 3,647 | 5 | 299 | 0 | 16 | 0.02 | 10,516 | 16 |
| Mouse | Compositionally nonhomogeneous domains | GC rich | 9,167 | 14 | 5,225 | 8 | 527 | 1 | 5 | 0.01 | 14,924 | 22 |
| Mouse | Compositionally nonhomogeneous domains | GC poor+rich | 15,721 | 23 | 8,872 | 13 | 826 | 1 | 21 | 0.03 | 25,440 | 38 |
| Mouse | Compositionally homogeneous domains | GC poor | 6,672 | 10 | 6,347 | 9 | 2,100 | 3 | 102 | 0.15 | 15,221 | 23 |
| Mouse | Compositionally homogeneous domains | GC rich | 13,096 | 19 | 11,505 | 17 | 1,888 | 3 | 73 | 0.11 | 26,562 | 40 |
| Mouse | Compositionally homogeneous domains | GC poor+rich | 19,768 | 29 | 17,852 | 27 | 3,988 | 6 | 175 | 0.26 | 41,783 | 62 |
| Mouse | All domains | GC poor | 13,226 | 20 | 9,994 | 15 | 2,399 | 4 | 118 | 0.18 | 25,737 | 38 |
| Mouse | All domains | GC rich | 22,263 | 33 | 16,730 | 25 | 2,415 | 4 | 78 | 0.12 | 41,486 | 62 |
| Mouse | All domains | GC poor+rich | 35,489 | 53 | 26,724 | 40 | 4,814 | 7 | 196 | 0.29 | 67,223 | 100 |
| Rat | Compositionally nonhomogeneous domains | GC poor | 5,637 | 9 | 3,017 | 5 | 266 | 0 | 13 | 0.02 | 8,933 | 14 |
| Rat | Compositionally nonhomogeneous domains | GC rich | 9,297 | 15 | 5,444 | 9 | 516 | 1 | 2 | 0.00 | 15,259 | 24 |
| Rat | Compositionally nonhomogeneous domains | GC poor+rich | 14,934 | 24 | 8,461 | 13 | 782 | 1 | 15 | 0.02 | 24,192 | 38 |
| Rat | Compositionally homogeneous domains | GC poor | 6,619 | 10 | 5,420 | 9 | 2,027 | 3 | 102 | 0.16 | 14,168 | 22 |
| Rat | Compositionally homogeneous domains | GC rich | 11,435 | 18 | 11,226 | 18 | 2,064 | 3 | 52 | 0.08 | 24,777 | 39 |
| Rat | Compositionally homogeneous domains | GC poor+rich | 18,054 | 29 | 16,646 | 26 | 4,091 | 6 | 154 | 0.24 | 38,945 | 62 |
| Rat | All domains | GC poor | 12,256 | 19 | 8,437 | 13 | 2,293 | 4 | 115 | 0.18 | 23,101 | 37 |
| Rat | All domains | GC rich | 20,732 | 33 | 16,670 | 26 | 2,580 | 4 | 54 | 0.09 | 40,036 | 63 |
| Rat | All domains | GC poor+rich | 32,988 | 52 | 25,107 | 40 | 4,873 | 8 | 169 | 0.27 | 63,137 | 100 |
| Horse | Compositionally nonhomogeneous domains | GC poor | 6,545 | 6 | 1,866 | 2 | 64 | 0 | - | 0.00 | 8,475 | 8 |
| Horse | Compositionally nonhomogeneous domains | GC rich | 14,751 | 13 | 5,763 | 5 | 191 | 0 | 1 | 0.00 | 20,706 | 18 |
| Horse | Compositionally nonhomogeneous domains | GC poor+rich | 21,296 | 19 | 7,629 | 7 | 255 | 0 | 1 | 0.00 | 29,181 | 26 |
| Horse | Compositionally homogeneous domains | GC poor | 23,282 | 21 | 15,834 | 14 | 2,491 | 2 | 85 | 0.08 | 41,692 | 37 |
| Horse | Compositionally homogeneous domains | GC rich | 25,513 | 23 | 15,203 | 14 | 757 | 1 | 4 | 0.00 | 41,477 | 37 |
| Horse | Compositionally homogeneous domains | GC poor+rich | 48,795 | 43 | 31,037 | 28 | 3,248 | 3 | 89 | 0.08 | 83,169 | 74 |
| Horse | All domains | GC poor | 29,827 | 27 | 17,700 | 16 | 2,555 | 2 | 85 | 0.08 | 50,167 | 45 |
| Horse | All domains | GC rich | 40,264 | 36 | 20,966 | 19 | 948 | 1 | 5 | 0.00 | 62,183 | 55 |
| Horse | All domains | GC poor+rich | 70,091 | 62 | 38,666 | 34 | 3,503 | 3 | 90 | 0.08 | 112,350 | 100 |
| Dog | Compositionally nonhomogeneous domains | GC poor | 6,585 | 6 | 3,086 | 3 | 182 | 0 | 1 | 0.00 | 9,854 | 9 |
| Dog | Compositionally nonhomogeneous domains | GC rich | 15,557 | 15 | 5,999 | 6 | 330 | 0 | 4 | 0.00 | 21,890 | 21 |
| Dog | Compositionally nonhomogeneous domains | GC poor+rich | 22,142 | 21 | 9,085 | 9 | 512 | 0 | 5 | 0.00 | 31,744 | 30 |
| Dog | Compositionally homogeneous domains | GC poor | 18,928 | 18 | 15,750 | 15 | 2,375 | 2 | 69 | 0.07 | 37,122 | 35 |
| Dog | Compositionally homogeneous domains | GC rich | 22,458 | 21 | 12,867 | 12 | 681 | 1 | 13 | 0.01 | 36,019 | 34 |
| Dog | Compositionally homogeneous domains | GC poor+rich | 41,386 | 39 | 28,617 | 27 | 3,056 | 3 | 82 | 0.08 | 73,141 | 70 |
| Dog | All domains | GC poor | 25,513 | 24 | 18,836 | 18 | 2,557 | 2 | 70 | 0.07 | 46,976 | 45 |
| Dog | All domains | GC rich | 38,015 | 36 | 18,866 | 18 | 1,011 | 1 | 17 | 0.02 | 57,909 | 55 |
| Dog | All domains | GC poor+rich | 63,528 | 61 | 37,702 | 36 | 3,568 | 3 | 87 | 0.08 | 104,885 | 100 |
| Pig | Compositionally nonhomogeneous domains | GC poor | 5,549 | 6 | 1,918 | 2 | 68 | 0 | - | 0.00 | 7,535 | 8 |
| Pig | Compositionally nonhomogeneous domains | GC rich | 12,419 | 13 | 5,354 | 6 | 251 | 0 | 1 | 0.00 | 18,025 | 19 |
| Pig | Compositionally nonhomogeneous domains | GC poor+rich | 17,968 | 19 | 7,272 | 8 | 319 | 0 | 1 | 0.00 | 25,560 | 27 |
| Pig | Compositionally homogeneous domains | GC poor | 15,912 | 17 | 12,563 | 13 | 2,276 | 2 | 65 | 0.07 | 30,816 | 32 |
| Pig | Compositionally homogeneous domains | GC rich | 23,822 | 25 | 15,143 | 16 | 1,055 | 1 | 14 | 0.01 | 40,034 | 42 |
| Pig | Compositionally homogeneous domains | GC poor+rich | 39,734 | 41 | 27,706 | 29 | 3,331 | 3 | 79 | 0.08 | 70,850 | 73 |
| Pig | All domains | GC poor | 21,461 | 22 | 14,481 | 15 | 2,344 | 2 | 65 | 0.07 | 38,351 | 40 |
| Pig | All domains | GC rich | 36,241 | 38 | 20,497 | 21 | 1,306 | 1 | 15 | 0.02 | 58,059 | 60 |
| Pig | All domains | GC poor+rich | 57,702 | 60 | 34,978 | 36 | 3,650 | 4 | 80 | 0.08 | 96,410 | 100 |
| Cow | Compositionally nonhomogeneous domains | GC poor | 7,084 | 8 | 2,317 | 3 | 61 | 0 | - | 0.00 | 9,462 | 10 |
| Cow | Compositionally nonhomogeneous domains | GC rich | 11,859 | 13 | 5,049 | 6 | 279 | 0 | 4 | 0.00 | 17,191 | 19 |
| Cow | Compositionally nonhomogeneous domains | GC poor+rich | 18,943 | 21 | 7,366 | 8 | 340 | 0 | 4 | 0.00 | 26,653 | 29 |
| Cow | Compositionally homogeneous domains | GC poor | 12,937 | 14 | 10,103 | 11 | 2,226 | 2 | 131 | 0.14 | 25,397 | 28 |
| Cow | Compositionally homogeneous domains | GC rich | 23,175 | 26 | 14,433 | 16 | 1,201 | 1 | 22 | 0.02 | 38,831 | 43 |
| Cow | Compositionally homogeneous domains | GC poor+rich | 36,112 | 40 | 24,536 | 27 | 3,427 | 4 | 153 | 0.17 | 64,228 | 71 |
| Cow | All domains | GC poor | 20,021 | 22 | 12,420 | 14 | 2,287 | 3 | 131 | 0.14 | 34,859 | 38 |
| Cow | All domains | GC rich | 35,034 | 39 | 19,482 | 21 | 1,480 | 2 | 26 | 0.03 | 56,022 | 62 |
| Cow | All domains | GC poor+rich | 55,055 | 61 | 31,902 | 35 | 3,767 | 4 | 157 | 0.17 | 90,881 | 100 |
| Opossum | Compositionally nonhomogeneous domains | GC poor | 16,781 | 16 | 8,017 | 7 | 575 | 1 | 9 | 0.01 | 25,382 | 24 |
| Opossum | Compositionally nonhomogeneous domains | GC rich | 13,881 | 13 | 4,428 | 4 | 268 | 0 | 4 | 0.00 | 18,581 | 17 |
| Opossum | Compositionally nonhomogeneous domains | GC poor+rich | 30,662 | 29 | 12,445 | 12 | 843 | 1 | 13 | 0.01 | 43,963 | 41 |
| Opossum | Compositionally homogeneous domains | GC poor | 21,102 | 20 | 19,823 | 18 | 4,419 | 4 | 304 | 0.28 | 45,648 | 43 |
| Opossum | Compositionally homogeneous domains | GC rich | 13,017 | 12 | 4,513 | 4 | 213 | 0 | 1 | 0.00 | 17,744 | 17 |
| Opossum | Compositionally homogeneous domains | GC poor+rich | 34,119 | 32 | 24,336 | 23 | 4,632 | 4 | 305 | 0.28 | 63,392 | 59 |
| Opossum | All domains | GC poor | 37,883 | 35 | 27,840 | 26 | 4,994 | 5 | 313 | 0.29 | 71,030 | 66 |
| Opossum | All domains | GC rich | 26,898 | 25 | 8,941 | 8 | 481 | 0 | 5 | 0.00 | 36,325 | 34 |
| Opossum | All domains | GC poor+rich | 64,781 | 60 | 36,781 | 34 | 5,475 | 5 | 318 | 0.30 | 107,355 | 100 |
| Chicken | Compositionally nonhomogeneous domains | GC poor | 1,629 | 4 | 714 | 2 | 60 | 0 | 3 | 0.01 | 2,406 | 6 |
| Chicken | Compositionally nonhomogeneous domains | GC rich | 6,280 | 16 | 2,483 | 6 | 136 | 0 | 4 | 0.01 | 8,903 | 23 |
| Chicken | Compositionally nonhomogeneous domains | GC poor+rich | 7,909 | 20 | 3,197 | 8 | 196 | 0 | 7 | 0.02 | 11,309 | 29 |
| Chicken | Compositionally homogeneous domains | GC poor | 6,520 | 17 | 7,954 | 20 | 989 | 3 | 14 | 0.04 | 15,477 | 39 |
| Chicken | Compositionally homogeneous domains | GC rich | 6,499 | 16 | 5,743 | 15 | 413 | 1 | 9 | 0.02 | 12,664 | 32 |
| Chicken | Compositionally homogeneous domains | GC poor+rich | 13,019 | 33 | 13,697 | 35 | 1,402 | 4 | 23 | 0.06 | 28,141 | 71 |
| Chicken | All domains | GC poor | 8,149 | 21 | 8,668 | 22 | 1,049 | 3 | 17 | 0.04 | 17,883 | 45 |
| Chicken | All domains | GC rich | 12,779 | 32 | 8,226 | 21 | 549 | 1 | 13 | 0.03 | 21,567 | 55 |
| Chicken | All domains | GC poor+rich | 20,928 | 53 | 16,894 | 43 | 1,598 | 4 | 30 | 0.08 | 39,450 | 100 |
