# Supplementary material for: A Comparative Study and a Phylogenetic Exploration of the Compositional Architectures of Mammalian Nuclear Genomes
Source: PLoS Comput Biol. 2014 Nov 6;10(11):e1003925. doi: 10.1371/journal.pcbi.1003925 (PMC4222635; doi:10.1371/journal.pcbi.1003925)
Supplement: Table S3 — List of 49 publications by Professor Giorgio Bernardi and colleagues in which isochores are defined as compositionally homogeneous genomic stretches longer than 300 kb*#. (DOC) [file pcbi.1003925.s010.doc]

**Table S3. List of 49 publications by Professor Giorgio Bernardi and colleagues in which isochores are defined as compositionally homogeneous genomic stretches longer than 300 kb*#**

| Year | Author | Title | Journal | Volume | Pages |
| --- | --- | --- | --- | --- | --- |
| 1983 | Olofsson B, Bernardi G. | The distribution of CR1, and *Alu*-like family of interspersed repeats, in the chicken genome | *Biochimica et Biophysica Acta* | 740 | 339-341 |
| 1983 | Soriano P. Meunier-Rotival M, Bernardi G. | The distribution of interspersed repeats is nonuniform and conserved in the mouse and human genomes | *Proceedings of the National Academy of Sciences USA* | 80 | 1816-1820 |
| 1987 | Mouchiroud D, Fichant G, Bernardi G. | Compositional compartmentalization and gene composition in the genome of vertebrates | *Journal of Molecular Evolution* | 26 | 198-204 |
| 1987 | Salinas J., Zerial M, Filipski J, Crepin M, Bernardi G. | Nonrandom distribution of *MMTV* proviral sequences in the mouse genome | *Nucleic Acids Research* | 15 | 3009-3022 |
| 1988 | Mouchiroud D, Gautier C, Bernardi G. | The compositional distribution of coding sequences and DNA molecules in humans and murids | *Journal of Molecular Evolution* | 27 | 311-320 |
| 1988 | Salinas J, Matassi G, Montero LM, Bernardi G. | Compositional compartmentalization and compositional patterns in the nuclear genomes of plants | *Nucleic Acids Res* | 16 | 4269-4285 |
| 1989 | Bernardi G. | The isochore organization of the human genome | *Annual Review of Genetics* | 23 | 637-661 |
| 1990 | Gardiner K, Aissani B, Bernardi G. | A compositional map of human chromosome 21 | *EMBO Journal* | 9 | 1853-1858 |
| 1991 | de Sario A, Aissani B, Bernardi G. | Compositional properties of telomeric regions from human chromosomes | *FEBS Letters* | 295 | 22-26 |
| 1991 | Mouchiroud, D, D’Onofrio G, Aissani B, Macaya G, Gautier C, Bernardi G. | The distribution of genes in the human genome | *Gene* | 100 | 181-187 |
| 1992 | Bettecken T, Aissani B, Muller CR, Bernardi G. | Compositional mapping of the human dystrophin-encoding gene | *Gene* | 122 | 329-335 |
| 1992 | d’Onofrio G, Bernardi G. | A universal compositional correlation among codon positions | *Gene* | 110 | 81-88 |
| 1992 | Matassi G, Melis R, Kuo KC, Macaya G, Gehrke CW, Bernardi G. | Large-scale methylation patterns in the nuclear genomes of plants | *Gene* | 122 | 239-245 |
| 1992 | Zoubak S, Rynditch A, Bernardi G. | Compositional bimodality and evolution of retroviral genomes | *Gene* | 119 | 207-213 |
| 1993 | Bernardi G. | The vertebrate genome: isochores and evolution | *Molecular Biology and Evolution* | 10 | 186-204 |
| 1993 | Bernardi G. | Genome organization and species formation in vertebrates | *Journal of Molecular Evolution* | 37 | 331-337 |
| 1993 | Bernardi G. | The isochore organization of the human genome and its evolutionary history—a review | *Gene* | 135 | 57-66 |
| 1993 | Bernardi G, Mouchiroud D, Gautier C. | Silent substitutions in mammalian genomes and their evolutionary implications | *Journal of Molecular Evolution* | 37 | 583-589 |
| 1993 | Isacchi A, Bernardi G. | Compositional compartmentalization of the nuclear genomes of *Trypanosoma brucei* and *Trypanosoma equiperdum* | *FEBS Letters* | 335 | 181-183 |
| 1993 | Mouchiroud D, Bernardi G. | Compositional properties of coding sequences and mammalian phylogeny | *Journal of Molecular Evolution* | 37 | 109-116 |
| 1993 | Sabeur G, Macaya G, Kadi F, Bernardi G. | The isochore patterns of mammalian genomes and their phylogenetic implications | *Journal of Molecular Evolution* | 37 | 93-108 |
| 1994 | Zoubak S, Richardson JH, Rynditch A, Hollsberg P, Hafler DA, Boeri E, Lever AM, Bernardi G. | Regional specificity of *HTLV*-I proviral integration in the human genome | *Gene* | 143 | 155-163 |
| 1995 | Bernardi G. | The human genome: organization and evolutionary history | *Annual Review of Genetics* | 29 | 445-476 |
| 1995 | Carels N, Barakat A, Bernardi G. | The gene distribution of the maize genome | *Proceedings of the National Academy of Sciences USA* | 92 | 11057-11060 |
| 1996 | De Sario A, Geigl EM, Palmieri G, d’Urso M, Bernardi G. | A compositional map of human chromosome band Xq28 | *Proceedings of the National Academy of Sciences USA* | 93 | 1298-1302 |
| 1996 | Zoubak S, Clay O, Bernardi G. | The gene distribution of the human genome | *Gene*** | 174 | 95-102 |
| 1997 | Saccone S, Caccio S, Perani P, Andreozzi L, Rapisarda A, Motta S, Bernardi G. | Compositional mapping of mouse chromosomes and identification of the gene-rich regions | *Chromosome Research* | 5 | 293-300 |
| 1998 | Matassi G, Labuda D, Bernardi G. | Distribution of the mammalian-wide interspersed repeats (*MIR*s) in the isochores of the human genome | *FEBS Letters* | 439 | 63-65 |
| 1998 | Rynditch AV, Zoubak S, Tsyba L, Tryapitsina-Guley N, Bernardi G. | The regional integration of retroviral sequences into the mosaic genomes of mammals | *Gene*** | 222 | 16-Jan |
| 1999 | d’Onofrio G, K Jabbari, Musto H, Alvarez-Valin F, Cruveiller S, Bernardi G. | Evolutionary genomics of vertebrates and its implications | *Annals of the New York Academy of Sciences* | 870 | 81-94 |
| 1999 | Pesole G, Bernardi G, Saccone C. | Isochore specificity of AUG initiator context of human genes | *FEBS Letters* | 464 | 60-62 |
| 1999 | Musto H, Romero H, Zavala A, Bernardi G | Compositional correlations in the chicken genome | *Journal of Molecular Evolution*** | 49 | 325-329 |
| 2000 | Bernardi G. | Isochores and the evolutionary genomics of vertebrates | *Gene*** | 241 | 3-17 |
| 2000 | Jabbari K, Bernardi G. | The distribution of genes in the *Drosophila* genome | *Gene*** | 247 | 287-292 |
| 2001 | Bernardi G. | Misunderstandings about isochores. Part 1*** | *Gene*** | 276 | 3-13 |
| 2001 | Clay O, Carels N, Douady C, Macaya G, Bernardi G. | Compositional heterogeneity within and among isochores in mammalian genomes. I. CsCl and sequence analyses | *Gene*** | 276 | 15-24 |
| 2001 | Saccone S, Bernardi G. | Human chromosomal banding by in situ hybridization of isochores | *Methods in Cell Science* | 23 | 7-15 |
| 2001 | Saccone S, Pavlicek A, Federico C, Paces J, Bernardi G. | Genes, isochores and bands in human chromosomes 21 and 22 | *Chromosome Research* | 9 | 533-539 |
| 2002 | Alvarez-Valin F, Lamolle G, Bernardi G. | Isochores, GC3 and mutation biases in the human genome | *Gene*** | 300 | 161-168 |
| 2002 | Pavlicek A, Paces J, Clay O, Bernardi G. | A compact view of isochores in the draft human genome sequence | *FEBS Letters* | 511 | 165-169 |
| 2002 | Saccone S, Federico C, Bernardi G. | Localization of the gene-richest and the gene-poorest isochores in the interphase nuclei of mammals and birds | *Gene*** | 300 | 169-178 |
| 2003 | Cruveiller S, Jabbari K, Clay O, Benardi G. | Compositional features of eukaryotic genomes for checking predicted genes | *Briefings in Bioinformatics* | 4 | 43-52 |
| 2004 | Tsyba L, Rynditch AV, Boeri E, Jabbari K, Bernardi G. | Distribution of HIV-1 in the genomes of AIDS patients | *Cellular and Molecular Life Sciences* | 61 | 721-726 |
| 2004 | Wronka G, Bernardi G, Doerfler W. | Localization of integrated adenovirus DNA in the hamster genome | *Cellular and Molecular Life Sciences* | 61 | 2983-2990 |
| 2006 | Costantini M, Clay O, Auletta F, Bernardi G. | An isochore map of human chromosomes | *Genome Research* | 16 | 536-541 |
| 2006 | Federico, C., C. Scavo, C. D. Cantarella, S. Motta, S. Saccone, and G. Bernardi | Gene-rich and gene-poor chromosomal regions have different locations in the interphase nuclei of cold-blooded vertebrates | *Chromosoma* | 115 | 123-128 |
| 2007 | Bernardi, G | The neoselectionist theory of genome evolution | *Proceedings of the National Academy of Sciences USA* | 104 | 8385-8390 |
| 2007 | Costantini, M., O. Clay, C. Federico, S. Saccone, F. Auletta, and G. Bernardi | Human chromosomal bands: nested structure, high-definition map and molecular basis | *Chromosoma* | 116 | 29-40 |
| 2008 | Costantini M, Bernardi G. | The short-sequence designs of isochores from the human genome | *Proceedings of the National Academy of Sciences USA* | 16 | 13971-13976 |

*Data from Google Scholar.

** From 1996 to 2008, Professor Giorgio Bernardi served as Editor-in-Chief of *Gene*; from 1999 to 2003, he served as Co-Editor-in-Chief of *Journal of Molecular Evolution*.

***As far as we could ascertain, Part 2 was never published.

# In their reply to Cohen, et al. [1], Clay and Bernardi [2] criticized the author’s choice of a 300 kb cutoff as the minimum length of isochores: “*The third attribute, “minimum length” (A3), is roughly appropriate where the authors refer to the literature and use the word “typically” but inappropriate where they subsequently hard-wire a cutoff of 300 kb (the one they emphasize most in the text and abstract) and then discard all shorter segments as “nonisochoric” DNA. This sharp cutoff is apparently not inspired by the literature, and there is no precedent of its use of which we are aware*. “

1. Cohen, N., et al., *GC composition of the human genome: in search of isochores.* Mol Biol Evol, 2005. **22**(5): p. 1260-72.

2. Clay, O. and G. Bernardi, *How Not to Search for Isochores: A Reply to Cohen et al.* Mol Biol Evol, 2005. **22**(12): p. 2315-2317.
